# Limits of Solid Solution and Evolution of Crystal Morphology in (La_1–*x*_RE_*x*_)FeO_3_ Perovskites by Low Temperature Hydrothermal Crystallization

**DOI:** 10.1021/acs.inorgchem.2c04325

**Published:** 2023-03-06

**Authors:** Lu Jia, Matthew D. Lloyd, Martin R. Lees, Limin Huang, Richard I. Walton

**Affiliations:** † Department of Chemistry, University of Warwick, Coventry CV4 7AL, U.K.; ‡ Department of Chemistry, Southern University of Science and Technology, Shenzhen 518055, P. R. China; § Department of Physics, University of Warwick, Coventry CV4 7AL, U.K.

## Abstract

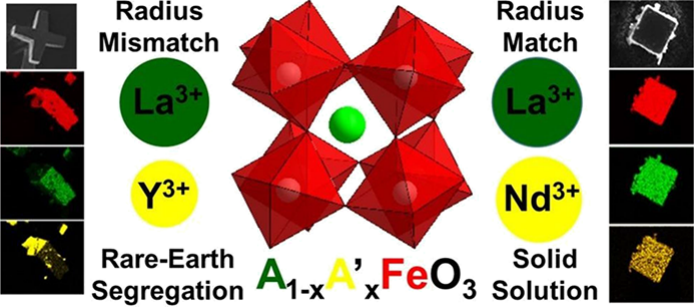

The crystallization of a new series of A-site substituted lanthanum ferrite materials (La_1–*x*_RE_*x*_)FeO_3_ was explored by the hydrothermal method at 240 °C, for rare earth (RE) = Nd, Sm, Gd, Ho, Er, Yb, and Y, with 0 ≤ *x* ≤ 1. The effect of elemental substitution on the morphological, structural, and magnetic properties of the materials was studied using high-resolution powder X-ray diffraction, energy dispersive spectroscopy (EDS) on the scanning electron microscope, Raman spectroscopy, and SQUID magnetometry. If the radius of the La^3+^ and the substituent ions is similar, such as for Nd^3+^, Sm^3+^, and Gd^3+^, homogeneous solid solutions are formed, with the orthorhombic GdFeO_3_-type structure, and a continuous evolution of Raman spectra with composition and distinct magnetic behavior from the end members. When the radius difference between substituents and La^3+^ is large, such as for Ho^3+^, Er^3+^, Yb^3+^, and Y^3+^, then instead of forming solid solutions, crystallization in separate phases is found. However, low levels of element mixing are found and intergrowths of segregated regions give composite particles. In this case, the Raman spectra and magnetic behavior are characteristic of mixtures of phases, while EDS shows distinctive elemental segregation. A-site replacement induces an evolution in the crystallite shape with an increasing amount of substituent ions and this is most evident for RE = Y from cube-shaped crystals seen for LaFeO_3_ to multipodal crystals for (La_1–*x*_Y_*x*_)FeO_3_, providing evidence for a phase-separation-driven evolution of morphology.

## Abstract 2

Hydrothermal synthesis allows direct crystallization of quaternary ferrite perovskites with A-site substitution. Phase homogeneity is achieved for radius-matched A-site cations, but if the A-site cations differ in radius by more than 17%, then phase separation leads to intergrows with distinct crystal morphologies.

## Introduction

The ABX_3_ perovskite structure class encompasses a huge variety of compounds. Many cations of metallic ions in the periodic table can be incorporated into the perovskite structure. Oxides (X = O^2–^) and fluorides (X = F^–^) comprise the vast majority of perovskite compounds, and the perovskite structure is found for many combinations of cations and anions. Chlorides, bromides, hydrides, oxynitrides, and sulfides are also known as perovskite structures.^1^ Rare earth (RE) orthoferrites RE^3+^Fe^3+^O_3_ with distorted perovskite structures have been intensively studied because they are potential multiferroics, which may combine ferromagnetism and ferroelectricity, and they have also been applied in other technical fields, including solid oxide fuel cells,^2^ photovoltaic sensors,^3^ and heterogeneous catalysis.^4^

Distortions of BO_6_ octahedra in RE orthoferrites can be assigned to three mechanisms: distortions of the octahedra, cation displacements within the octahedra, and tilting of the octahedra.^5^ The study of the structural distortions is fundamental because they directly link to the bulk electronic and magnetic properties and even the surface properties of the perovskite oxides, further influencing their application performances.^6^ Previous investigations on lattice distortions resulting from A-site substitution in RE perovskite oxides focused on using a cation with higher valence^7^ and/or with a lower valence in comparison to A^3+^.^8,9^ However, the introduction of the second metal cation into the metal oxides may cause undesired phase separation and structure collapse due to the large ionic radii and charge mismatch of the substituent ion and the original ions. This phenomenon will induce impurities and lead to the limited performance of the perovskite materials for applications.^10,11^ Only a few studies fine-tuned the local structure of lattice oxygen via the A-site substituted by a trivalent ion without significantly changing the crystal structure type of perovskite. Zhang et al. recently synthesized La_1–*x*_Ce_*x*_FeO_3_ (*x* = 0, 0.25, 0.5, 0.75, 1) solid solutions and found that by tuning the degree of the distortion of BO_6_ with A-site substitution, the performance of the samples in chemical looping methane partial oxidation-CO_2_ splitting can be improved and La_0.5_Ce_0.5_FeO_3_ with the maximum distortion of the FeO_6_ octahedra exhibited the best performance among all samples.^12^ Li et al. studied the catalytic performance of different REFeO_3_ (RE = La, Pr, Gd, and Y) perovskites and found that the average Fe–O bond angle, which is decided by the distortion degrees of FeO_6_, is dependent on Fe–O covalency, the O 2p band center, and the charge-transfer energy. The average Fe–O–Fe bond angle between FeO_6_ octahedra is positively related to the activity for the oxygen evolution reaction, and 180° is the optimal value.^13^ The rare-earth orthoferrites have also attracted attention due to their interesting magnetic properties such as spin reorientation (*T*_SR_), antiferromagnetic transition of Fe (*T*_N1_), compensation effect (*T*_comp_), ordering of RE moments (*T*_N2_), and weak ferromagnetism (canted antiferromagnetism).^14^ These properties also depend on the degree of structural distortion.

In this work, we have investigated the use of mild hydrothermal conditions for the direct crystallization of RE-substituted lanthanum ferrites (La_1–*x*_RE_*x*_)FeO_3_. This followed from the work by Feng and coworkers who reported the hydrothermal crystallization of ternary REFeO_3_ perovskites, aided by the inclusion of urea in the reagent solutions.^15^ Hydrothermal synthesis has more generally been applied for the synthesis of a wide variety of perovskite oxides^16^ and various iron oxide materials^17^ and offers the advantage of the control of the crystal form and, in some cases, the formation of metastable polymorphs. Herein, we explore the limitations of forming solid solutions of the ferrite perovskites and have investigated the magnetic properties of the resulting materials as a signature of structure distortion and phase separation. The evolution of the crystal morphology with A-site substitution is also considered, which is related to phase separation when a large size mismatch of A-site cations is used.

## Experimental Section

### Materials and Synthesis

La(NO_3_)_3_·6H_2_O (99.9%), Nd(NO_3_)_3_·6H_2_O (99.9%), Gd(NO_3_)_3_·6H_2_O (99.9%), Yb(NO_3_)_3_·6H_2_O (99.9%), Sm(NO_3_)_3_·6H_2_O (99.9%), Ho(NO_3_)_3_·6H_2_O (99.9%), and Er(NO_3_)_3_·6H_2_O (99.9%) were obtained from Alfa Aesar, while Fe(NO_3_)_3_·9H_2_O (98%) and urea (NH_2_CONH_2_) (99–100.5%) were purchased from Sigma Aldrich. KOH (85%, pellets) was purchased from Fisher Scientific. The synthesis of La_*x*_RE_1–*x*_FeO_3_ (RE = Nd, Sm, Gd, Ho, Er, Yb, and Y) was undertaken by placing 5 mL of 0.4 M Fe(NO_3_)_3_ in a 40 mL Teflon-lined steel autoclave, to which was added the required stoichiometric amounts of 0.4 M RE(NO_3_)_3_ and 0.4 M La(NO_3_)_3_. After this, 4.5 g of KOH was added to the mixture (∼60% fill) and homogenized by stirring for 30 min before being allowed to cool to room temperature. Finally, 1.5 g of urea was added with further stirring and the reactant solution was sealed inside the autoclave and heated for 48 h at 240 °C. After cooling to room temperature, the as-produced powders were washed using deionized water before being dried in air at 80 °C. The washed and dried materials were then gently ground using a pestle and mortar before further study.

### Characterization Methods

Powder X-ray diffraction data were collected in Bragg–Brentano geometry with a Panalytical Empyrean diffractometer with Co Kα1 and Kα2 radiation (average wavelength = 1.7889 Å) and a PIXcel solid-state detector. Cobalt radiation was chosen to eliminate the high background of X-ray fluorescence that occurs when iron is exposed to Cu Kα. The data were collected over a range of 2θ, 20°–90°, using a step size of ∼0.013°, with sample spinning at 15 rpm and a measurement time 1 h scans. High-resolution XRPD data for structure refinement were obtained on Beamline I11 (wavelength = 0.82456 Å) at the Diamond Light Source Ltd., U.K. Samples were loaded into 0.1 mm internal diameter borosilicate capillaries and measured at room temperature in transmission geometry using a position-sensitive Mythen detector. Electron microscopy was undertaken on Zeiss Gemini SEM 500 using an Inlens detector with an accelerating voltage of 3 kV and a 20 μm aperture. Elemental analysis was performed on the electron microscope with an Oxford Instruments X-MaxN 150 solid-state detector with an accelerating voltage of 10 kV, a 30 μm aperture, and with the high current setting enabled. Spot analysis was undertaken a minimum of 5 times on a given sample and the results were averaged. Acquisition and control were handled by Oxford Instruments AZtecEnergy software. The zero-field cooled (ZFC) and field-cooled cooling (FCC) magnetization measurements were performed in an applied field of 1 kOe using a Quantum Design MPMS-5S and an MPMS-3 SQUID magnetometer. Raman spectra were recorded with a Renishaw inVia Reflex Raman microscope with a low wave number spectral cutoff at about 120 cm^–1^. Experiments were conducted in micro-Raman mode at room temperature by using a 633 nm He-Ne laser as an exciting wavelength. It is well known that Raman spectra recorded on transition-metal oxides often show strong dependence on the exciting laser power, leading to structural modifications, phase transitions, or even locally decomposed material. In order to avoid this situation, the experiments were performed with a laser power of <1 mW under the microscope to avoid structural transformations or overheating from taking place.

## Results

### La_*x*_Nd_1–*x*_FeO_3_ Solid Solutions

**Figure 1:**
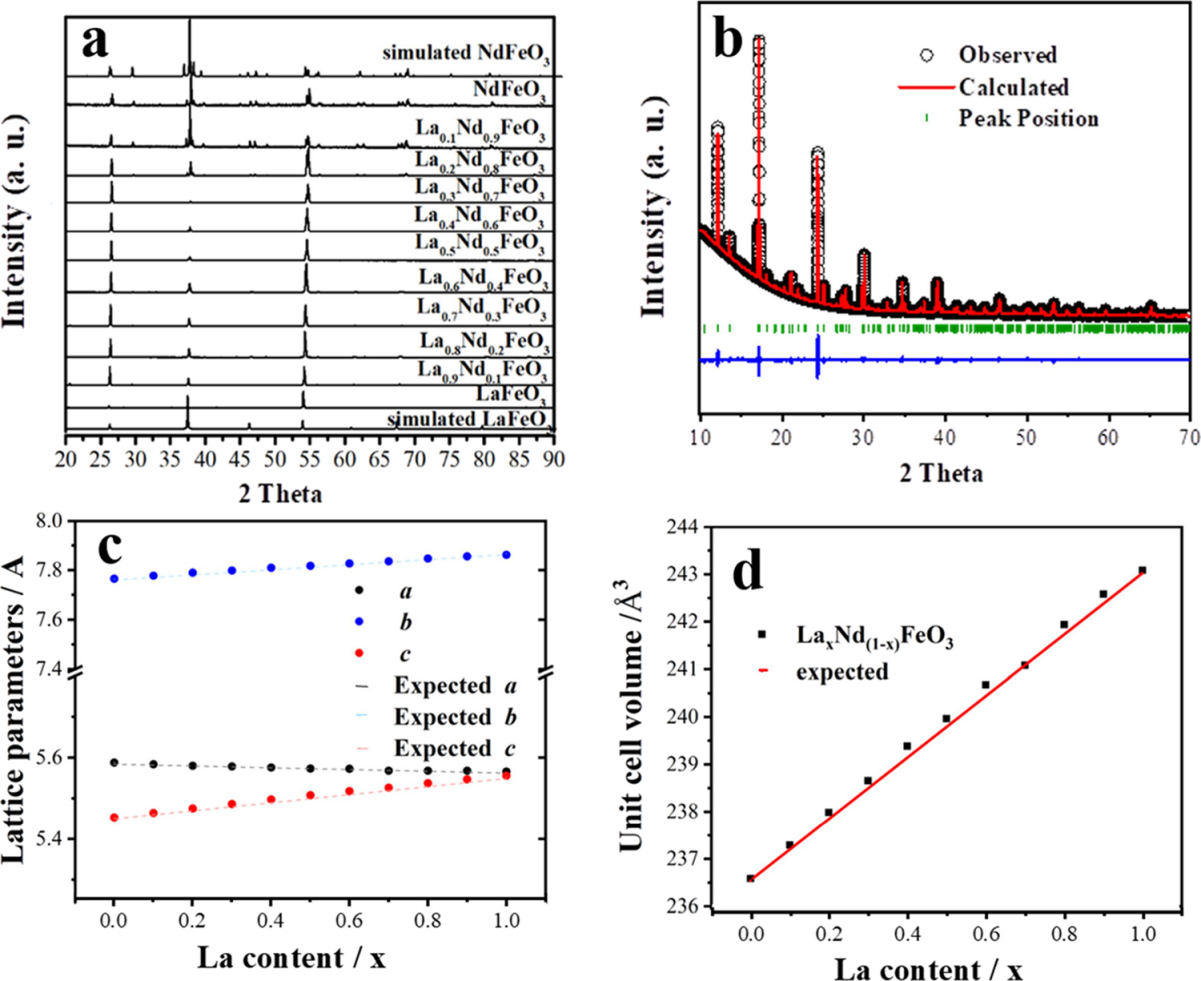
(a) Comparison of PXRD (λ = 1.7889 Å) patterns of simulated LaFeO_3_,^19^ NdFeO_3_, and synthesized La_*x*_Nd_1–*x*_FeO_3_; (b) Rietveld fit for synchrotron XRD (λ = 0.82456 Å) pattern of La_0.5_Nd_0.5_FeO_3_, (c) refined La_*x*_Nd_1–*x*_FeO_3_ parameters, (d) unit cell volume of La_*x*_Nd_1–*x*_FeO_3_ (the error bars are smaller than the data points). Expected lines are extrapolated from the lattice parameters of the end members.

To our knowledge, this is the first report of this perovskite solid solution. As shown in Figure 1a, the as-prepared La_*x*_Nd_1–*x*_FeO_3_ samples have similar X-ray diffraction patterns, which can be assigned to an orthorhombic perovskite structure, although the effects of the preferred orientation can be seen in relative peak intensities from these laboratory data. An obvious shift in the Bragg peak position occurs because of the smaller ionic radius of Nd^3+^ (1.27 Å) compared to La^3+^ (1.36 Å).^18^ The space group *Pnma* was used in structure refinement (it should be noted that some literature reports use the nonstandard setting *Pbnm* of the same space group for this distortion of the perovskite). Figure 1b shows the Rietveld fit obtained for one composition (see the Supporting Information, Figure S5 and Table S1 for fitted patterns, refined parameters and fitting statistics of all materials). As Figure 1c and d depict, the substitution of the smaller ion Nd^3+^ induces changes in the cell parameters, the value of *a* and *c* become increasingly different, and the cell volume linearly decreases with increased Nd^3+^. The refined lattice parameters of the end members, LaFeO_3_ and NdFeO_3_, agree well with those reported in the literature for materials of the same composition prepared by conventional synthetic methods (Table S1).

**Figure 2:**
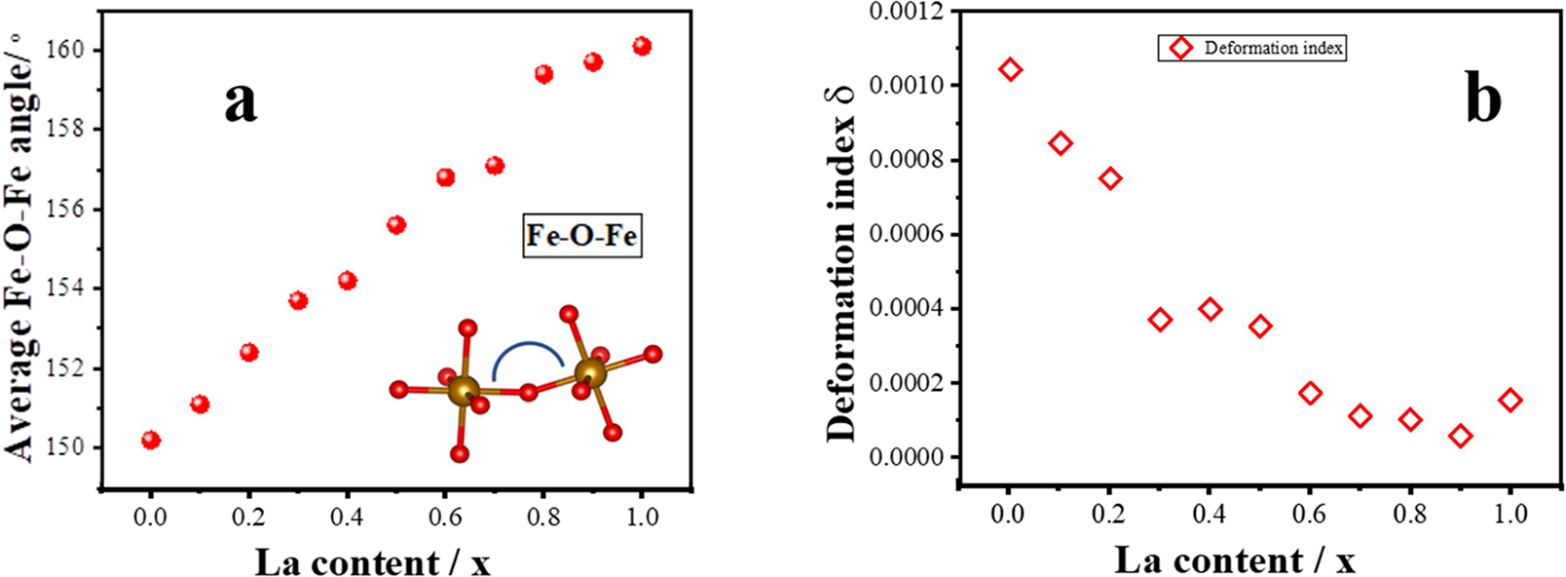
(a) Average Fe–O–Fe angle ⟨Fe–O–Fe⟩ and (b) internal deformation index δ of La_*x*_Nd_1–*x*_FeO_3_, which is an indicator of the FeO_6_ octahedral tilting degree. Error bars are smaller than the size of the data points.

For an evaluation of the distortion degree of FeO_6_ octahedra in La_*x*_Nd_1–*x*_FeO_3_, two variables were employed, *viz*, the deformation index δ and the average Fe–O–Fe angle ⟨Fe–O–Fe⟩. Specifically, δ is used to describe the deformation of FeO_6_ from regular octahedra and is calculated by
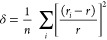
1where *r_i_*, *r*, and *n* represent the Fe–O bond length, average Fe–O bond length, and the number of Fe–O bonds in an FeO_6_ octahedron, respectively.^12^ The other variable ⟨Fe–O–Fe⟩, the mean value of Fe–O–Fe bond angles, is used to indicate the tilting degree of FeO_6_ octahedra. When the FeO_6_ octahedra are more tilted, the ⟨Fe–O–Fe⟩ angle is smaller. The average Fe–O–Fe angle and the distortion degree of FeO_6_ are found to increase with the enhancement of the Nd^3+^ amount in samples, as shown in Figure 2.

**Figure 3:**
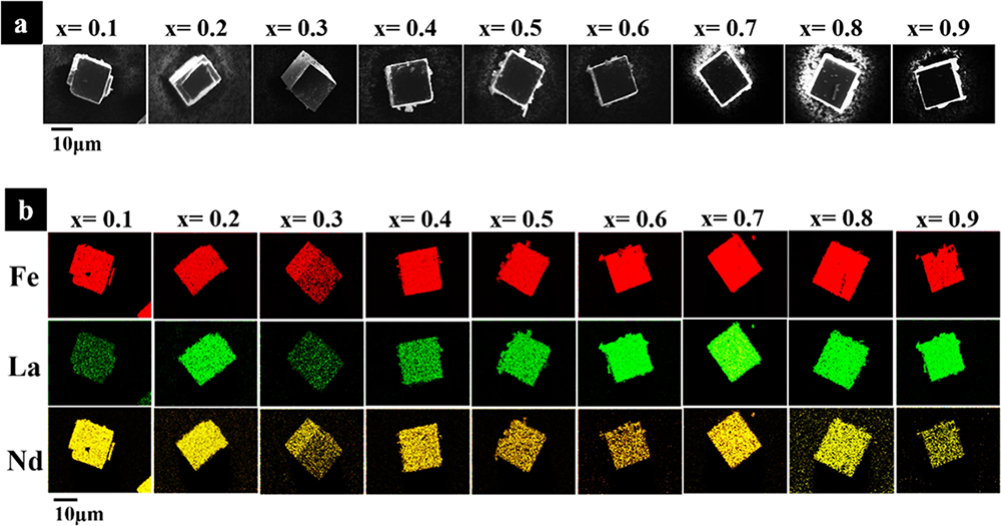
(a) SEM Electron micrographs and (b) EDS maps of La_*x*_Nd_1–*x*_FeO_3_ (*x* = 0.1, 0.2,...,0.9).

Scanning electron microscopy (SEM) with elemental analysis by EDS of the La_*x*_Nd_1–*x*_FeO_3_ samples (*x* = 0.1, 0.2,...,0.9), Figure 3, shows that the samples present cubic-shaped crystallites with some surface faceting evident for some samples, and with La, Fe, and Nd distributed uniformly in each particle. This is in good agreement with the XRD analysis that homogeneous solid solutions are formed. Quantification of the EDS results shows the expected trend in atomic composition across the solid solution (Table S10).

**Figure 4:**
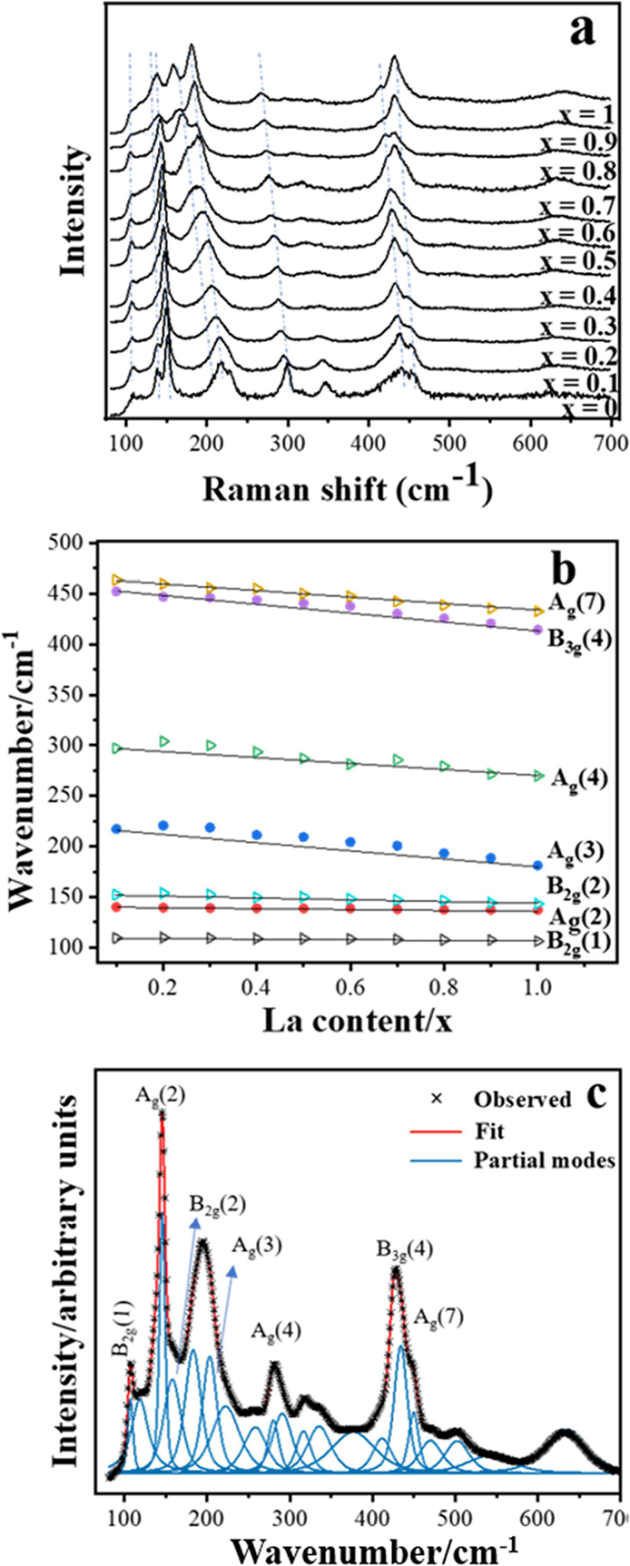
(a) Raman spectra of La_*x*_Nd_1–*x*_FeO_3_ with the assignment of major bands, (b) wavenumber shifts in phonon modes for La_*x*_Nd_1–*x*_FeO_3_ series, and (c) fitted Raman spectra of La_0.5_Nd_0.5_FeO_3_.

The Raman spectra (Figure 4a) of all La_*x*_Nd_1–*x*_FeO_3_ samples exhibit typical features of an orthorhombic perovskite. The band positions have an obvious evolution with composition with a continuous shift to lower frequencies when the La content increases, consistent with the formation of a homogeneous solid solution. The observed phonon modes were assigned to specific vibrational symmetries using data from a previous systematic study of REFeO_3_ that included the end members LaFeO_3_ and REFeO_3_.^20,21^ The prior knowledge of mode assignments for the end members and the continuous change between the spectra of each solid solution aided the assignment of observed bands for this unreported series. The phonon positions and line widths were determined, and each spectrum was modeled with 15 or 16 modes (Supporting Information S12). For most of the samples, it is possible to assign at least 6 modes with their corresponding vibrational symmetries, but some modes remained un-assigned because of the overlapping of the modes, and some modes are not assigned so far in the literature. The assigned phonon modes and their positions of NdFeO_3_ are listed in Table 1. Blue shifts of O–Fe–O bending (∼441 cm^–1^) modes were observed in all La_*x*_Nd_1–*x*_FeO_3_, in comparison to LaFeO_3_ and NdFeO_3_, indicating the distortion of the FeO_6_ octahedra (Figure 4b).

**Table 1:** Assigned Phonon Modes and Atomic Motions of NdFeO_3_

symmetry	position/cm^–1^	atomic motion
*A*_g_(2)	140	*A*(*z*) out-of-phase
*A*_g_(3)	217	FeO_6_ rotation, in-phase
*A*_g_(4)	297	O(1) *x*–*z* plane
*B*_3g_(4)	452.3	FeO_6_ scissor-like bending, out-of-phase
*A*_g_(7)	463.5	FeO_6_ scissor-like bending
*B*_2g_(1)	110	*A*(*z*), in-phase in *x*–*z*, out-of-phase in *y*
*B*_2g_(2)	152.1	*A*(*x*), out-of-phase

**Figure 5:**
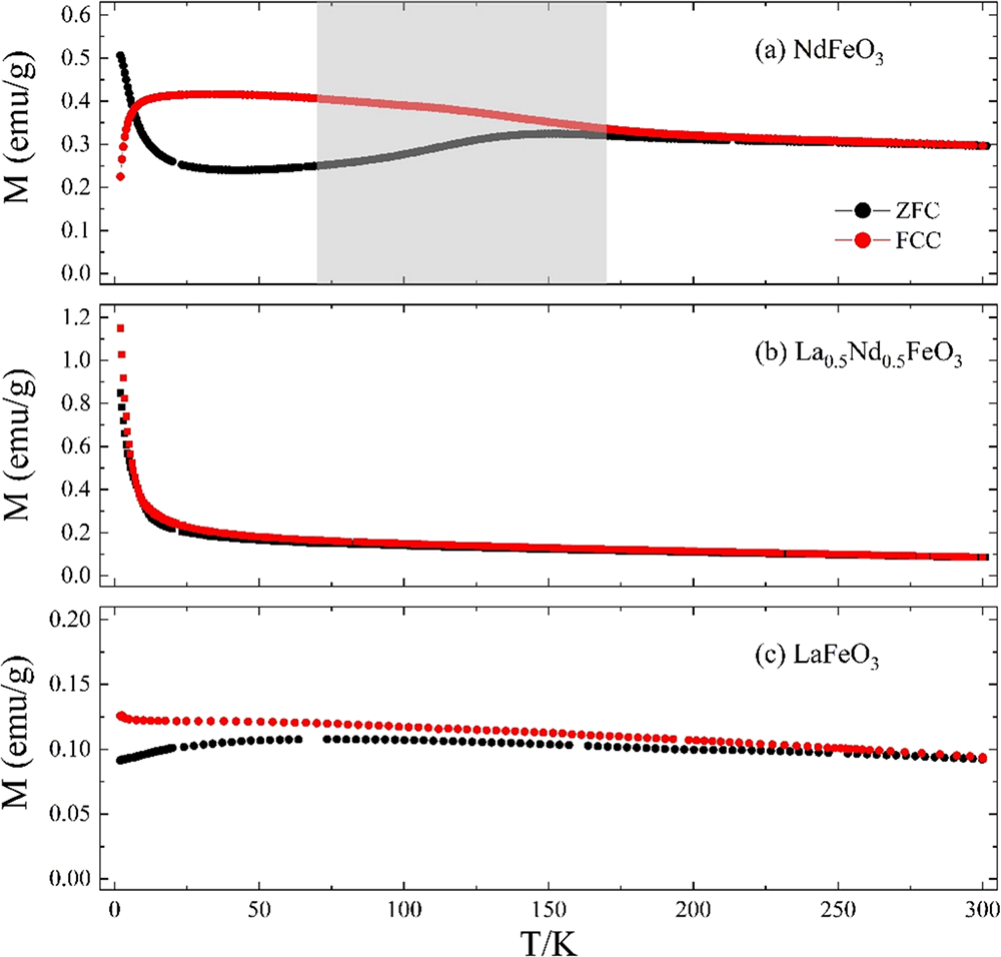
Temperature dependence of the magnetization (ZFC and FCC) of NdFeO_3_, La_0.5_Nd_0.5_FeO_3_, and LaFeO_3_ in 1 kOe from 2 to 300 K. The gray shaded area indicates the temperature region over which the spin reorientation takes place.

Magnetization versus temperature, *M*(*T*), measurements for pure NdFeO_3_ are shown in Figure 5a. The data are consistent with a strong Fe–Fe super-exchange interaction that leads to long-range antiferromagnetic (AFM) ordering within the Fe sublattice well above 300 K. Previous neutron diffraction studies showed a G_*x*_-type order below a Néel temperature *T*_N_ ≈ 690 K, with the Fe moments aligned antiparallel to their 6 nearest neighbors along the *x*-axis (*a*-axis).^21−23^ The Nd–Fe interactions are of intermediate strength and result in a polarization of the Nd^3+^ ion moments. A spin reorientation transition is reported from both neutron diffraction and ac and dc magnetization studies with the Gz-type order below *T*_SR1_.^21−26^ Here, we find a spin reorientation indicated by the shaded region between *T*_SR1_–*T*_SR2_: 70–170 K. A similar behavior is seen in both single crystal NdFeO_3_ (*T*_SR1_–*T*_SR2_: 100–190 K) and polycrystalline NdFeO_3_ produced by a hydrothermal method (*T*_SR1_–*T*_SR2_: 70–150 K).^22,25^ Nd polarization and spin reorientation lead to considerable field history dependence between the ZFC and FCC curves. The relative alignment of the Nd and Fe moments can lead to a compensation point as high as *T*_comp_ = 7.6 K.^25,27^ Here, there is a downturn in the FCC *M*(*T*) at low temperatures, but the strength of the measuring field means *T*_comp_ is suppressed to below 2 K. The weak Nd–Nd interactions lead to a rapid change in both the ZFC and FCC magnetization at the lowest temperatures, which is a precursor to long-range AFM ordering of the Nd^3+^ magnetic moments at 1.5 K.^22^

The magnetic response of LaFeO_3_, shown in Figure 5c, is different from NdFeO_3_ because the A-site is diamagnetic. The magnetic properties of LaFeO_3_ originate solely from interactions within the Fe sublattice, with long-range G_*x*_-type AFM ordering below 740 K, but with a small canting of the moments away from the *a* axis.^21,23,24,28−30^ There is no spin reorientation phenomenon.^21,29^ The magnitude of magnetization is similar to previous work in which polycrystalline LaFeO_3_ was also synthesized by the wet-chemical method.^31^

In La_0.5_Nd_0.5_FeO_3_, (see Figure 5b) the magnetization at 300 K is reduced compared to NdFeO_3_ because the number of Nd^3+^ ions is halved. At lower temperatures, the paramagnetic contribution of the Nd^3+^ ions becomes more prominent and there is almost no hysteresis between the ZFC and FCC curves and no clear evidence for a spontaneous spin reorientation. This can be attributed to the presence of an atomically homogenous solid solution. The diamagnetic La^3+^ is dispersed into the NdFeO_3_ structure and disrupts the Nd–Fe intermediate magnetic structures present in the NdFeO_3_ phase, disturbing the magnetic anisotropy of the Nd sublattice, which is considered as a key factor for a spin-reorientation.^32−36^ Taken together, the measured diffraction, spectroscopic, and magnetization data of La_*x*_Nd_1–*x*_FeO_3_ prepared by hydrothermal chemistry provide clear evidence for homogeneous solid solutions. A similar analysis of La_*x*_Sm_1–*x*_FeO_3_ and La_*x*_Gd_1–*x*_FeO_3_ shows that these samples are also homogeneous solid solutions (see Supporting Information Figures S1, S2, S6, S7, S13–S18, S23, S24 and Tables S2, S3, S8, S9).

### La_*x*_Er_1–*x*_FeO_3_ Materials

**Figure 6:**
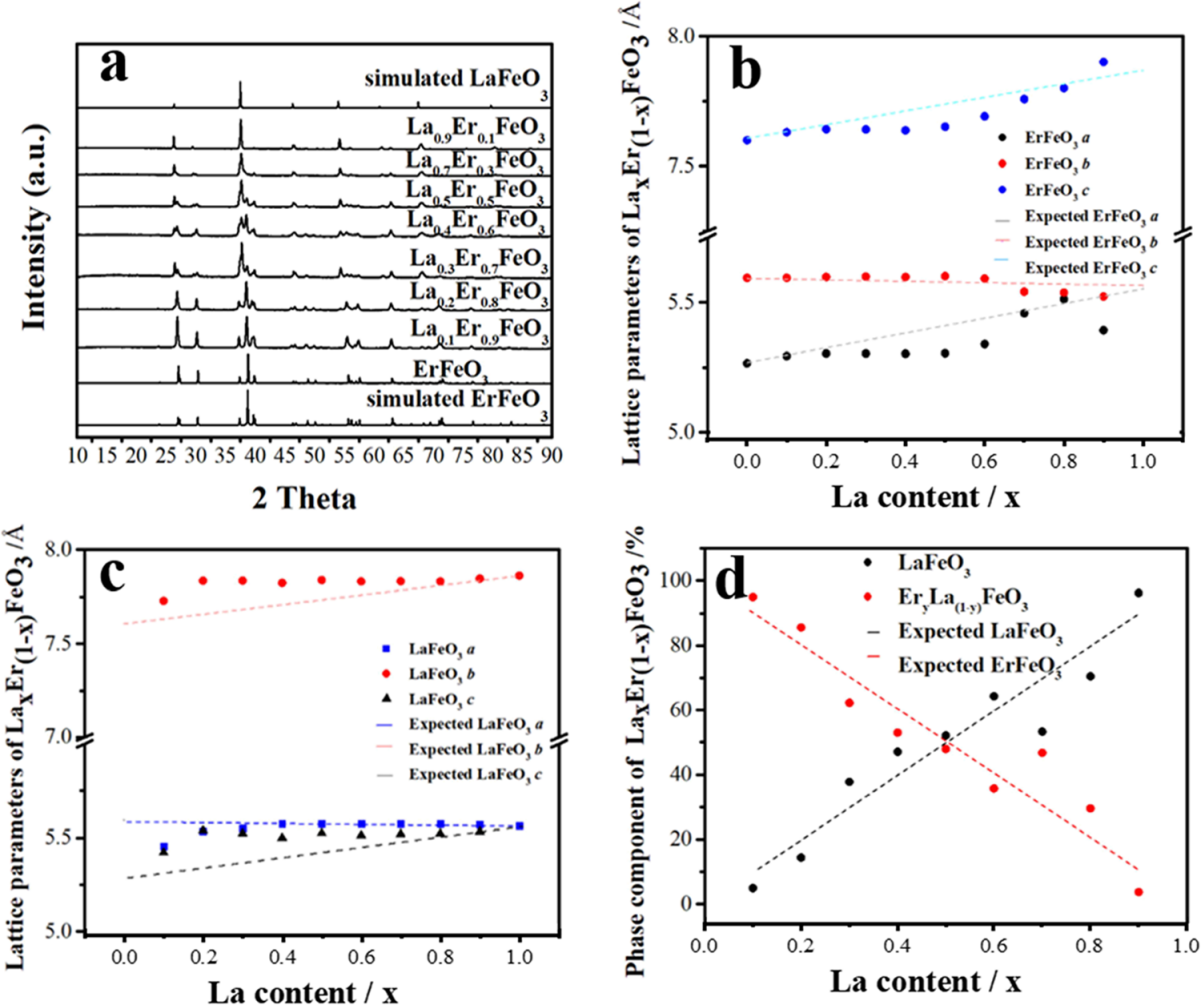
(a) Comparison of PXRD (λ = 1.7889 Å) patterns of simulated LaFeO_3_,^19^ ErFeO_3_,^25^ and synthesized La_*x*_Er_1–*x*_FeO_3_; (b) refined ErFeO_3_ parameters, (c) refined LaFeO_3_ parameters, (d) refined phase component of La_*x*_Er_1–*x*_FeO_3_.

Powder XRD patterns shown in Figure 6 reveal that all La_*x*_Er_1–*x*_FeO_3_ materials crystallize as two different perovskite phases, which can initially be assigned as LaFeO_3_ and ErFeO_3_. This is confirmed by the evolution of three ErFeO_3_ peaks (020, 112, 021) between 36° and 41° 2θ and the LaFeO_3_ peak [002] at 37.6° 2θ. However, a closer inspection shows that with increasing amount of La^3+^ in the samples, the ErFeO_3_ Bragg peaks shift slightly to lower angles, indicating lattice expansion, while LaFeO_3_ peaks remain in the same positions. Thus, we conclude that the La_*x*_Er_1–*x*_FeO_3_ samples are composed of LaFeO_3_ and La^3+^-substituted ErFeO_3_ (Er_1–*y*_La_*y*_FeO_3_) and Rietveld analysis was undertaken using LaFeO_3_ and ErFeO_3_ (with the *Pbnm* space group) to quantify this (Supporting Information Figure S9 and Table S5). The refinement results of the structural parameters are summarized in Figure 6 where it can be confirmed that the cell parameters of LaFeO_3_ change only by a small amount while the cell parameters of ErFeO_3_ show changes with the addition of La. This suggests that, although there are two distinct phases, some La^3+^ ions enter into the ErFeO_3_ structure.

**Figure 7:**
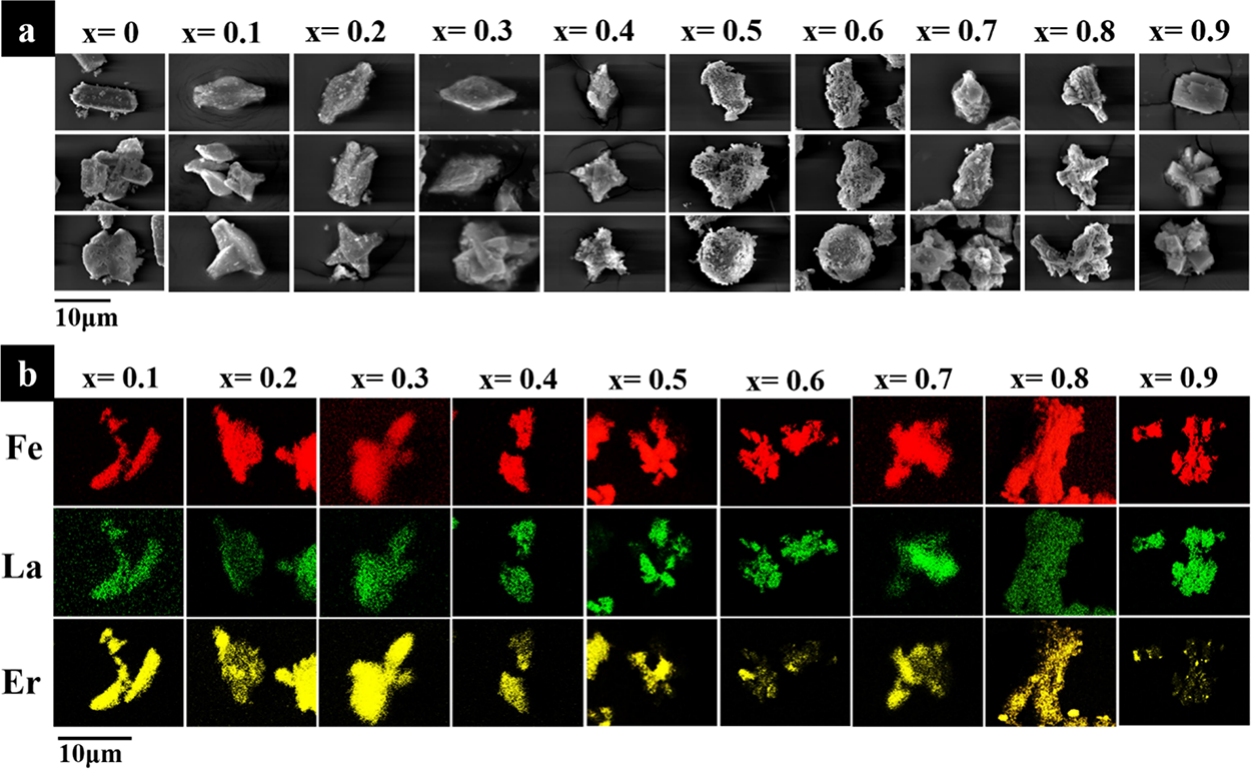
(a) Electron micrographs and (b) EDS maps of La_*x*_Er_1–*x*_FeO_3_ (*x* = 0,0.1,0.2,..., 0.9).

The morphologies and EDS maps of the La_*x*_Er_1–*x*_FeO_3_ samples are shown in Figure 7. All the samples have relatively small particle sizes, which is estimated in the range of 10–25 μm. The crystal morphology is much less distinct than the well-formed cube-shaped crystallites seen for La_*x*_Nd_1–*x*_FeO_3_, and even for La_0.9_Er_0.1_FeO_3_, the crystal habit is modified to a lozenge shape. With an increased amount of Er^3+^, substantial changes in crystal morphology are seen and it can be noted that the samples are constructed as agglomerates of small bricks of crystals.

The EDS maps, Figure 7b, show that the dispersion of La and Er is not homogeneous in the samples. Taking the La_0.5_Er_0.5_FeO_3_ sample as an example, while Fe is found in all regions of the sample, there are regions that contain only Er, while others contain only La. In other areas, all three elements are present, at least on the micron length scale. The information that is deduced from EDS maps is in good agreement with XRD refinement that La_*x*_Er_1–*x*_FeO_3_ samples were composed of pure LaFeO_3_ phase and La^3+^ doped ErFeO_3_.

**Figure 8:**
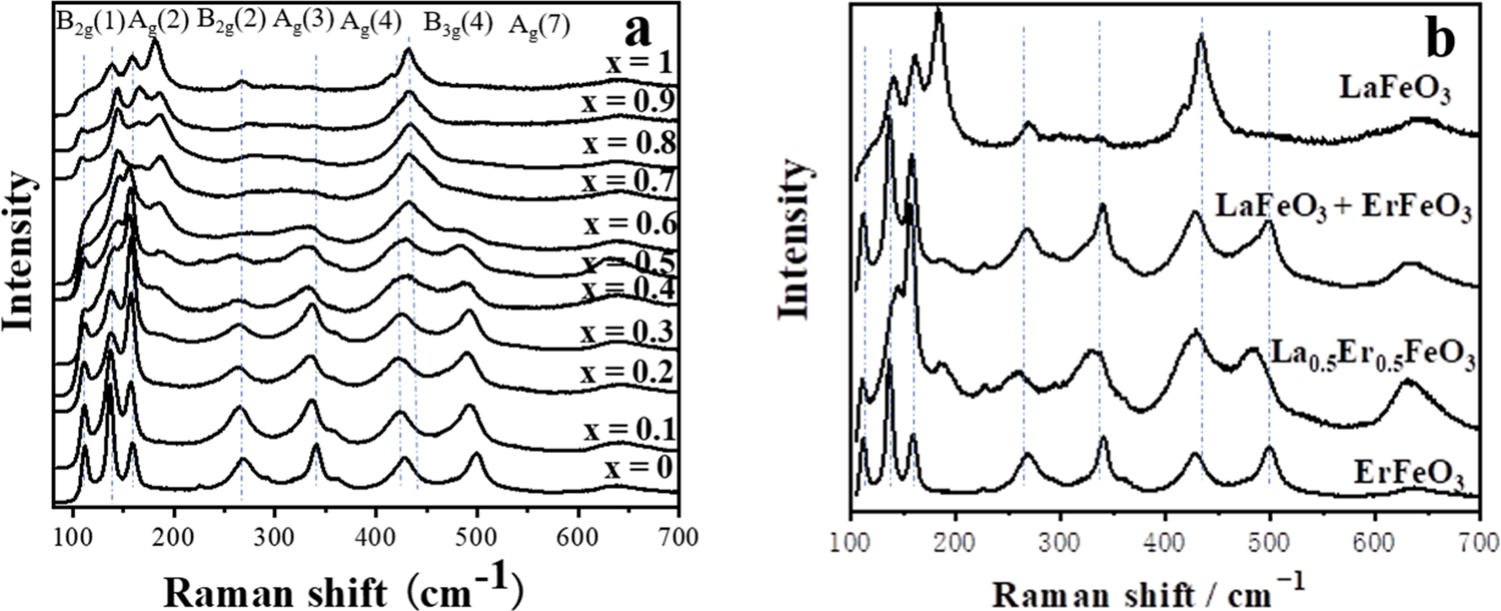
(a) Raman spectra of La_*x*_Er_1–*x*_FeO_3_, (b) Raman spectra of ErFeO_3_, La_0.5_Er_0.5_FeO_3_, and LaFeO_3_ compared to the simulated spectra of 0.5ErFeO_3_ + 0.5LaFeO_3_ (weighted sum of the measured spectra of the end members).

**Figure 9:**
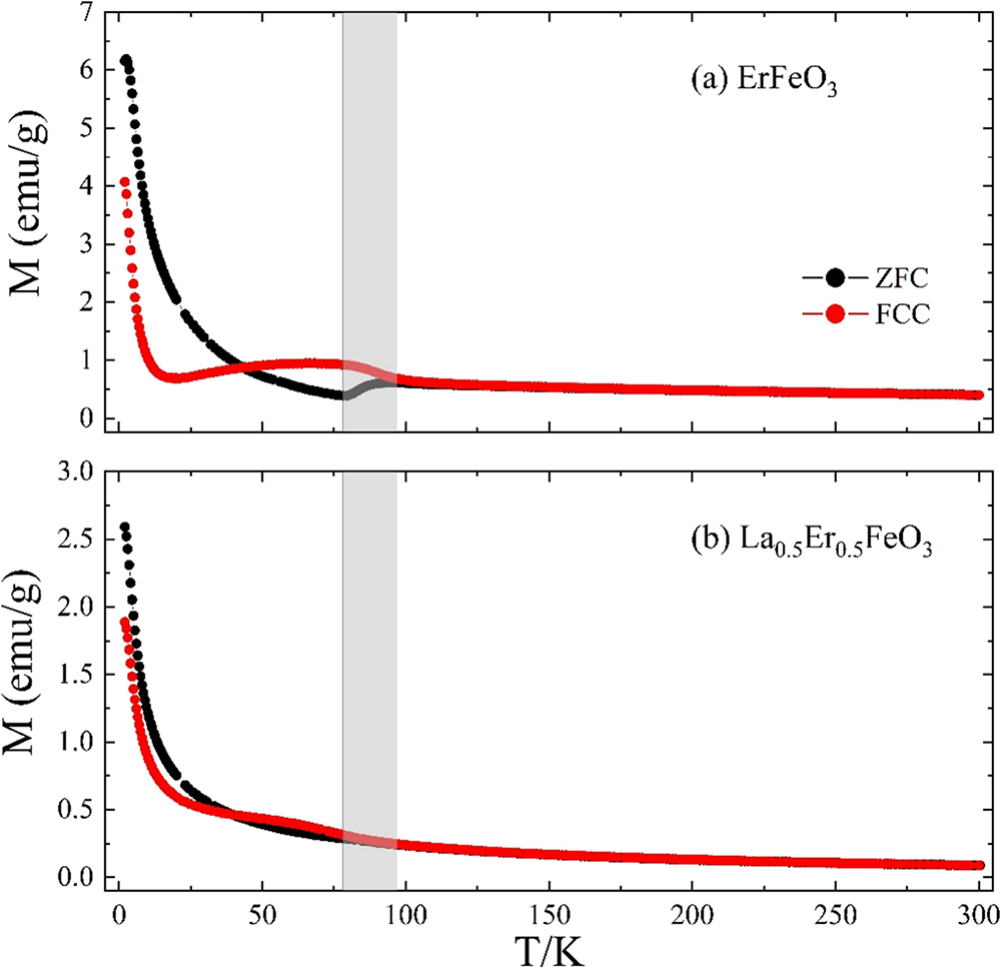
Temperature dependence of the magnetization (ZFC and FCC) of ErFeO_3_ and La_0.5_Er_0.5_FeO_3_ in 1 kOe from 2 to 300 K. The gray shaded area indicates the temperature region over which the spin reorientation takes place.

Figure 8a shows the Raman spectra of La_*x*_Er_1–*x*_O_3_. Unlike the solid solutions discussed above, the spectral signatures do not show a gradual shifting in band positions as the La content changes toward LaFeO_3_. As Figure 9b shows, instead, the spectra can be interpreted as a superposition of two distinct signatures, but with broadening compared to the pure phases. The broadening of the modes provides some evidence for the presence of mixed phases formed for a limited substitutional range.

Magnetization versus temperature measurements for pure ErFeO_3_ and La_0.5_Er_0.5_FeO_3_ powders are shown in Figure 9. ErFeO_3_ undergoes a transition below 640 K to G-type antiferromagnetism within the Fe sublattice.^21,23,24,26^ Studies on single crystals show that the Fe moments are directed along the *a*-axis with a small canting, leading to a net moment along *c*. As the temperature is lowered, the Er^3+^ RE ions are increasingly polarized antiparallel to the Fe moments, and their effective anisotropy increases. ErFeO_3_ undergoes a spin reorientation transition below *T*_SR2_ with the Fe spins rotating in the *ac* plane to lie almost parallel to the *c*-axis by *T*_SR1_ with a net Fe moment now along the *a*-axis.^25,37,38^ In this work, (see Figure 9a) a spontaneous spin reorientation was observed in the ErFeO_3_ material over the temperature range *T*_SR1_–*T*_SR2_, 78 to 97 K, which leads to field history dependence. Similar behavior was observed for ErFeO_3_ powders synthesized via a sol–gel combustion method.^39^ Previous work has shown further cooling leads to a spin compensation at *T*_comp_ = 38–45 K. with the net Fe and Er moments canceling.^21,25^ This compensation point is close to the crossover temperature of the ZFC and FCC curves. Lower field data (see Supporting Information Figure S21) shows a compensation at ∼40 K in the ErFeO_3_. At low temperatures, an upturn in *M*(*T*) reflects the increasing contribution from the Er moments. A peak in the ZFC curve at 2.5 K marks the onset of long-range AFM order between the Er moments, slightly lower than the 3.7–4.5 K reported previously.^25,38^

Figure 9b shows the *M*(*T*) data for the La_0.5_Er_0.5_FeO_3_ powders. The temperature dependence and magnitude of the signal at 300 K suggest that in common with La and Er orthoferrite, the Fe moments order antiferromagnetically at some much higher temperature. Although the degree of hysteresis is reduced in La_0.5_Er_0.5_FeO_3_, a spin reorientation is clearly still present at a similar temperature. At low temperatures, the total magnetization of the La_0.5_Er_0.5_FeO_3_ powders is close to half the magnetization of ErFeO_3_. The overall magnetic response is consistent with a phase separated mixture of ErFeO_3_ diluted by LaFeO_3_, albeit with some incorporation of a small number of nonmagnetic La ions into the ErFeO_3_ which disrupt the Er–Fe exchange interactions. This scenario is in good agreement with the XRD and EDS results.

### La_*x*_Y_1–*x*_FeO_3_

**Figure 10:**
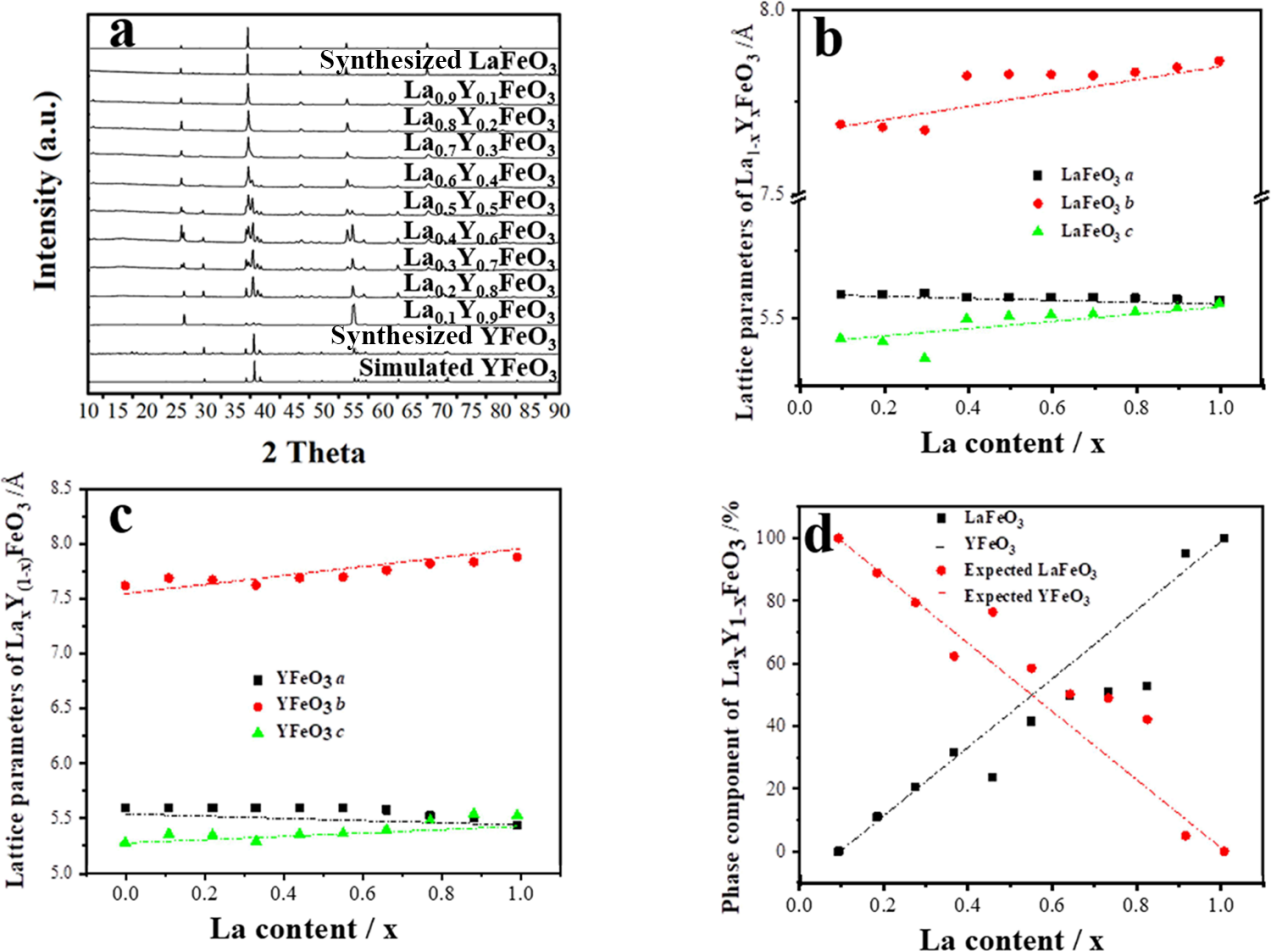
(a) Comparison of PXRD (λ = 1.7889 Å) patterns of simulated LaFeO_3_,^19^ YFeO_3_,^40^ synthesized LaFeO_3_, YFeO_3_, and La_*x*_Y_1–*x*_FeO_3_; (b) refined LaFeO_3_ parameters, (c) refined YFeO_3_ parameters, (d) refined phase component of La_*x*_Y_1–*x*_FeO_3_.

The comparison in Figure 10a between the measured patterns and the simulated patterns of both YFeO_3_ and LaFeO_3_ clearly indicate the samples contain two distinct crystallographic phases for 0.2 ≤ *x* ≤ 0.8. This is evident by the evolution of three strong YFeO_3_ peaks between 36° and 41° 2θ but the retention of the strong LaFeO_3_ at 37.4° 2θ as the yttrium content increases. These signature peaks begin to shift as the composition of the samples change, with YFeO_3_ peaks shifting to lower angles (expanding lattice) and LaFeO_3_ peaks shifting to higher angles (contracting lattice). This peak shift is consistent with a substitution of La into the YFeO_3_ lattice (expanding lattice, shifting Bragg peaks to lower 2θ positions) and Y into the LaFeO_3_ lattice (contracting lattice, shifting Bragg peaks to higher 2θ positions). This suggests that, while there are two distinct phases, they are both taking up the other A-site metal to a limited extent.

**Figure 11:**
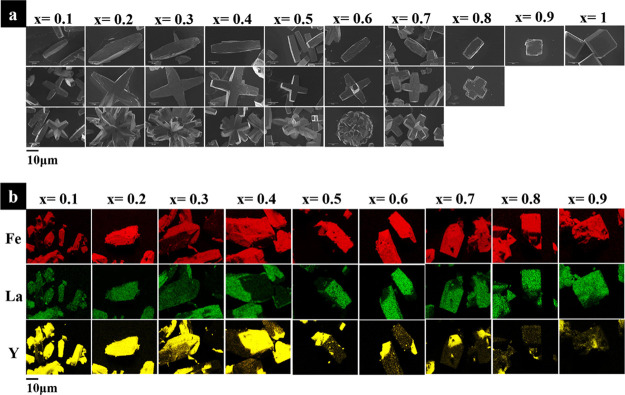
(a) Electron micrographs detailing the evolution in La_*x*_Y_1–*x*_FeO_3_ crystal morphology, and (b) EDS maps of fractured crystals.

Electron microscopy of the materials reveals substantial changes in crystal morphology with the increase of yttrium used in the precursor mixture. Figure 11a charts this morphological evolution as a function of Y% and evident is an elongation of the original cube shape seen in pure LaFeO_3_ system to a maximum length of ∼25 μm up from a core particle size of ∼10–15 μm, followed by additional perpendicular growths at the particle center. These additional growths appear to always occur in the same plane as the first axial elongation and reach the same length maximum with a full particle length in direction of primary and secondary axial growth equal at ∼25 μm. At higher concentrations of Y^3+^ the particles continue to grow arms from a central core, first in the “*z*” axis, i.e., in the direction perpendicular to the initial growth directions and then trending towards spheroids composed of multiple rods. Morphology changes are common in hydrothermal synthesis, with morphology changes reported for complexing/templating,^41,42^ agents or changes in pH,^43^ however, to our knowledge, there are no reported cases of a doped bi-phasic material undergoing crystal growth in a similar nature to that seen here.

Also evident is a mixture of Y and La within the crystals, determined by EDS, with EDS atomic % compositions detailed in Table S10. The EDS maps, Figure 11b, show clear evidence for intergrowths of the La-rich and Y-rich phases, and for the *x* = 0.5 material, it is apparent that at the crystal center of the “blades”, there is a notable concentration of Y, while at the tips of the blades, La is concentrated. This implies that the multipodal crystal morphology is created by the initial nucleation of YFeO_3_ and the subsequent growth of La-rich material.

**Figure 12:**
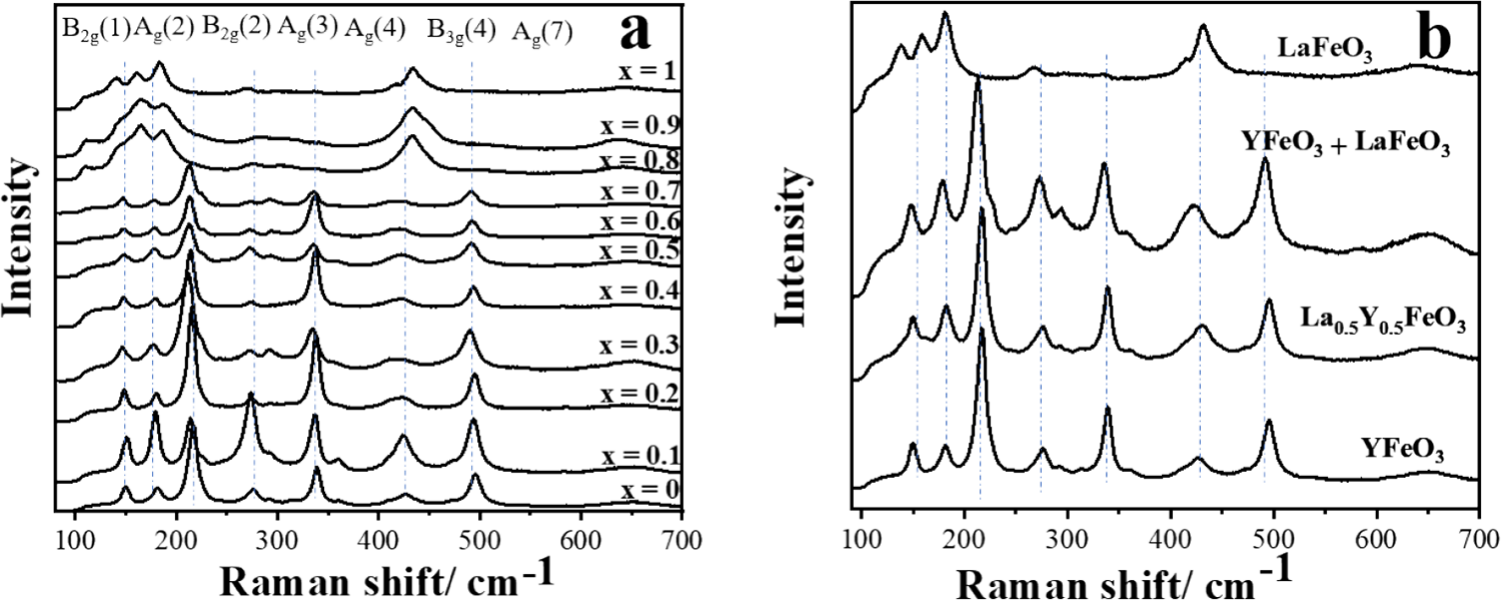
(a) Raman spectra of La_*x*_Y_1–*x*_FeO_3_, (b) Raman spectra of YFeO_3_, La_0.5_Y_0.5_FeO_3_, and LaFeO_3_ compared to the simulated spectra of 0.5YFeO_3_ + 0.5LaFeO_3_ (weighted sum of the measured spectra of the end members).

Figure 12a shows the Raman spectra of La_*x*_Y_1–*x*_O_3_. Unlike the solid solutions discussed above for La_*x*_Nd_1–*x*_O_3_, the spectral signatures do not show gradual shifting in band positions as the La content changes toward LaFeO_3_. As Figure 12b shows, instead, the spectra can be interpreted as a superposition of two distinct signatures, but with broadening compared to the pure phases. The broadening of the modes provides evidence for some degree of A-site mixing.

**Figure 13:**
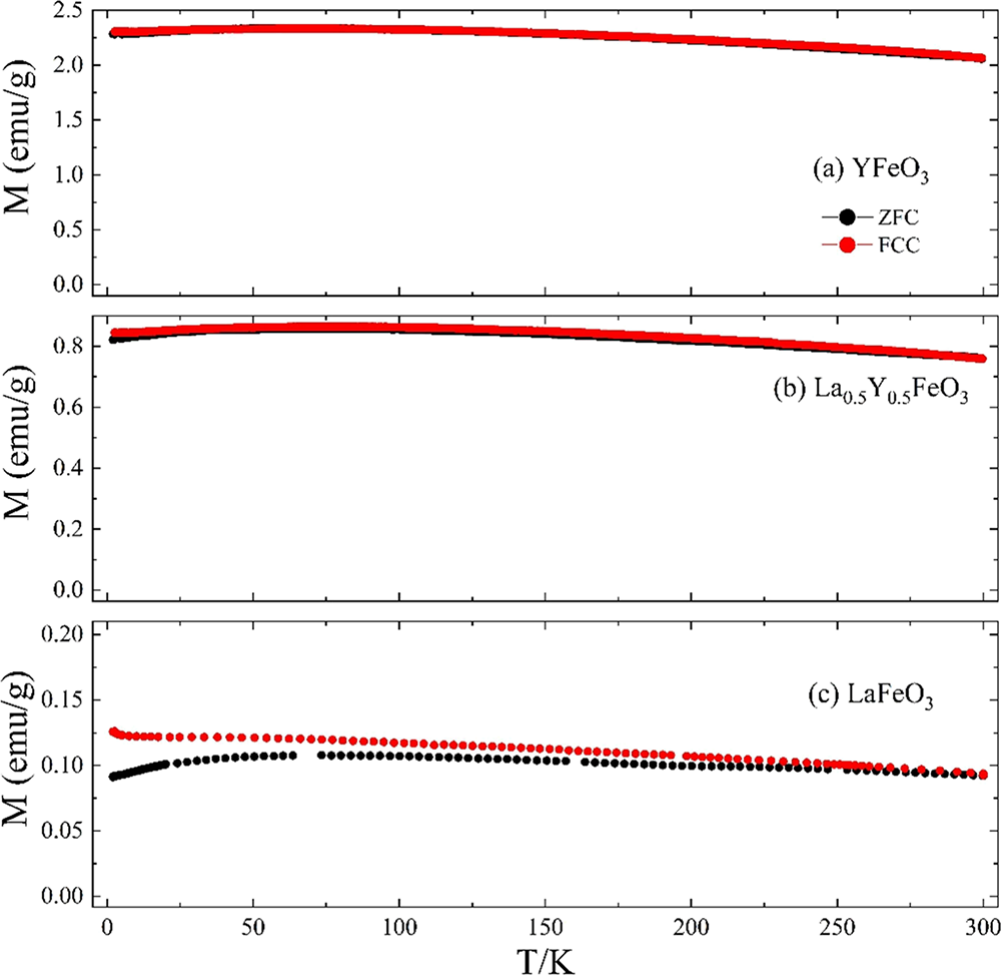
Temperature dependence of magnetization (ZFC and FCC) of YFeO_3_, La_0.5_Y_0.5_FeO_3_, and LaFeO_3_ in 1 kOe from 2 to 300 K.

Figure 13 shows the temperature dependence of the magnetization of YFeO_3_, La_0.5_Y_0.5_FeO_3_, and LaFeO_3_. Qualitatively, the magnetic responses of all three samples appear similar. Néel temperatures, *T*_N_ of YFeO_3_ (644.5 K) and LaFeO_3_ (740 K)^21,24,28^ are both well above the maximum temperature accessible in this work. Both the La^3+^ and Y^3+^ ions are diamagnetic and so the magnetism in La_1–*x*_Y_*x*_FeO_3_ series arises solely from the Fe sublattice. Canting of the Fe moments results in small net magnetization, giving rise to an almost temperature-independent weak ferromagnetic behavior with a hysteresis between the zero-field cooled and field-cooled cooling magnetization curves for all three materials.^30^ A very similar behavior was observed previously for YFeO_3_ nanoparticles.^44^ Distortions from an ideal perovskite structure are expected to be more significant in YFeO_3_ due to the smaller Y^3+^ ions, and this may lead to the larger signal observed for YFeO_3_.

La_*x*_Ho_1–*x*_FeO_3_ and La_*x*_Yb_1–*x*_FeO_3_ were studied as two more examples and the results from these phases also are consistent with phase-separation. (Supporting Information Figures S3, S4, S8, S10, S19, S20, S24, S26 and Tables S4 and S6).

## Discussion

Our results show the successful formation by a mild hydrothermal method of solid solutions of orthoferrites (La_1–*x*_RE_*x*_)FeO_3_ for RE = Nd, Sm, and Gd, as evidenced by a variety of experimental methods that probe the structural order over various length scales and supported by magnetization measurements. For Nd, this is the first report of this solid solution, while for Sm, examples have previously been prepared using a precursor decomposition route at 1000 °C,^45^ and for Gd only the phase La_0.9_Gd_0.1_FeO_3_ has been reported from the solid-state reaction at 1200 °C.^46^ For all of these materials, the difference in the ionic radius between the A-site substituents is smaller than 13.4%. In contrast, for RE = Ho, Er, Yb, and Y, the formation of homogeneous solid solutions is never successful, and there is only limited evidence for mixing of the two cations in a single phase. To our knowledge, there are no previous reports of the synthesis of these quaternary phases, using any synthesis method. For these materials, the difference in the cationic radii of the A-site substituent is larger than 17.2%. In the formation of solid solutions of perovskites, the Hume–Rothery law, well known for understanding the range of compositions of alloys, has been used to rationalize the compositions that can successfully be formed.^47−50^ From this work, it can be be suggested that when the radius disparity is more than 15% between the A-site cation and the intended substituent cation, segregation occurs, resulting in phase separation. In more recent computational work, the thermodynamics of the formability of perovskite solid solutions has been considered and one of the factors that is concluded to play a role in the successful formation of a homogeneous solid solution is the radius variance of A-site substituents.^51^

A significant new observation from our work is that despite the lack of formation of solid solutions, a modification of the crystal morphology is possible when two-phase mixtures are produced from hydrothermal crystallization. This is most evident for the largest disparity in the A-site cation radius (La and Y), where intergrowth structures are observed, implying that the one phase crystallizes first before the seeded growth of the second phase. This may imply a kinetic effect in the solution crystallization of RE ferrites, where different lanthanide cations lead to different rates of crystallization. This could also be the origin of why the formation of a continuous solid solution is not possible for cations of differing ionic radius, but instead, one phase is initially nucleated followed by the growth of the second in close proximity, leading to composite structures on the micron-scale.

## Conclusions

To study the effect of A-site substitution on the structure and properties of LaFeO_3_, a new series of samples have been synthesized by the hydrothermal method. A limit of solid-solution mixed-metal oxide formation from hydrothermal conditions has been discovered. It can be concluded that when doping different RE ions into the LaFeO_3_ structure, (1) if the radius between La^3+^ and the substituent ions is close, such as Nd^3+^, Sm^3+^, and Gd^3+^, homogeneous solid solutions will be formed; (2) when the radius difference is large, such as Ho^3+^, Er^3+^, and Yb^3+^, then instead of forming solid solutions, the substituent ion will cause samples to crystallize in separate phases, with a limited amount of element mixing in each. For the former situation, A-site substitution results in a significant change of the magnetic properties, and we have characterized some new solid solutions of ferrites as atomically homogeneous fine powders. The mixed-phase materials show intergrown crystallites with a distinct evolution of crystal morphology with chemical composition. Given the present interest in ferrite perovskites in various applications, ranging from multiferroics to heterogeneous catalysis, the ability to predict the crystallization of desired compositions and understand the limitations of a particular synthesis method is of utmost importance. Further work is needed to understand the atomic-scale mechanism for the complex intergrowth structures that we observe, such as using spectroscopic techniques, and this might also involve computational rationalization of the relationship between composition, crystal chemistry, and stability of solid solutions of perovskite oxides.

## Supporting Information Available

Full analysis of powder X-ray diffraction; Raman spectroscopy; scanning electron microscopy; and magnetization for all materials (PDF)
