# Supplementary material for: Limits of Solid Solution and Evolution of Crystal Morphology in (La1–xREx)FeO3 Perovskites by Low Temperature Hydrothermal Crystallization
Source: Inorg Chem. 2023 Mar 6;62(11):4503–13. doi: 10.1021/acs.inorgchem.2c04325 (PMC10031561; doi:10.1021/acs.inorgchem.2c04325)
Supplement: Supplementary file 1 — ic2c04325_si_001.pdf [file ic2c04325_si_001.pdf]

SUPPORTING INFORMATION

Limits of Solid Solution and Evolution of Crystal Morphology in $(La_{1-x}RE_x)FeO_3$ Perovskites by Low Temperature Hydrothermal Crystallization

Lu Jia[1,2], Matthew D. Lloyd[1], Martin R. Lees[3], Limin Huang[2], Richard. I. Walton[1*]

1. Department of Chemistry, University of Warwick, Coventry, CV4 7AL, U.K. *email r.i.walton@warwick.ac.uk

2. Department of Chemistry, Southern University of Science and Technology, Shenzhen, 518055, P. R. China

3. Department of Physics, University of Warwick, Coventry, CV4 7AL, U.K.

S1: Rietveld analysis results

1. $La_xSm_{1-x}FeO_3$:

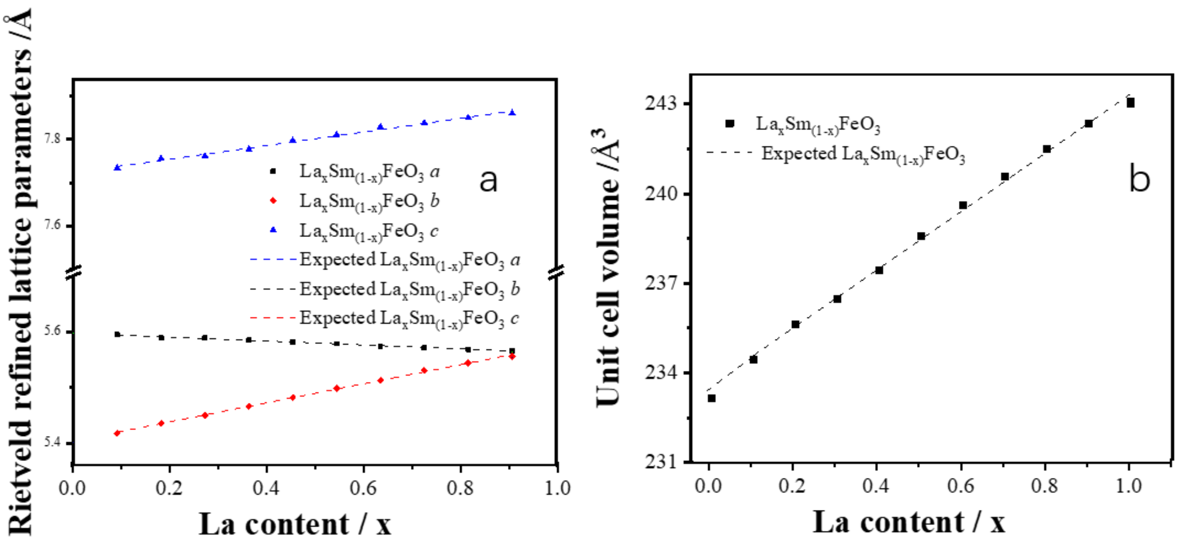

Figure S1 Results of Rietveld analysis of $La_xSm_{1-x}FeO_3$: a) Lattice parameters; b) Unit cell volumes

2. $La_xGd_{1-x}FeO_3$:

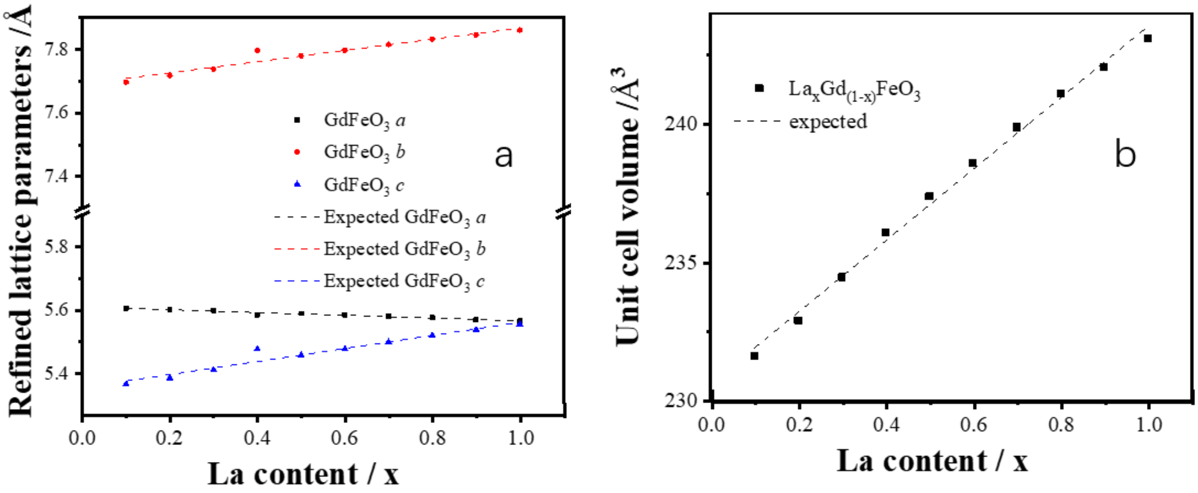

Figure S2 Results of Rietveld analysis of $La_xGd_{1-x}FeO_3$: a) Lattice parameters; b) Unit cell volumes

Solid solutions As shown in Figures S1, S2, S6 and S7, the as-prepared $La_xSm_{1-x}FeO_3$, $La_xGd_{1-x}FeO_3$ samples have similar X-ray diffraction patterns, which can all be assigned to an orthorhombic perovskite structure (*Pbnm*). These samples are still solid solutions. The substitution of the smaller ion Sm^{3+} and Gd^{3+} induce changes of the cell parameters like Nd^{3+} did, the value of *a* and *c* become increasingly different and the cell volume linearly decreases with increased Sm^{3+} and Gd^{3+} content.

3. $La_xHo_{1-x}FeO_3$:

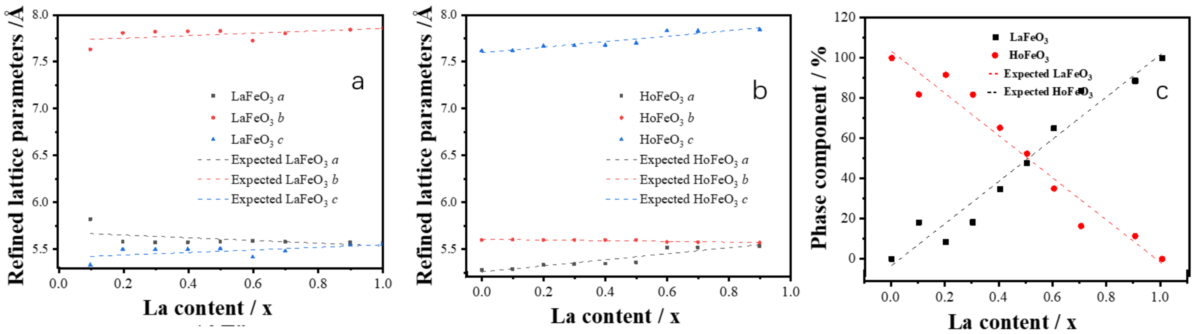

Figure S3 Results of Rietveld analysis of $La_xHo_{1-x}FeO_3$: a , b) Lattice parameters of $LaFeO_3$ phase and $HoFeO_3$ phase, respectively; c) Phase component

4. $La_xYb_{1-x}FeO_3$:

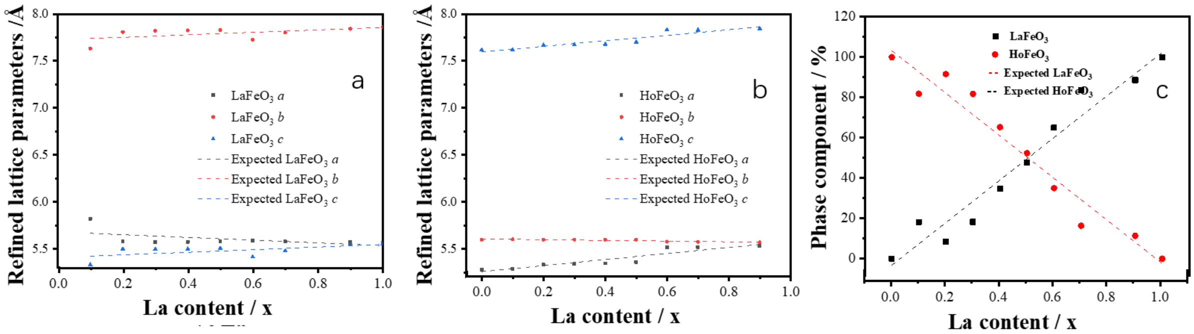

Figure S4 Results of Rietveld analysis of $La_xYb_{1-x}FeO_3$: a , b) Lattice parameters of $LaFeO_3$ phase and $YbFeO_3$ phase, respectively; c) Phase component

Separate phases Powder XRD patterns shown in Figures S8, S9, S10 reveal that the $La_x\mathit{RE}_{1-x}FeO_3$ materials (RE = Ho, Er, Yb) all crystallise as two different perovskite phases, which can initially be assigned as $LaFeO_3$ and *RE*FeO_3. This is confirmed by the evolution of three *RE*FeO_3 peaks (020, 112, 021) between 36° and 43° 2θ and the $LaFeO_3$ peak [002] at 37.6° 2θ. However, these signature peaks begin to shift as the composition of the samples change, with *RE*FeO_3 peaks shifting to lower angles (expanding lattice) and $LaFeO_3$ peaks shifting to higher angles (contracting lattice). This peak shift is consistent with a substitution of La into the *RE*FeO_3 lattice and *RE* into the $LaFeO_3$ lattice, suggesting that while there are two distinct phases, they are both up taking the other A-site metal. Rietveld analysis was undertaken to further confirm the component of the samples. These results suggest that, although there are two distinct phases, some La^{3+} ions enter into the *RE*FeO_3 structure.

S2: Rietveld fitted powder X-ray patterns and structure details

1. $La_xNd_{1-x}FeO_3$:

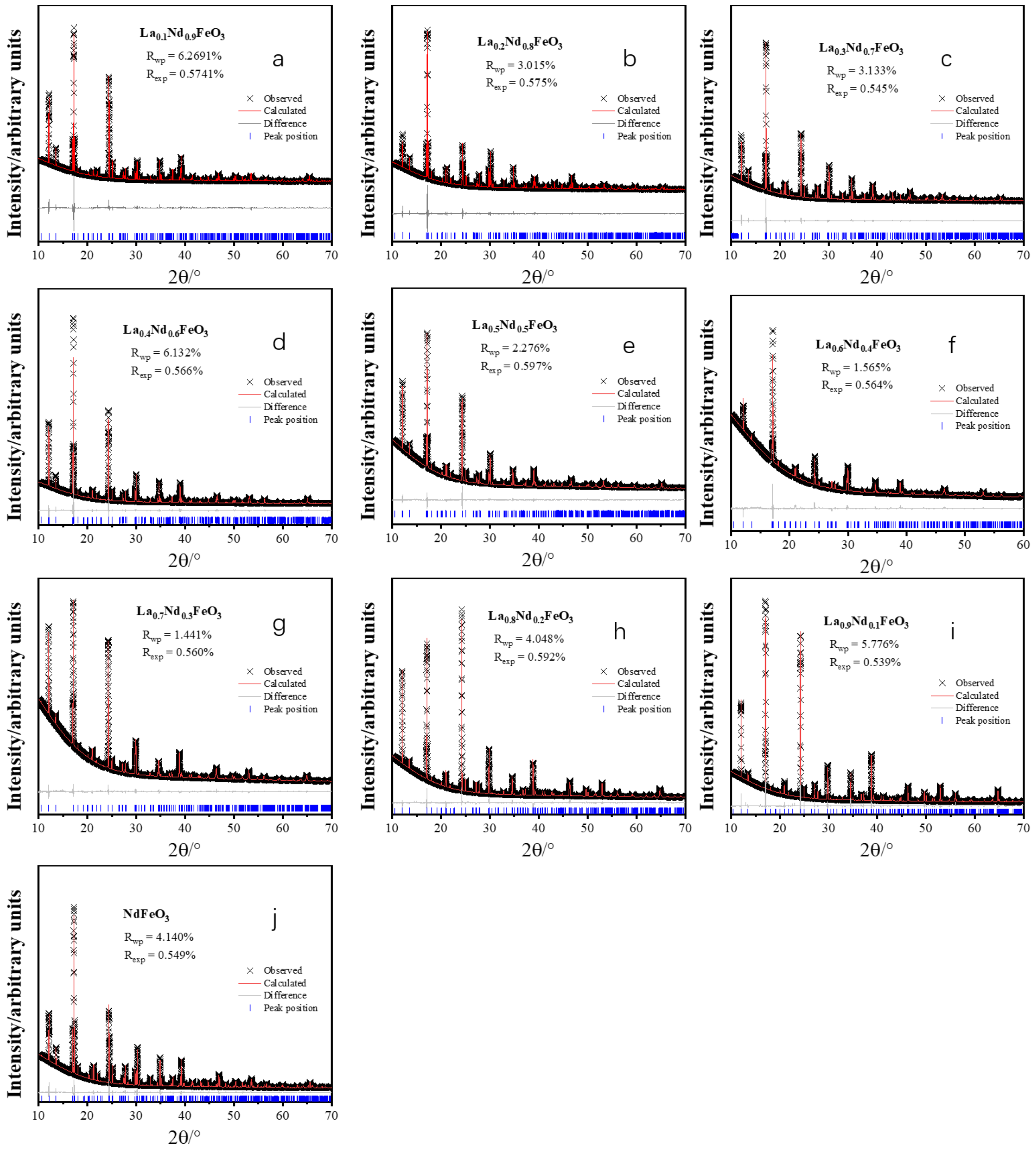

Figure S5 Rietveld fitted X-ray powder patterns (a, .., j) of $La_xNd_{1-x}FeO_3$ (x = 0.1, 0.2, 0.3, ..., 0.9, 1)

The literature used to compare with the obtained $NdFeO_3$ and $LaFeO_3$ is same as the one mentioned in the paper.

Table S1 Structured details obtained from Rietveld refinements against synchrotron X-ray diffraction for $La_xNd_{1-x}FeO_3$

Literature $NdFeO_3$	a = 5.591Å	b = 7.768 Å	c = 5.446 Å	V = 236.22 Å^3
$NdFeO_3$	R_{wp}=3.0538		R_{exp}=0.5735	
x=1	a = 5.587188 (41) Å	b= 7.764159 (70) Å	c= 5.452616 (52) Å	V = 236.533 (4) Å^3
Atom	x	y	z	
Nd	0.04738 (8)	0.25	0.99138 (22)	
Fe	0	0	0.5	
O(1)	0.78650 (92)	0.25	0.92125 (101)	
O(2)	0.29454 (77)	0.05111 (47)	-0.29705 (79)	
$La_{0.1}Nd_{0.9}FeO_3$	R_{wp}=3.8541		R_{exp}=0.5631	
x=0.9	a = 5.583916 (40) Å	b= 7.77775 (61) Å	c= 5.46435 (52) Å	V = 237.319 (3) Å^3
Atom	x	y	z	
La/Nd	0.04526(9)	0.25	0.00764 (23)	
Fe	0	0	0.5	
O(1)	0.45790 (134)	0.25	0.92474 (113)	
O(2)	0.28776 (104)	0.04338 (46)	-0.29810 (103)	
$La_{0.2}Nd_{0.8}FeO_3$	R_{wp}=2.9351		R_{exp}=0.5633	
x=0.8	a = 5.580233 (34) Å	b= 7.78967 (59) Å	c= 5.47505 (44) Å	V = 237.991 (3) Å^3
Atom	x	y	z	
La/Nd	0.04494 (8)	0.25	0.00894 (18)	
Fe	0	0	0.5	
O(1)	0.47952 (99)	0.25	0.92257 (112)	
O(2)	0.29291 (83)	0.04361 (59)	-0.29457 (85)	
$La_{0.3}Nd_{0.7}FeO_3$	R_{wp}=2.5693		R_{exp}=0.5676	
x=0.7	a = 5.578568 (32) Å	b= 7.79816 (52) Å	c= 5.48615 (41) Å	V = 238.662 (3) Å^3
Atom	x	y	z	
La/Nd	0.04337 (7)	0.25	0.00862 (15)	
Fe	0	0	0.5	
O(1)	0.47764 (85)	0.25	0.92922 (94)	
O(2)	0.28466 (74)	0.03631 (45)	-0.29122 (76)	
$La_{0.4}Nd_{0.6}FeO_3$	R_{wp}= 1.7753		R_{exp}= 0.5847	
x=0.6	a = 5.576030 (61) Å	b= 7.80917 (95) Å	c= 5.49757 (73) Å	V = 239.387 (5) Å^3
Atom	x	y	z	
La/Nd	0.04111 (7)	0.25	0.00711 (20)	
Fe	0	0	0.5	

O(1)	0.47222 (103)	0.25	0.92824 (108)	
O(2)	0.28300 (91)	0.04055 (50)	-0.29065 (97)	
$La_{0.5}Nd_{0.5}FeO_3$	R_{wp}= 4.8056		R_{exp}= 0.5552	
x=0.5	*a* = 5.573295 (60) Å	*b*= 7.81727 (96) Å	*c*= 5.50784 (75) Å	V = 239.966 (5) Å^3
Atom	x	y	z	
La/Nd	0.03901 (12)	0.25	0.00691 (31)	
Fe	0	0	0.5	
O(1)	0.47301 (168)	0.25	0.92026 (167)	
O(2)	0.29219 (133)	0.03362 (89)	-0.29143 (136)	
$La_{0.6}Nd_{0.4}FeO_3$	R_{wp}= 1.2658		R_{exp}= 0.5920	
x=0.4	*a* = 5.572468 (21) Å	*b*= 7.82724 (89) Å	*c*= 5.51837 (43) Å	V = 240.695 (10) Å^3
Atom	x	y	z	
La/Nd	0.03839 (13)	0.25	0.00741 (37)	
Fe	0	0	0.5	
O(1)	0.48546 (253)	0.25	0.93859 (521)	
O(2)	0.29042 (198)	0.04249 (129)	-0.28478 (208)	
$La_{0.7}Nd_{0.3}FeO_3$	R_{wp}= 1.1653		R_{exp}= 0.5884	
x=0.3	*a* = 5.569836 (62) Å	*b*= 7.83680 (10) Å	*c*= 5.52748 (74) Å	V = 241.273 (5) Å^3
Atom	x	y	z	
La/Nd	0.03531 (7)	0.25	0.00751 (17)	
Fe	0	0	0.5	
O(1)	0.48306 (89)	0.25	0.93606 (124)	
O(2)	0.27822 (109)	0.04642 (69)	-0.27951 (119)	
$La_{0.8}Nd_{0.2}FeO_3$	R_{wp}= 1.3192		R_{exp}= 0.5903	
x=0.2	*a* = 5.568036 (50) Å	*b*= 7.84637 (88) Å	*c*= 5.53685 (69) Å	V = 241.899 (5) Å^3
Atom	x	y	z	
La/Nd	0.03409 (9)	0.25	0.00555 (21)	
Fe	0	0	0.5	
O(1)	0.48500 (98)	0.25	0.95027 (177)	
O(2)	0.26459 (106)	0.05545 (56)	-0.26590 (118)	
$La_{0.9}Nd_{0.1}FeO_3$	R_{wp}= 4.2022		R_{exp}= 0.5686	
x=0.1	*a* = 5.568016 (45) Å	*b*= 7.85501 (96) Å	*c*= 5.54667 (71) Å	V = 242.594 (5) Å^3
Atom	x	y	z	
La/Nd	0.03153 (12)	0.25	0.00749 (23)	
Fe	0	0	0.5	
O(1)	0.45132 (259)	0.25	0.89931 (213)	

O(2)	0.27208 (193)	0.03191 (134)	-0.28163 (210)	
$LaFeO_3$	R_{wp}= 4.1152		R_{exp}= 0.5685	
x=0	*a* = 5.565973 (30) Å	*b*= 7.860953(88) Å	*c*= 5.555669(43) Å	V = 238.598 (6) $Å^3$
Atom	x	y	z	
La	0.03328 (5)	0.25	-0.00673 (12)	
Fe	0	0	0.5	
O(1)	0.48091 (60)	0.25	0.06984 (82)	
O(2)	0.28056 (59)	0.04354 (46)	-0.28061 (64)	
Literature $LaFeO_3$	*a* = 5.563(2) Å	*b* = 7.867(3) Å	*c* = 5.553(2) Å	V = 243.02 $Å^3$

2. $La_xSm_{1-x}FeO_3$:

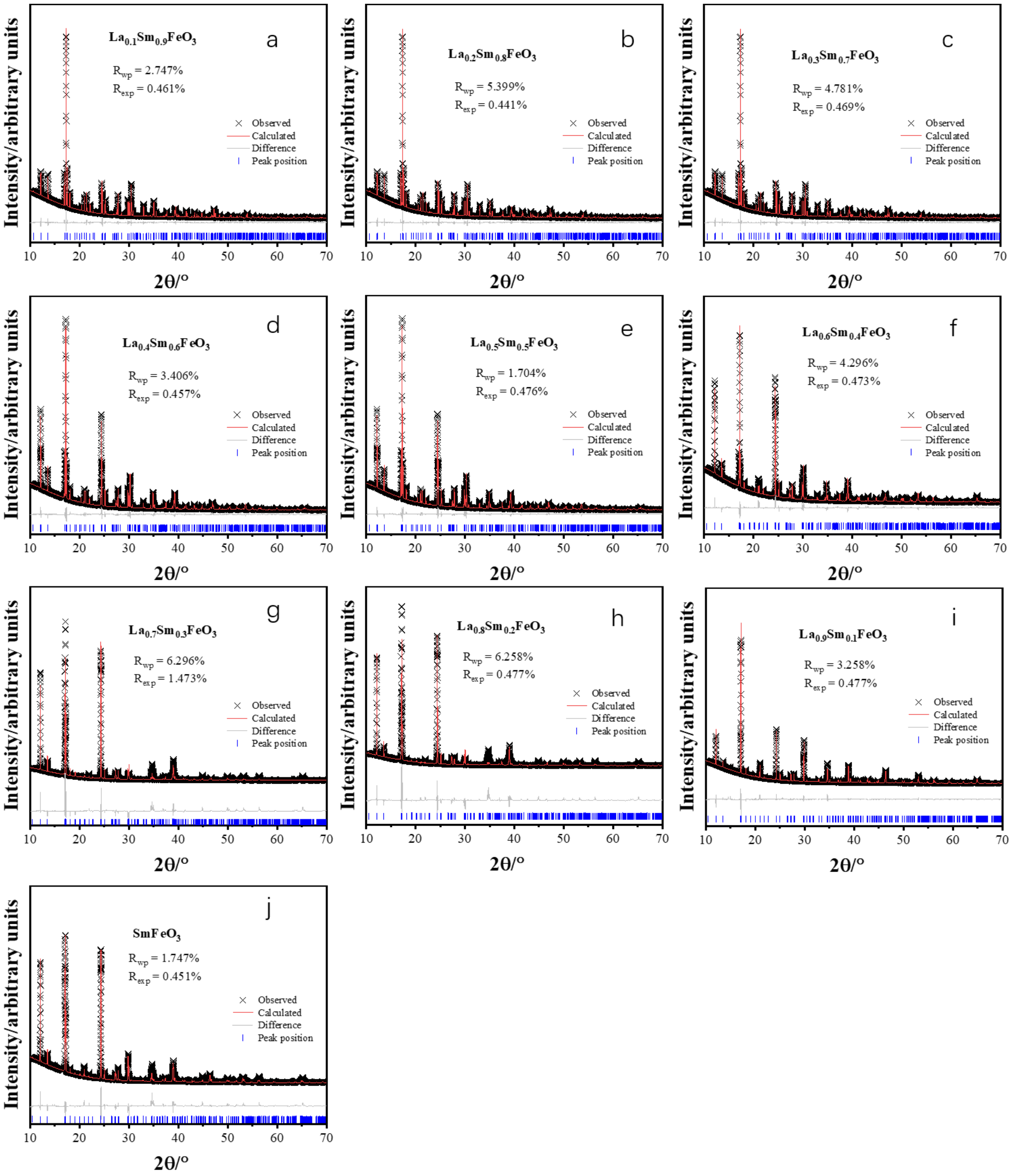

Figure S6 Rietveld fitted X-ray powder patterns (a, .., j) of $La_xSm_{1-x}FeO_3$ (x = 0.1, 0.2, 0.3, .., 0.9, 1)

Table S2 Structured details obtained from Rietveld refinements against synchrotron X-ray diffraction for $La_xSm_{1-x}FeO_3$

Literature[1] $SmFeO_3$	*a* = 5.616 Å	*b* = 7.668 Å	*c* = 5.346 Å	V = 230.22 Å^3
$SmFeO_3$	R_{wp}=3.5581		R_{exp}=0.5193	
x=1	*a* = 5.599230 (81) Å	*b*= 7.709865 (123) Å	*c*= 5.399771 (34) Å	V = 233.104 (7) Å^3
Atom	x	y	z	
La/ Sm	0.05979 (21)	0.25	0.98561(41)	
Fe	0	0	0.5	
O(1)	0.47718 (247)	0.25	0.06603(301)	
O(2)	0.68811 (295)	-0.06811 (161)	0.26614 (296)	
$La_{0.1}Sm_{0.9}FeO_3$	R_{wp}=2.5814		R_{exp}=0.4618	
x=0.9	*a* = 5.594720 (25) Å	*b*= 7.733752 (35) Å	*c*= 5.418261 (26) Å	V = 234.438 (2) Å^3
Atom	x	y	z	
La/ Sm	0.05467 (14)	0.25	0.98740(15)	
Fe	0	0	0.5	
O(1)	0.47239 (85)	0.25	0.09002(91)	
O(2)	0.69526 (83)	-0.04717 (51)	0.28932 (95)	
$La_{0.2}Sm_{0.8}FeO_3$	R_{wp}= 6.7448		R_{exp}= 0.4412	
x=0.8	*a* = 5.589411 (31) Å	*b*= 7.756050 (49) Å	*c*= 5.436315 (34) Å	V = 235.674 (2) Å^3
Atom	x	y	z	
La/ Sm	0.05209 (10)	0.25	0.98770(20)	
Fe	0	0	0.5	
O(1)	0.47270 (119)	0.25	0.08585(127)	
O(2)	0.70208 (103)	-0.04686 (75)	0.30017 (111)	
$La_{0.3}Sm_{0.7}FeO_3$	R_{wp}= 4.8376		R_{exp}= 0.4691	
x=0.7	*a* = 5.588695 (61) Å	*b*= 7.762107 (69) Å	*c*= 5.450667 (65) Å	V = 236.450 (4) Å^3
Atom	x	y	z	
La/ Sm	0.04508 (24)	0.25	0.99251(41)	
Fe	0	0	0.5	
O(1)	0.50725 (104)	0.25	0.05879(135)	
O(2)	0.72100 (138)	-0.02846 (59)	0.27850 (143)	
$La_{0.4}Sm_{0.6}FeO_3$	R_{wp}= 3.4233		R_{exp}= 0.4577	
x=0.6	*a* = 5.584876 (37) Å	*b*= 7.777330 (52) Å	*c*= 5.466533 (40) Å	V = 237.441 (3) Å^3
Atom	x	y	z	
La/ Sm	0.04461 (7)	0.25	0.99144(21)	
Fe	0	0	0.5	

O(1)	0.48462 (67)	0.25	0.06830(78)	
O(2)	0.71336 (67)	-0.03927 (40)	0.29125 (70)	
$La_{0.5}Sm_{0.5}FeO_3$	R_{wp}= 1.7368		R_{exp}= 0.4766	
x=0.5	*a* = 5.581479 (27) Å	*b*= 7.797565 (38) Å	*c*= 5.481909 (29) Å	V = 238.583 (2) Å^3
Atom	x	y	z	
La/ Sm	0.04361 (6)	0.25	0.99068	
Fe	0	0	0.5	
O(1)	0.48604 (59)	0.25	0.06462(71)	
O(2)	0.71507 (68)	-0.04614 (37)	0.28648 (73)	
$La_{0.6}Sm_{0.4}FeO_3$	R_{wp}= 4.3695		R_{exp}= 0.4739	
x=0.4	*a* = 5.578450 (55) Å	*b*= 7.811315 (67) Å	*c*= 5.498684 (59) Å	V = 239.605 (4) Å^3
Atom	x	y	z	
La/ Sm	0.04113 (12)	0.25	0.99488(45)	
Fe	0	0	0.5	
O(1)	0.49451 (97)	0.25	0.06331(117)	
O(2)	0.71408 (93)	-0.03483 (43)	0.28330 (97)	
$La_{0.7}Sm_{0.3}FeO_3$	R_{wp}= 6.6185		R_{exp}= 0.4257	
x=0.3	*a* = 5.573067 (54) Å	*b*= 7.825561 (90) Å	*c*= 5.511309 (60) Å	V = 240.361 (5) Å^3
Atom	x	y	z	
La/ Sm	0.356 (12)	0.25	0.99228 (35)	
Fe	0	0	0.5	
O(1)	0.49126 (82)	0.25	0.02494 (209)	
O(2)	0.69929 (153)	-0.01592 (241)	0.23557 (220)	
$La_{0.8}Sm_{0.2}FeO_3$	R_{wp}= 1.2515		R_{exp}= 0.4768	
x=0.2	*a* = 5.570734 (34) Å	*b*= 7.836689 (47) Å	*c*= 5.529560 (32) Å	V = 241.399 (2) Å^3
Atom	x	y	z	
La/ Sm	0.03614 (6)	0.25	-0.00702	
Fe	0	0	0.5	
O(1)	0.48878 (62)	0.25	0.06633(88)	
O(2)	0.26906 (96)	0.04108 (56)	-0.27610 (110)	
$La_{0.9}Sm_{0.1}FeO_3$	R_{wp}= 2.5586		R_{exp}= 0.4598	
x=0.1	*a* = 5.543238 (33) Å	*b*= 5.568248 (29) Å	*c*= 7.849198 (44) Å	V = 242.274 (2) Å^3
Atom	x	y	z	
La/ Sm	0.99284 (16)	0.03376(7)	0.25	
Fe	0	0.5	0	
O(1)	0.06197 (161)	0.49544(85)	0.25	

O(2)	0.77537 (114)	0.23105 (103)	-0.04260 (77)	
$LaFeO_3$	R_{wp}= 4.1152		R_{exp}= 0.5685	
x=0	*a* = 5.565973 (30) Å	*b*= 7.860953(88) Å	*c*= 5.555669(43) Å	V = 238.598 (6) Å^3
Atom	x	y	z	
La	0.03328 (5)	0.25	-0.00673 (12)	
Fe	0	0	0.5	
O(1)	0.48091 (60)	0.25	0.06984 (82)	
O(2)	0.28056 (59)	0.04354 (46)	-0.28061 (64)	
Literature $LaFeO_3$	*a* = 5.563(2) Å	*b* = 7.867(3) Å	*c* = 5.553(2) Å	V = 243.02 Å^3

3. $La_xGd_{1-x}FeO_3$:

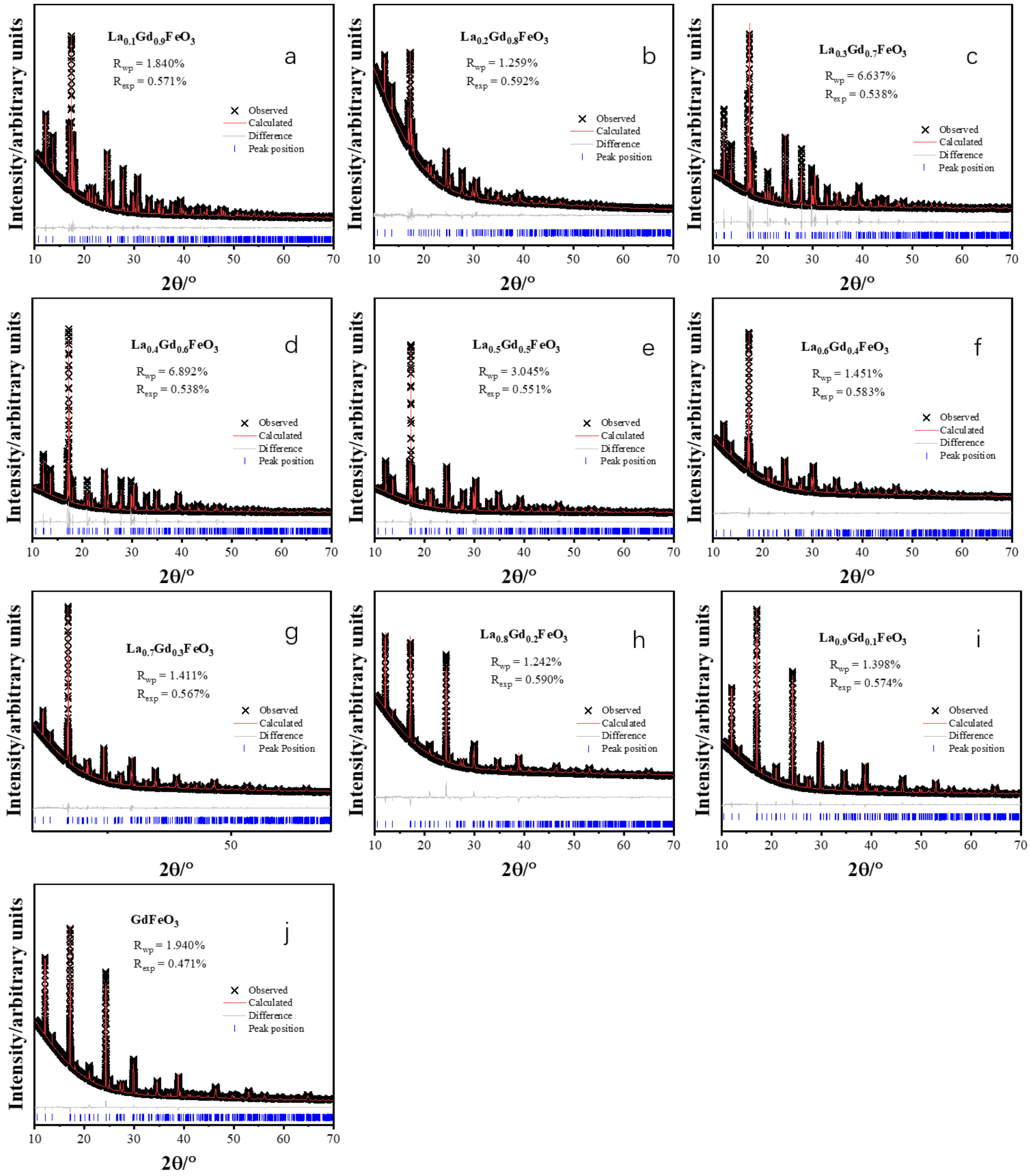

Figure S7 Rietveld fitted X-ray powder patterns (a, …, j) of $La_xNd_{1-x}FeO_3$ (x = 0.1, 0.2, 0.3, …, 0.9, 1)

Table S3 Structural details obtained from Rietveld refinements against synchrotron X-ray diffraction for $La_xGd_{1-x}FeO_3$

Literature $GdFeO_3$[2]	*a* = 5.616 Å	*b* = 7.668 Å	*c* = 5.346 Å	V = 230.22 Å^3
$La_{0.1}Gd_{0.9}FeO_3$	R_{wp}=1.8405		R_{exp}=0.5715	
x=0.9	*a* = 5.606104 (50) Å	*b*= 7.697633(87) Å	*c*= 5.367555(57) Å	V = 231.630(4) Å^3
Atom	x	y	z	
La/Gd	0.06041	0.25	-0.01539(12)	
Fe	0	0	0.5	
O(1)	0.46491(104)	0.25	0.09330(97)	
O(2)	0.29546(77)	0.05640(67)	-0.30246(77)	
$La_{0.2}Gd_{0.8}FeO_3$	R_{wp}=1.2604		R_{exp}=0.5920	
x=0.8	*a* = 5.603362(165) Å	*b*= 7.718574(263) Å	*c*= 5.386467(182) Å	V = 232.964(13) Å^3
Atom	x	y	z	
La/Gd	0.05755(12)	0.25	-0.01565 (27)	
Fe	0	0	0.5	
O(1)	0.44982 (203)	0.25	0.07480 (188)	
O(2)	0.28912 (129)	0.05639 (126)	-0.30165 (147)	
$La_{0.3}Gd_{0.7}FeO_3$	R_{wp}=6.6505		R_{exp}=0.5380	
x=0.7	*a* = 5.599303 (134) Å	*b*= 7.738688(234) Å	*c*= 5.411840(159) Å	V = 234.502 (11) Å^3
Atom	x	y	z	
La/Gd	0.05541 (15)	0.25	- 0.01392 (35)	
Fe	0	0	0.5	
O(1)	0.47151 (230)	0.25	0.07879 (269)	
O(2)	0.29028 (163)	0.06696 (159)	-0.29650 (194)	
$La_{0.4}Gd_{0.6}FeO_3$	R_{wp}= 6.8352		R_{exp}= 0.5382	
x=0.6	*a* = 5.594657 (137) Å	*b*= 7.762445 (225) Å	*c*= 5.437005 (169) Å	V = 236.119 (12) Å^3
Atom	x	y	z	
La/Gd	0.05292 (16)	0.25	-0.01157 (43)	
Fe	0	0	0.5	
O(1)	0.47427 (219)	0.25	0.08276 (272)	
O(2)	0.29246 (175)	0.05417 (132)	-0.29662 (207)	
$La_{0.5}Gd_{0.5}FeO_3$	R_{wp}= 3.0582		R_{exp}= 0.5514	
x=0.5	*a* = 5.590243 (52) Å	*b*= 7.779733 (88) Å	*c*= 5.458971 (64) Å	V = 237.414 (4) Å^3
Atom	x	y	z	
La/Gd	0.04878 (7)	0.25	-0.01107 (18)	
Fe	0	0	0.5	

O(1)	0.48040 (93)	0.25	0.08121 (116)	
O(2)	0.28807 (83)	0.04365 (61)	-0.29536 (90)	
$La_{0.6}Gd_{0.4}FeO_3$	R_{wp}= 1.4262		R_{exp}= 0.5837	
x=0.4	*a* = 5.585722 (74) Å	*b*= 7.797551 (117) Å	*c*= 5.478096 (88) Å	V = 238.598 (6) $Å^3$
Atom	x	y	z	
La/Gd	0.04637 (8)	0.25	-0.01032 (22)	
Fe	0	0	0.5	
O(1)	0.48009 (103)	0.25	0.08623 (137)	
O(2)	0.29074 (89)	0.04594 (63)	-0.29310 (99)	
$La_{0.7}Gd_{0.3}FeO_3$	R_{wp}= 1.3621		R_{exp}= 0.5769	
x=0.3	*a* = 5.580813 (82) Å	*b*= 7.815758 (131) Å	*c*= 5.499882 (97) Å	V = 239.895 (7) $Å^3$
Atom	x	y	z	
La/Gd	0.04227 (8)	0.25	-0.00883 (28)	
Fe	0	0	0.5	
O(1)	0.48767 (103)	0.25	0.08167 (156)	
O(2)	0.28590 (104)	0.03985 (71)	-0.28548 (116)	
$La_{0.8}Gd_{0.2}FeO_3$	R_{wp}= 1.3192		R_{exp}= 0.5903	
x=0.2	*a* = 5.575699 (131) Å	*b*= 7.831285 (201) Å	*c*= 5.517637 (148) Å	V = 240.927 (11) $Å^3$
Atom	x	y	z	
La/Gd	0.03667 (10)	0.25	-0.00066 (70)	
Fe	0	0	0.5	
O(1)	0.48528 (106)	0.25	0.04333 (264)	
O(2)	0.26223 (128)	0.05404 (61)	-0.26232 (160)	
$La_{0.9}Gd_{0.1}FeO_3$	R_{wp}= 1.0414		R_{exp}= 0.5743	
x=0.1	*a* = 5.570020 (34) Å	*b*= 7.845915 (58) Å	*c*= 5.538204 (43) Å	V = 242.030 (3) $Å^3$
Atom	x	y	z	
La/Gd	0.03328 (5)	0.25	-0.00673 (12)	
Fe	0	0	0.5	
O(1)	0.48091 (60)	0.25	0.06984 (82)	
O(2)	0.28056 (59)	0.04354 (46)	-0.28061 (64)	
$LaFeO_3$	R_{wp}= 4.1152		R_{exp}= 0.5685	
x=0	*a* = 5.565973 (30) Å	*b*= 7.860953(88) Å	*c*= 5.555669(43) Å	V = 238.598 (6) $Å^3$
Atom	x	y	z	
La	0.03328 (5)	0.25	-0.00673 (12)	
Fe	0	0	0.5	
O(1)	0.48091 (60)	0.25	0.06984 (82)	

O(2)	0.28056 (59)	0.04354 (46)	-0.28061 (64)	
Literature $LaFeO_3$	*a* = 5.563(2) Å	*b* = 7.867(3) Å	*c* = 5.553(2) Å	V = 243.02 $Å^3$

4. $La_xHo_{1-x}FeO_3$:

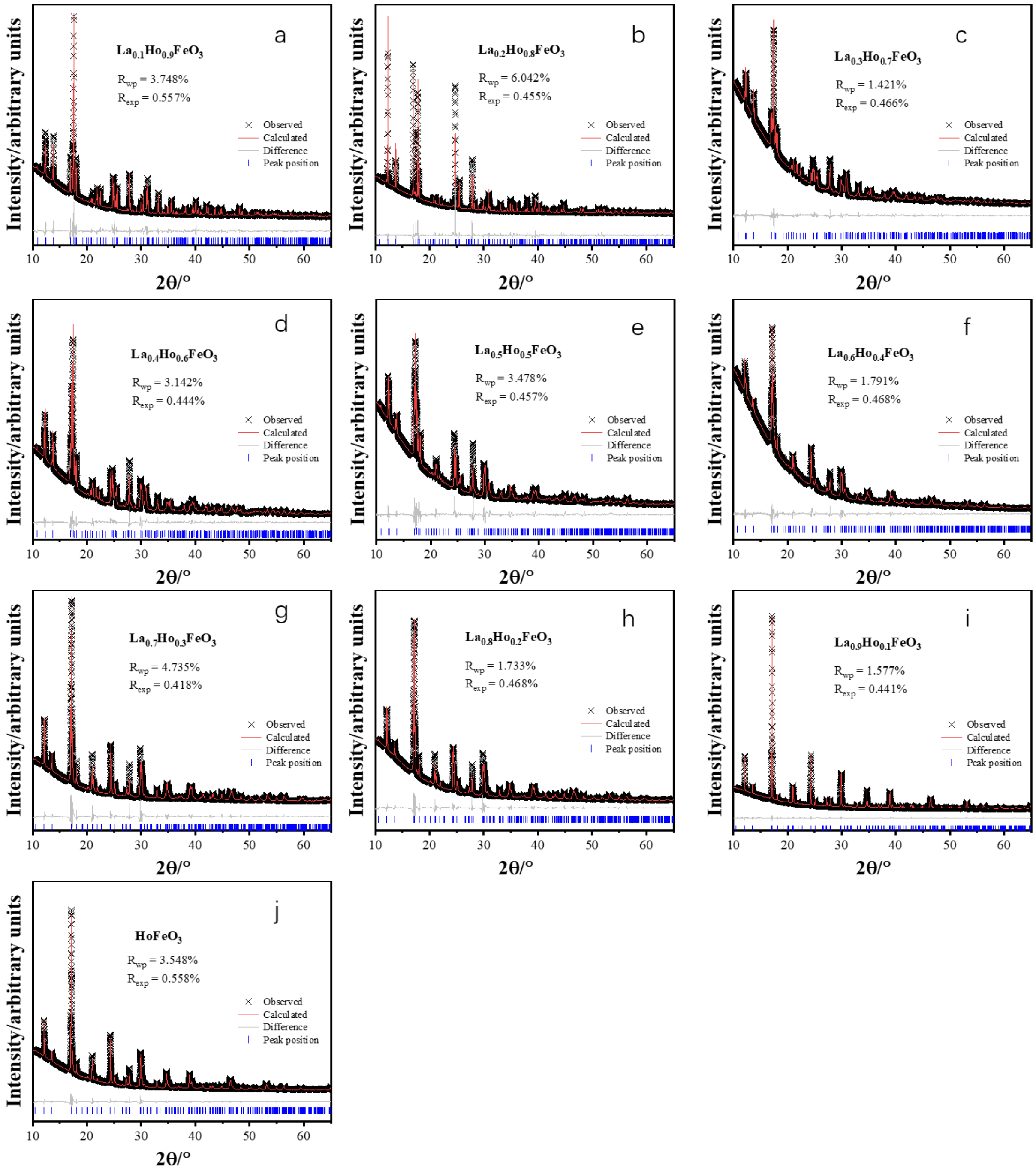

Figure S8 Rietveld fitted X-ray powder patterns (a, .., j) of $La_xHo_{1-x}FeO_3$ (x = 0.1, 0.2, 0.3, .., 0.9, 1)

Table S4 Structural details obtained from Rietveld refinements against synchrotron X-ray diffraction for $La_xHo_{1-x}FeO_3$

**Compounds**	$HoFeO_3$(literature)[2]	$HoFeO_3$	$La_{0.1}Ho_{0.9}FeO_3$	$La_{0.2}Ho_{0.8}FeO_3$	$La_{0.3}Ho_{0.7}FeO_3$	$La_{0.4}Ho_{0.6}FeO_3$	$La_{0.5}Ho_{0.5}FeO_3$
**Percent of phases**							
$LaFeO_3$ (%)		0	9.01	12.62	24.84	45.86	48.31
$HoFeO_3$ (%)		100	91.91	87.38	75.16	54.14	51.69
**Lattice parameters**							
$HoFeO_3$							
a /Å	5.2819	5.296217(21)	5.287266(81)	5.331317(62)	5.339455(126)	5.345459(164)	5.360902(311)
b/Å	5.5983	5.597836(14)	5.601545(72)	5.598455(63)	5.598330 (111)	5.598744(144)	5.598046(271)
c /Å	7.6177	7.62230(28)	7.619015(108)	7.668721(163)	7.671526(170)	7.679246(218)	7.701790(666)
Cell volume/Å^3	225.25	225.436(2)	225.651(6)	228.889(6)	229.317(9)	229.823(11)	231.135(27)
$LaFeO_3$							
a /Å			5.821870(848)	5.580439(1946)	5.573672(455)	5.574716(233)	5.578367(261)
b /Å			7.633079(1374)	7.808711(2940)	7.822038(640)	7.825247(348)	7.829924(345)
c /Å			5.331480(923)	5.499490(1659)	5.497686(412)	5.500729(219)	5.507554(241)
Cell volume/Å^3			236.925(68)	239.646(143)	239.824(39)	239.961(17)	240.560(19)
**R_{wp} (%)**		2.1062	4.5774	6.0366	1.4205	3.1418	3.4779
**R_{exp} (%)**		0.4703	0.4589	0.4554	0.4665	0.4441	0.4574

compounds	$La_{0.6}Ho_{0.4}FeO_3$	$La_{0.7}Ho_{0.3}FeO_3$	$La_{0.8}Ho_{0.2}FeO_3$	$La_{0.9}Ho_{0.1}FeO_3$	$LaFeO_3$	$LaFeO_3$(simulated)

**Percent of phases**						
$LaFeO_3$ (%)	52.89	65.21	86.21	92.81	0	
$HoFeO_3$ (%)	47.11	34.79	13.79	7.19	100	
**Lattice parameters**						
$HoFeO_3$						
a /Å	5.515694(185)	5.457845(1562)	5.512724(13208)	5.545255(87)		
b/Å	5.576162(212)	5.541365(1872)	5.538031(1435)	5.573912(84)		
c /Å	7.832618(280)	7.757504(2446)	7.799779(2351)	7.843348(148)		
Cell volume/$Å^3$	240.903(15)	234.617(128)	238.124(90)	242.428(7)		
$LaFeO_3$						
a/Å	5.589660(335)	5.579038(169)	5.573456(205)	5.570057(42)	5.565973(30)	5.5621
b/Å	7.727505(753)	7.803333(272)	7.830499(277)	7.844113(62)	7.860953(88)	7.8516
c/Å	5.413968(465)	5.479394(166)	5.522800(247)	5.532169(43)	5.555669(43)	5.5520
Cell volume /$Å^3$	233.852(20)	238.546 (13)	241.093(16)	241.712(3)	243.082(4)	242.46
**R_{wp} (%)**	1.7654	4.7245	1.4559	1.5319	2.9491	
**R_{exp} (%)**	0.4688	0.4185	0.4634	0.4419	0.4051	

The space groups used for the refinement for $HoFeO_3$ and $LaFeO_3$ were *Pbnm* and *Pnma*, respectively.

5. $La_xEr_{1-x}FeO_3$:

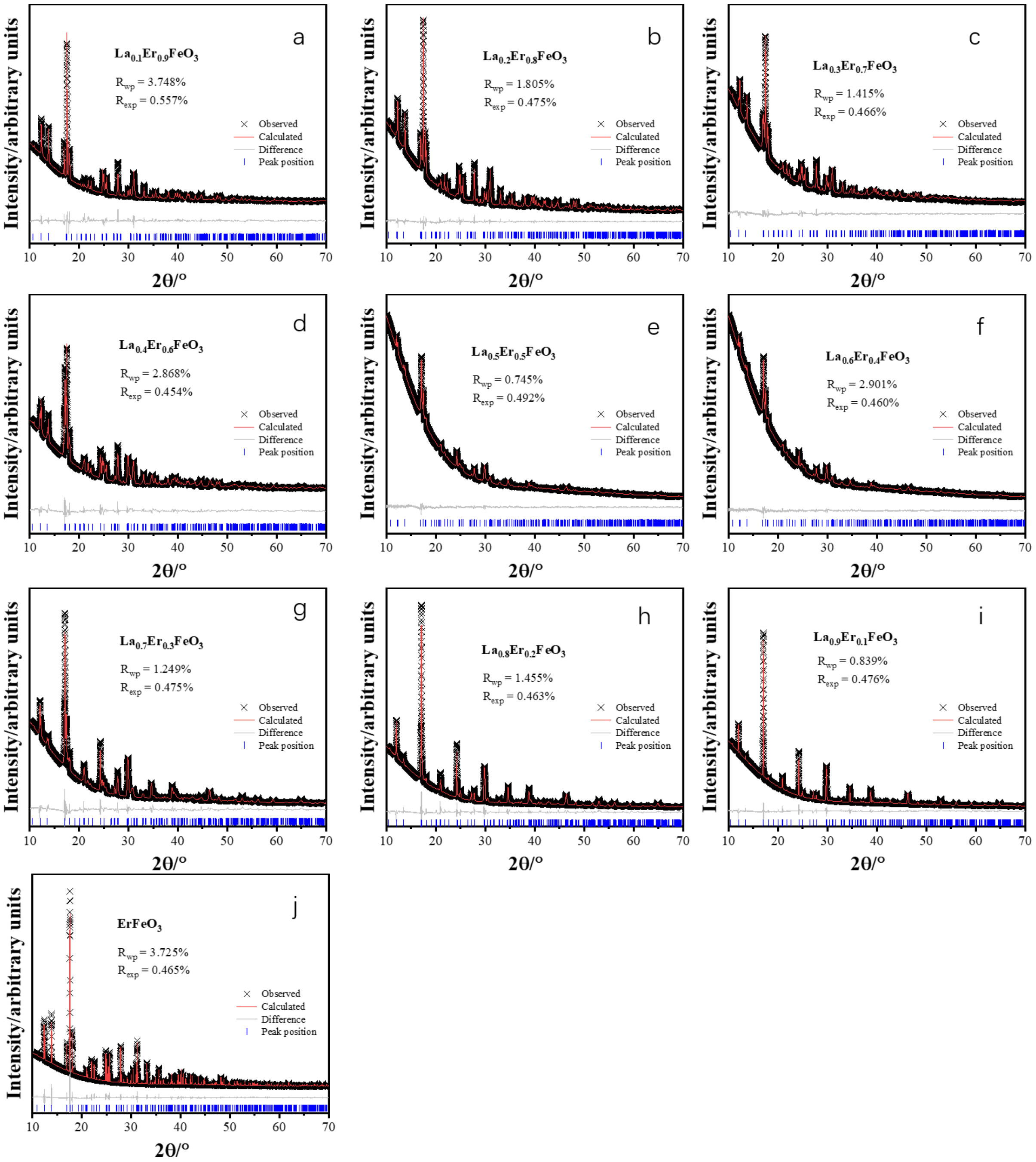

Figure S9 Rietveld fitted X-ray powder patterns (a, …, j) of $La_xEr_{1-x}FeO_3$ (x = 0.1, 0.2, 0.3, …, 0.9, 1)

The literature used to compare with the obtained $ErFeO_3$ is same as the one mentioned in the paper.

Table S5 Structural details obtained from Rietveld refinements against synchrotron X-ray diffraction for $La_xEr_{1-x}FeO_3$

**Compounds**	$ErFeO_3$(literature)	$ErFeO_3$	$La_{0.1}Er_{0.9}FeO_3$	$La_{0.2}Er_{0.8}FeO_3$	$La_{0.3}Er_{0.7}FeO_3$	$La_{0.4}Er_{0.6}FeO_3$	$La_{0.5}Er_{0.5}FeO_3$
**Percent of phases**							
$LaFeO_3$ (%)		0	6.44	12.64	34.64	45.86	60.65
$ErFeO_3$ (%)		100	93.56	87.36	65.36	54.14	39.35
**Lattice parameters**							
$ErFeO_3$							
a /Å	5.2648	5.266287(31)	5.293910(261)	5.303884(117)	5.303960(159)	5.302832(437)	5.304687(458)
b/Å	5.5938	5.594936(34)	5.595024(298)	5.598643(122)	5.599652(166)	5.598186(448)	5.601407(434)
c /Å	7.6080	7.600250(48)	7.630913(420)	7.642792(167)	7.641607(235)	7.638303(574)	7.652237(612)
Cell volume/$Å^3$	224.06	223.938(2)	226.024(021)	226.949(9)	226.958(12)	226.753(1)	227.376(32)
$LaFeO_3$							
a /Å			5.532902(4779)	5.550950(845)	5.573966(507)	5.573745(359)	5.574852(359)
b /Å			7.834262(2259)	7.834527(1519)	7.822673(722)	7.837643(522)	7.836511(522)
c /Å			5.538940(4960)	5.523402(0852)	5.500133(546)	5.524803(305)	5.524956(305)
Cell volume/$Å^3$			240.092(307)	240.208(70)	239.824(39)	241.351(26)	241.351(26)
**R_{wp} (%)**		3.7259	3.7489	1.8329	1.4827	2.9006	0.7743
**R_{exp} (%)**		0.4657	0.5577	0.4757	0.4662	0.4549	0.4928

compounds	$La_{0.6}Er_{0.4}FeO_3$	$La_{0.7}Er_{0.3}FeO_3$	$La_{0.8}Er_{0.2}FeO_3$	$La_{0.9}Er_{0.1}FeO_3$	$LaFeO_3$	$LaFeO_3$(simulated)
**Percent of phases**						
$LaFeO_3$ (%)	54.25	55.60	68.90	96.08	0	
$ErFeO_3$ (%)	45.75	44.39	31.10	3.92	100	
**Lattice parameters**						
$ErFeO_3$						
a /Å	5.339765(542)	5.457845(1562)	5.512724(1308)	5.392775(1130)		
b/Å	5.592162(659)	5.541365(1872)	5.538031(1128)	5.522649(1760)		
c /Å	7.691834(761)	7.757504(2446)	7.799779(1665)	7.900650(2051)		
Cell volume/Å^3	229.685(42)	234.617(128)	238.124(90)	235.300(109)		
$LaFeO_3$						
a/Å	5.573918(257)	5.573956(183)	5.573456(205)	5.572331(261)	5.565973(30)	5.5621
b/Å	7.830421(361)	7.831784(281)	7.830499(277)	7.845341(361)	7.860953(88)	7.8516
c/Å	5.513369(293)	5.521023(280)	5.522800(247)	5.534194(256)	5.555669(43)	5.5520
Cell volume /Å^3	240.637(20)	241.015(17)	241.093(16)	241.937(19)	243.082(4)	242.46
**R_{wp} (%)**	2.3946	1.2758	1.4559	0.8611	2.9491	
**R_{exp} (%)**	0.4602	0.4759	0.4634	0.4763	0.4051	

The space group used for the refinement for $ErFeO_3$ and $LaFeO_3$ are *Pbnm*, *Pnma*, respectively.

6. $La_xYb_{1-x}FeO_3$:

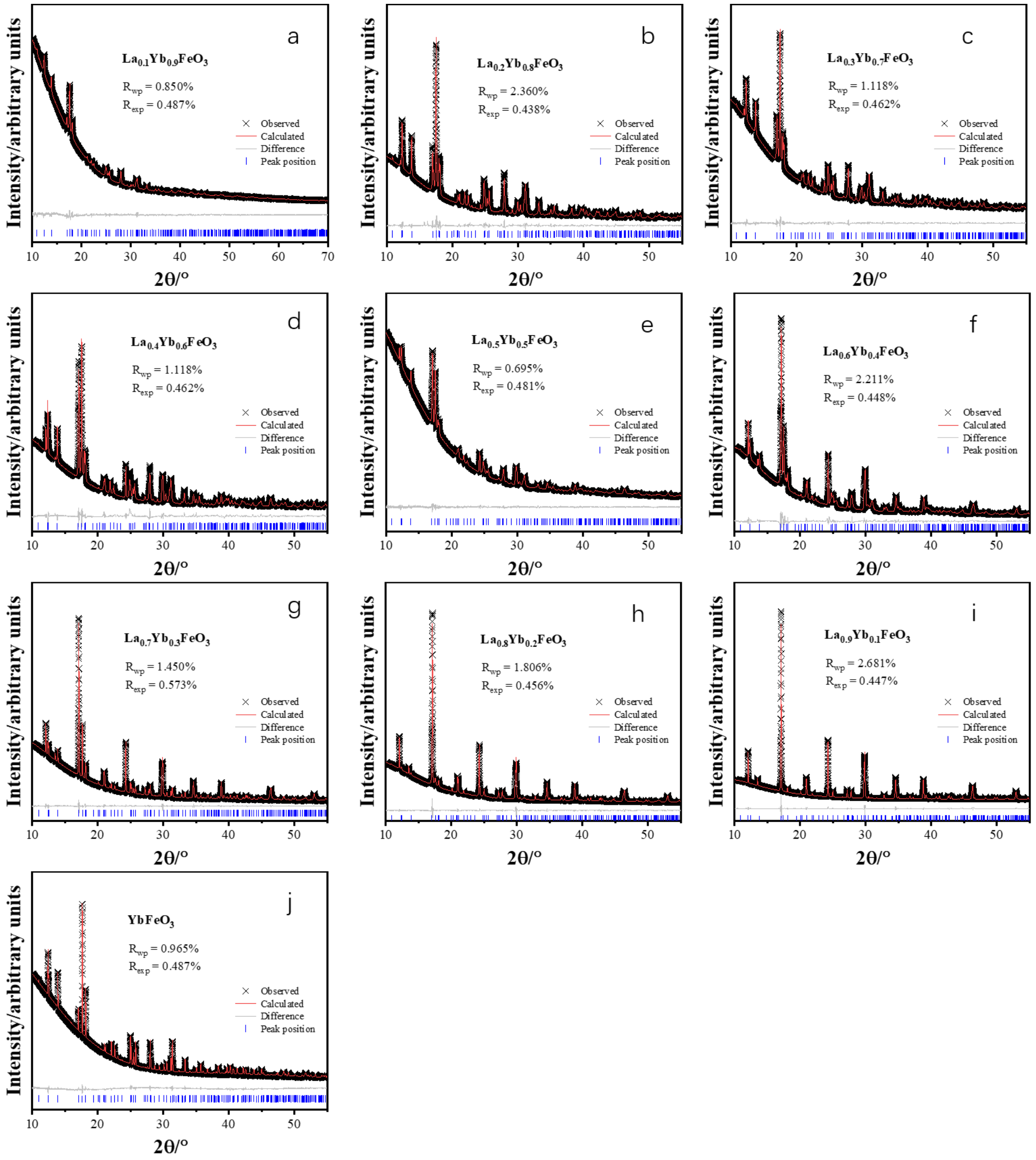

Figure S10 Rietveld fitted X-ray powder patterns (a,.., j) of $La_xYb_{1-x}FeO_3$ (x = 0.1, 0.2, 0.3,.., 0.9, 1)

Table S6 Structural details obtained from Rietveld refinements against synchrotron X-ray diffraction for $La_xYb_{1-x}FeO_3$

**Compounds**	$YbFeO_3$(literature)[2]	$YbFeO_3$	$La_{0.1}Yb_{0.9}FeO_3$	$La_{0.2}Yb_{0.8}FeO_3$	$La_{0.3}Yb_{0.7}FeO_3$	$La_{0.4}Yb_{0.6}FeO_3$	$La_{0.5}Yb_{0.5}FeO_3$
**Percent of phases**							
$LaFeO_3$ (%)		0	3.82	6.49	16.61	38.31	40.56
$YbFeO_3$ (%)		100	96.18	93.51	83.39	61.69	59.44
**Lattice parameters**							
$YbFeO_3$							
a /Å	5.233	5.232264(48)	5.260070(396)	5.280682(149)	5.287258(127)	5.282537(297)	5.293010(252)
b /Å	5.557	5.565325(55)	5.572088(363)	5.575580(140)	5.576735(123)	5.575078(328)	5.578088(246)
c / Å	7.570	7.581240(78)	7.603034(569)	7.628171(221)	7.638711(196)	7.633057(454)	7.647083(382)
Cell volume /$Å^3$	220.13	220.760(4)	222.842(28)	224.595(11)	224.595 (9)	224.798(23)	225.779(18)
$LaFeO_3$							
a / Å			5.373815(1654)	5.573927(521)	5.571436(406)	5.568332(345)	5.569460(303)
b / Å			8.046701(2148)	7.824423(590)	7.845292(519)	7.844495(540)	7.849872(426)
c /Å			5.545994(1654)	5.513924(443)	5.521134(345)	5.525797(351)	5.526387(277)
Cell volume /$Å^3$			239.804(121)	240.477(35)	241.326(28)	241.371(27)	241.611(22)
R_{wp} **(%)**		0.9656	0.8562	2.4191	1.1768	2.9448	0.7069
R_{exp} **(%)**		0.4874	0.4874	0.4386	0.4631	0.5410	0.4820

Compounds	$La_{0.6}Yb_{0.4}FeO_3$	$La_{0.7}Yb_{0.3}FeO_3$	$La_{0.8}Yb_{0.2}FeO_3$	$La_{0.9}Yb_{0.1}FeO_3$	$LaFeO_3$	$LaFeO_3$(simulated)
**Percent of phases**						
$LaFeO_3$ (%)	51.27	77.60	84.03	84.61	100	
$YbFeO_3$ (%)	48.73	22.40	15.97	15.39	0	
**Lattice parameters**						
$YbFeO_3$						
a /Å	5.310104(388)	5.238964(104)	5.272157(1168)	5.221929(4829)		
b /Å	5.576189(310)	5.567249(105)	5.574347(1017)	5.533116(6536)		
c /Å	7.654963(494)	7.578941(129)	7.623988 (1312)	7.868538(7702)		
Cell volume /$Å^3$	226.665(25)	221.052(7)	224.060(75)	227.350(615)		
$LaFeO_3$						
a /Å	5.570467(206)	5.568209(75)	5.569215(63)	5.568478(47)	5.565973 (30)	5.5621
b /Å	7.836199(312)	7.842201(129)	7.839102(100)	7.846164(81)	7.860953(88)	7.8516
c /Å	5.517392(208)	5.531999(87)	5.528562(65)	5.537154(52)	5.555669(43)	5.5520
Cell volume /$Å^3$	240.841(16)	241.566(6)	241.364(5)	241.925(4)	243.082(4)	242.46
R_{wp} **(%)**	2.2413	1.4776	1.8072	2.6825	2.9491	
R_{exp} **(%)**	2.2413	0.5731	0.4563	0.4478	0.4051	

The space group used for the refinement for $YbFeO_3$ and $LaFeO_3$ were *Pbnm* and *Pnma*, respectively.

7. $La_xY_{1-x}FeO_3$:

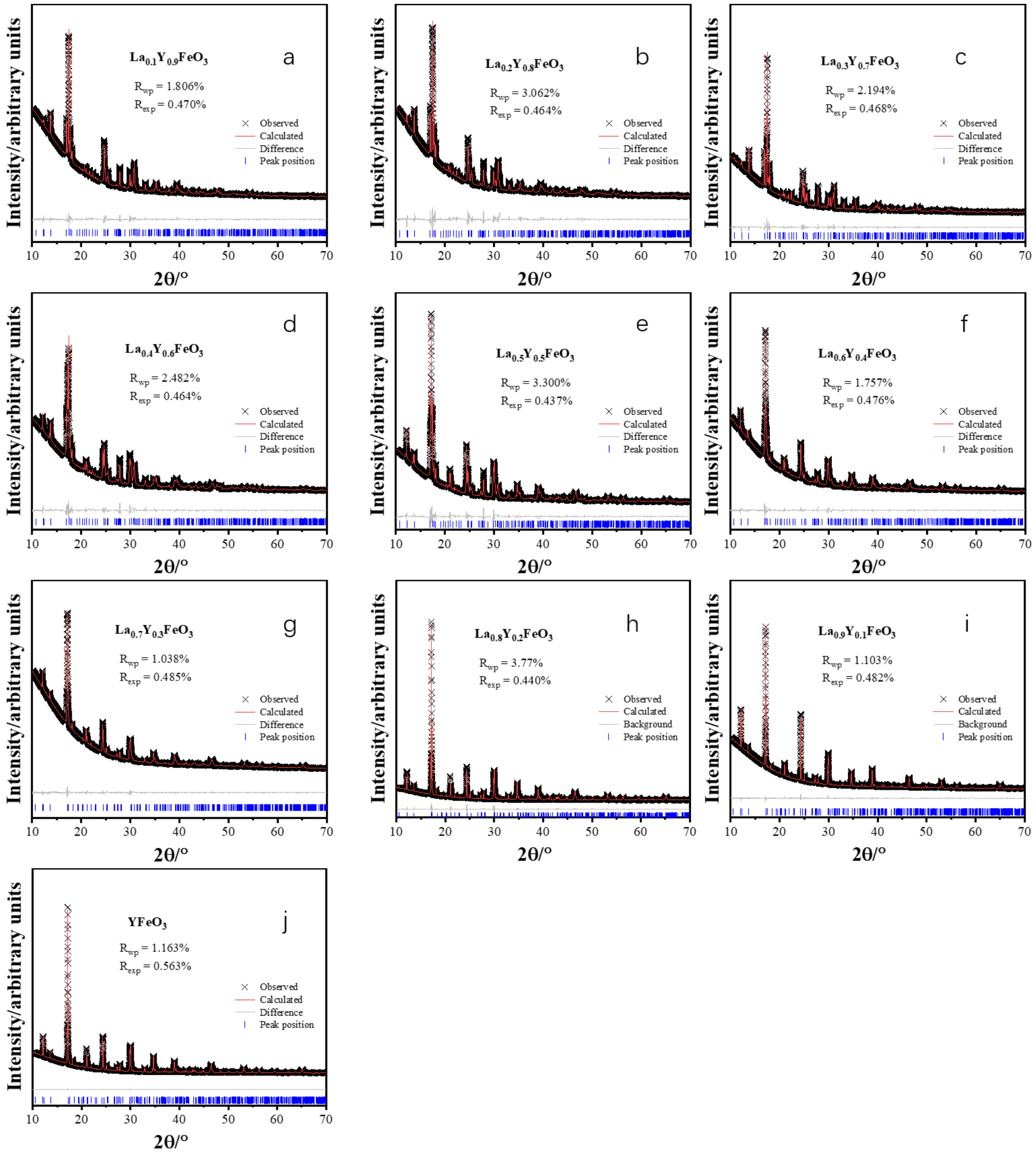

Figure S11 Rietveld fitted X-ray powder patterns (a,.., j) of $La_xY_{1-x}FeO_3$ (x = 0.1, 0.2, 0.3, .. 0.9, 1)

Table S7 Structural details obtained from Rietveld refinements against synchrotron X-ray diffraction for $La_xY_{1-x}FeO_3$

**Compounds**	$YFeO_3$(literature)	$YFeO_3$	$La_{0.1}Y_{0.9}FeO_3$	$La_{0.2}Y_{0.8}FeO_3$	$La_{0.3}Y_{0.7}FeO_3$	$La_{0.4}Y_{0.6}FeO_3$	$La_{0.5}Y_{0.5}FeO_3$
**Percent of phases**							
$LaFeO_3$ (%)		0	11.07	20.57	31.53	41.56	45.86
$YFeO_3$ (%)		100	88.93	79.43	68.47	58.44	54.14
**Lattice parameters**							
$YFeO_3$							
a /Å	5.2648	5.266287(31)	5.595345(126)	5.594358(214)	5.599697(79)	5.596651(175)	5.597080(244)
b/Å	5.5938	5.594936(34)	7.687151(201)	7.669171(106)	7.622166(106)	7.690052(315)	7.696839(455)
c /Å	7.6080	7.600250(48)	5.358110(114)	5.348381(72)	5.292945(72)	5.358328(179)	5.363757(251)
Cell volume/$Å^3$	224.06	223.938(2)	230.464(9)	229.467(16)	225.913(5)	230.615(14)	231.070(20)
$LaFeO_3$							
a /Å			5.588199(648)	5.588059(528)	5.593973(334)	5.577562(235)	5.577831(153)
b /Å			7.688715(633)	7.680773(614)	7.673064(472)	7.820138(312)	7.823929(206)
c /Å			5.424230(271)	5.412444(320)	5.349805(276)	5.497199(210)	5.506252(143)
Cell volume/$Å^3$			233.058(35)	232.305(32)	229.629(23)	239.773(17)	240.295(11)
**R_{wp} (%)**		3.7259	1.8060	3.0625	2.1946	2.4822	3.3006
**R_{exp} (%)**		0.4657	0.4701	0.4644	0.4680	0.4642	0.4370

compounds	$La_{0.6}Y_{0.4}FeO_3$	$La_{0.7}Y_{0.3}FeO_3$	$La_{0.8}Y_{0.2}FeO_3$	$La_{0.9}Y_{0.1}FeO_3$	$LaFeO_3$	$LaFeO_3$(simulated)
**Space group**					*Pnma*	*Pnma*
**Percent of phases**						
$LaFeO_3$ (%)	49.90	65.60	68.90	96.08	0	
$YFeO_3$ (%)	50.10	44.39	31.10	3.92	100	
**Lattice parameters**						
$YFeO_3$						
a /Å	5.576449(920)	5.524078(939)	5.505013(185)	5.392775(1130)		
b/Å	7.758592(1231)	7.818060(1211)	7.832653(375)	5.522649(1760)		
c /Å	5.398311(754)	5.480778(666)	5.539895(260)	7.900650(2051)		
Cell volume/Å^3	233.560(63)	236.701(62)	238.874(18)	235.300(109)		
$LaFeO_3$						
a/Å	5.576407(116)	5.576660(98)	5.574023(44)	5.572331(261)	5.565973(30)	5.5621
b/Å	7.823549(160)	7.821132(147)	7.830039(71)	7.845341(361)	7.860953(88)	7.8516
c/Å	5.512612(121)	5.515193(148)	5.521292(60)	5.534194(256)	5.555669(43)	5.5520
Cell volume /Å^3	240.500(9)	240.549(9)	240.976(4)	241.937(19)	243.082(4)	242.46
**R_{wp} (%)**	1.7573	1.0388	3.7710	0.8611	2.9491	
**R_{exp} (%)**	0.4764	0.4854	0.4403	0.4763	0.4051	

The literature used to compare with the obtained $YFeO_3$ and $LaFeO_3$ is same as the one mentioned in the paper. The space groups used for the refinement for $YFeO_3$ and $LaFeO_3$ were *Pbnm* and *Pnma*, respectively.

S3: Fitted Raman spectra

1. $La_xNd_{1-x}FeO_3$:

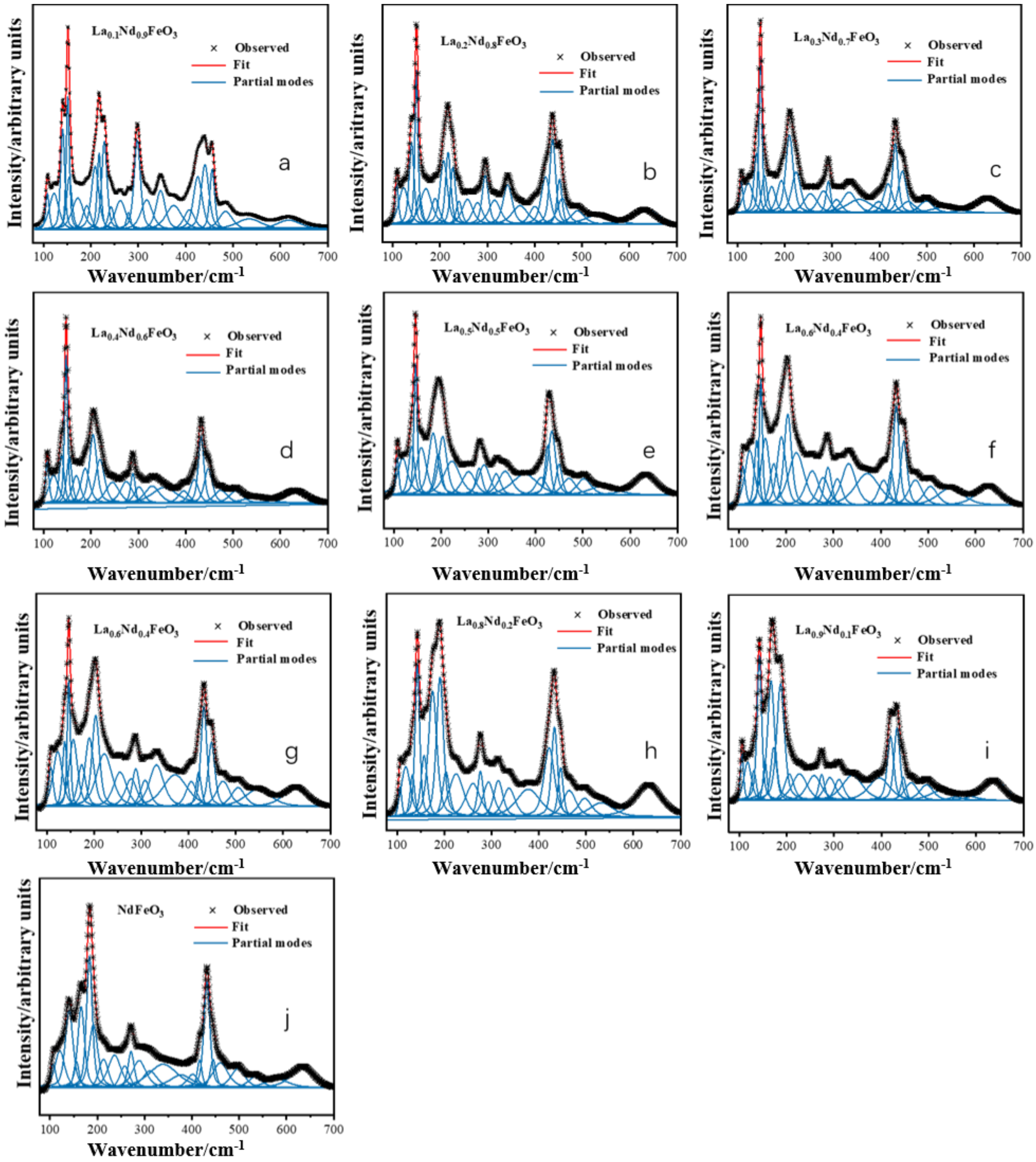

Figure S12 Fitted Raman spectra (a, …, j) of $La_xNd_{1-x}FeO_3$ (x = 0.1, 0.2, 0.3, …, 0.9, 1)

Raman spectra analysis for $La_xNd_{1-x}FeO_3$ can be found in the main text.

2. $La_xSm_{1-x}FeO_3$:

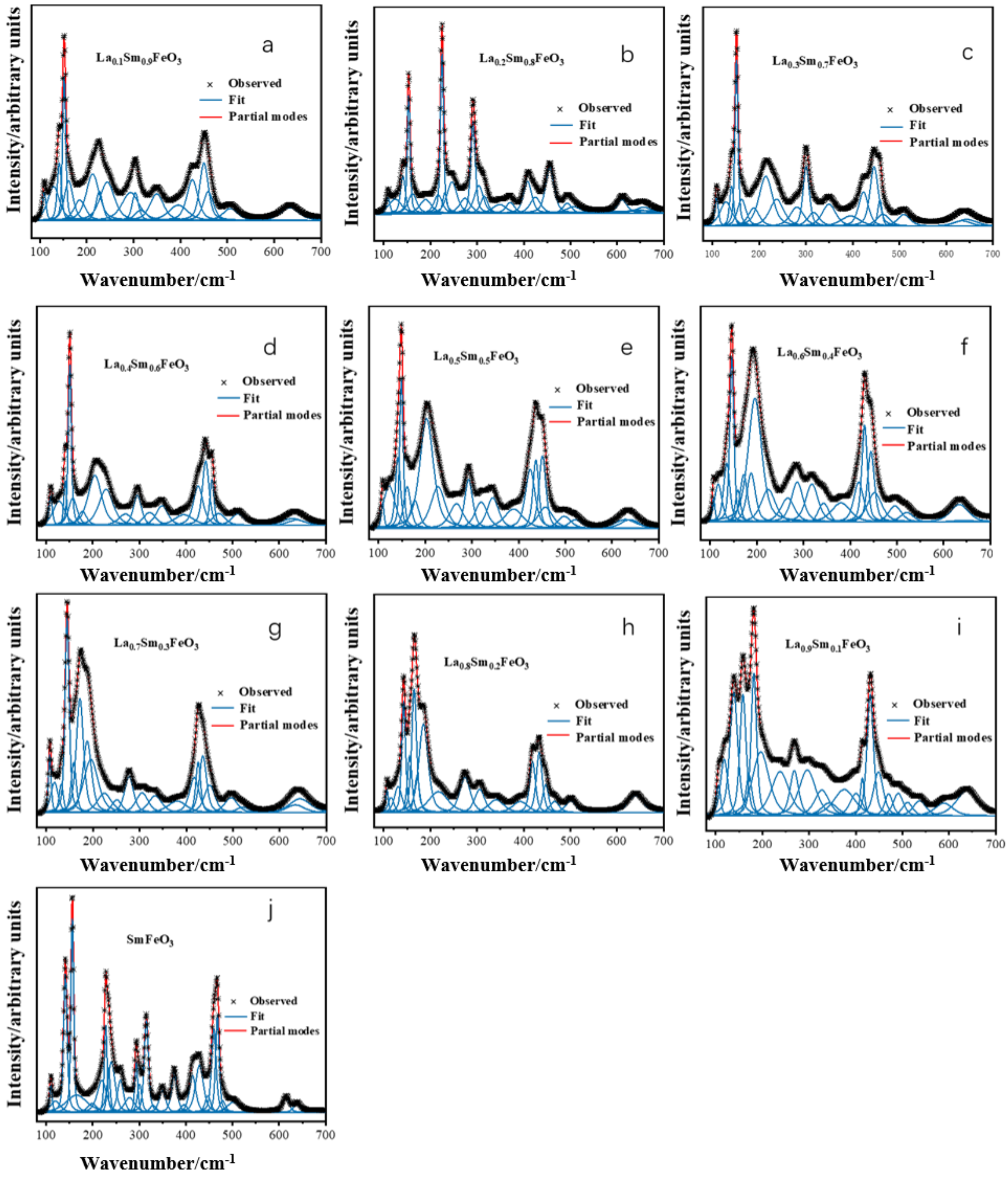

Figure S13 Fitted Raman spectra (a, …, j) of $La_xSm_{1-x}FeO_3$ (x = 0.1, 0.2, 0.3, …, 0.9, 1)

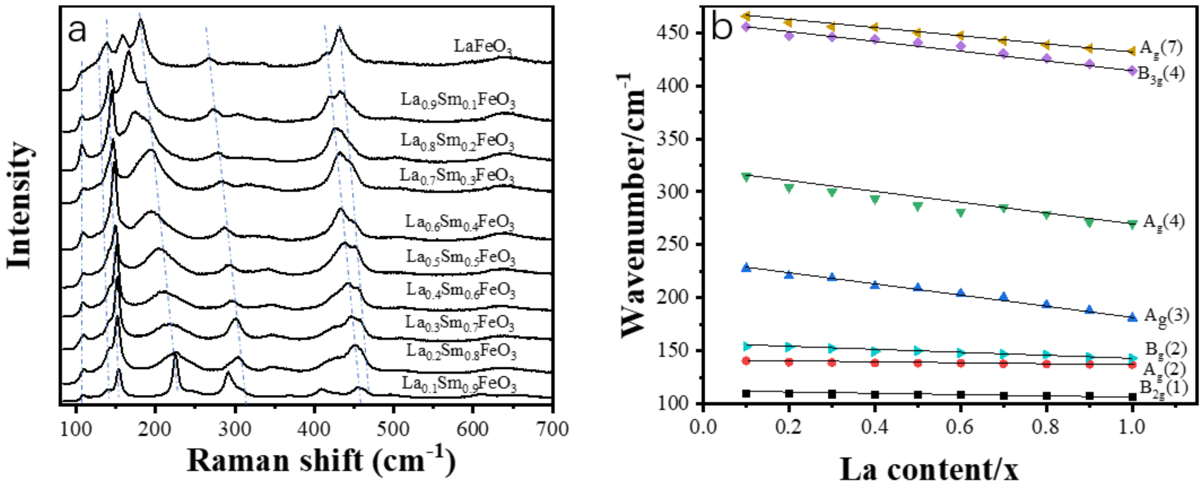

Figure S14 a, b) Raman spectra and wavenumber shifts in phonon modes for $La_xSm_{1-x}FeO_3$ series (x = 0.1, 0.2, …, 1)

Table S8 Assigned phonon modes and atomic motions of $SmFeO_3$

Symmetry	Position/cm^{-1}	Atomic motion
$\boldsymbol{A_g}$**(2)**	141	A(z) out-of-phase
$\boldsymbol{A_g}$**(3)**	217	FeO_6 rotation, in-phase
$\boldsymbol{A_g}$**(4)**	298.1	O(1) x-z plane
$\boldsymbol{B_{3g}}$**(4)**	453.6	FeO_6 scissor-like bending, out-of-phase
$\boldsymbol{A_g}$**(7)**	464.7	FeO_6 scissor-like bending
$\boldsymbol{B_{2g}}$**(1)**	111.3	A(z), in-phase in x-z, out-of-phase in y
$\boldsymbol{B_{2g}}$**(2)**	154.6	A(x), out-of-phase

.

3. $La_xGd_{1-x}FeO_3$:

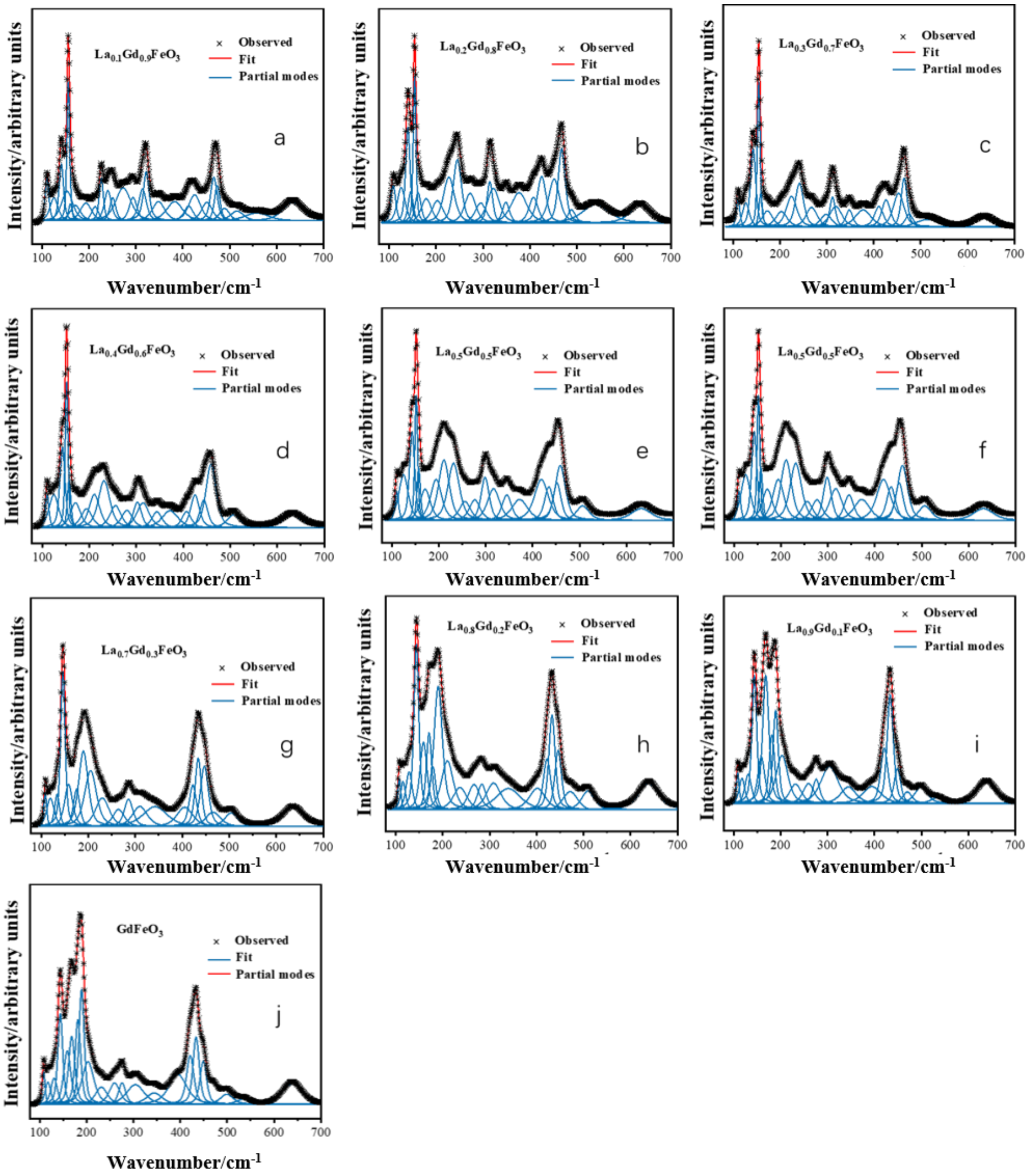

Figure S15 Fitted Raman spectra (a, …, j) of $La_xGd_{1-x}FeO_3$ (x = 0.1, 0.2, 0.3, …, 0.9, 1)

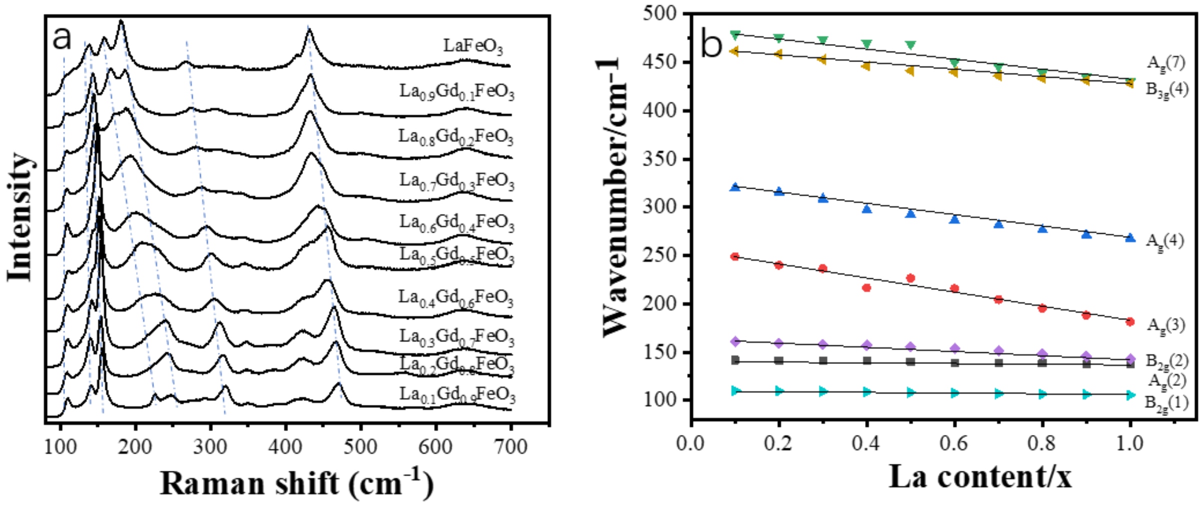

Figure S16 a, b) Raman spectra and wavenumber shifts in phonon modes for $La_xGd_{1-x}FeO_3$ series (x = 0.1, 0.2, …, 1)

Table S9 Assigned phonon modes and atomic motions of $GdFeO_3$

Symmetry	Position/cm^{-1}	Atomic motion
A_g(2)	141	A(z) out-of-phase
A_g(3)	219	FeO_6 rotation, in-phase
A_g(4)	298	O(1) x-z plane
B_{3g}(4)	454.3	FeO_6 scissor-like bending, out-of-phase
A_g(7)	465.5	FeO_6 scissor-like bending
B_{2g}(1)	112	A(z), in-phase in x-z, out-of-phase in y
B_{2g}(2)	154	A(x), out-of-phase

The Raman spectra (Figures S13 and S15) of $La_xRE_{1-x}FeO_3$ (*RE* = Gd and Sm) samples exhibit typical features of an orthorhombic perovskite. The band positions have an obvious evolution with composition with a continuous shift to lower frequencies when the La content increases, consistent with the formation of a homogeneous solid solution. The observed phonon modes were assigned to specific vibrational symmetries using data from a previous systematic study of *RE*FeO_3 that included the end members $LaFeO_3$ and *RE*FeO_3. The assigned phonon modes are listed in Tables S8 and S9. Blue shifts of O−Fe−O bending (~441 cm^{-1}) modes indicate the distortion of the FeO_6 octahedra while different rare earth ions take up the A site with different content.

The deconvolution of Raman spectra for $La_xHo_{1-x}FeO_3$, $La_xEr_{1-x}FeO_3$, and $La_xYb_{1-x}FeO_3$ samples has not been performed because these are consistent with two separate but rather similar phases. The wavenumbers of the photon modes of the two phases are close to each other due to the similarity of the intrinsic properties of these rare earth elements. Thus, it is impossible to precisely identity the accurate mode positions for each phase to perform the deconvolution.

S4: Magnetism analysis

1. $La_xSm_{1-x}FeO_3$:

The temperature dependence of the magnetisation of $SmFeO_3$ measured in zero-field cooled (ZFC) and field-cooled cooling (FCC) modes between 2 and 300 K is shown in Figure S17(a). The data are in good agreement with previous studies of single crystal $SmFeO_3$.[3] The temperature range shown here is well below the long-range antiferromagnetic (AFM) ordering temperature of the Fe sublattice, which is reported at a Néel temperature $T_N \approx 670$ K.[1, 2, 4, 5] A spin reorientation of the Fe moments is reported to occur at 480 K[5, 6]， a much higher a temperature than that observed in some other rare-earth orthoferrites. As a result, there is no significant hysteresis (cf. Nd or Er orthoferrite) between the ZFC and FCC curves. The downturn at lower temperatures is due to an alignment of the Sm moments in the exchange field of the Fe sublattice. A minimum at ~4 K is close to the compensation point reported in single crystals. [3, 6]

The temperature dependence of the magnetisation of $La_{0.5}Sm_{0.5}FeO_3$ is shown in Figure S17(b). The magnitude of the signal is unchanged, suggesting that at high temperature the magnitude of the signal is largely determined by the canting in the Fe sublattice. The hysteresis and downturn in the magnetisation are shifted to lower temperature and the minimum at 4 K is completely removed indicating that the substitution of La for Sm disrupts any alignment within the Sm sublattice.

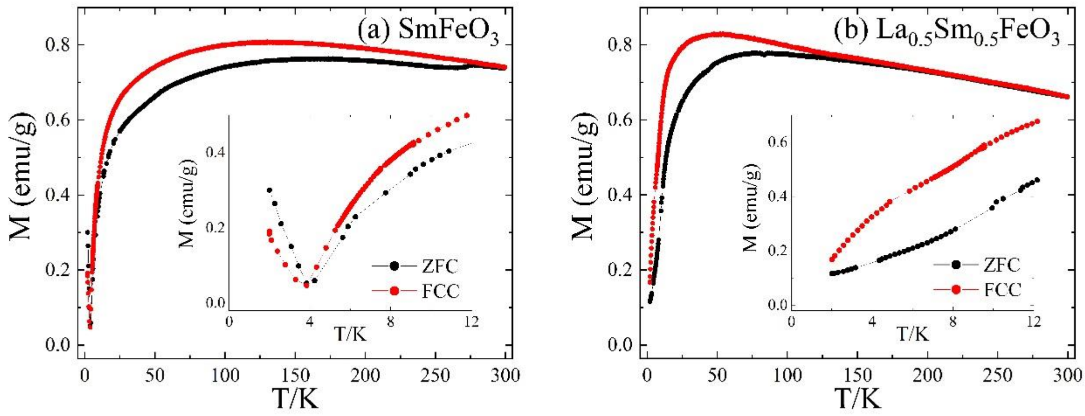

Figure S17: Temperature dependence of the magnetisation (ZFC and FCC) of (a) $SmFeO_3$ and (b) $La_{0.5}Sm_{0.5}FeO_3$ at 1 kOe from 2 to 300 K. The insets show the minimum in (a) $SmFeO_3$ at 4 K which is removed in (b) $La_{0.5}Sm_{0.5}FeO_3$.

2. $La_xGd_{1-x}FeO_3$:

The temperature dependence of the magnetisation of $GdFeO_3$ is shown in Figure S18(a). The Fe sublattice is reported to order at ~655 K.[1, 4, 5] The Gd moments align parallel to the Fe moments leading to a large magnetisation at low temperature.[7] There is no spontaneous spin reorientation down to low temperature in $GdFeO_3$ although it is reported that a field induced spin rotation occurs in magnetic fields of 76 kOe at 293 K and 81 kOe at 77 K. [8] An ordering of the Gd moments at $T_N \approx 1.47$ K[9] means this field induced spin reorientation is at a smaller field of 7 kOe at 2 K. The magnetisation versus temperature of the of $La_{0.5}Gd_{0.5}FeO_3$ is shown in Figure S18(b). At low temperature the signal is reduced as expected, die to the lower Gd content. At higher temperature the magnetisation becomes temperature independent, suggesting that the Gd is incorporated into the structure, rather than forming a phase separated mixture of $GdFeO_3$ diluted by $LaFeO_3$,

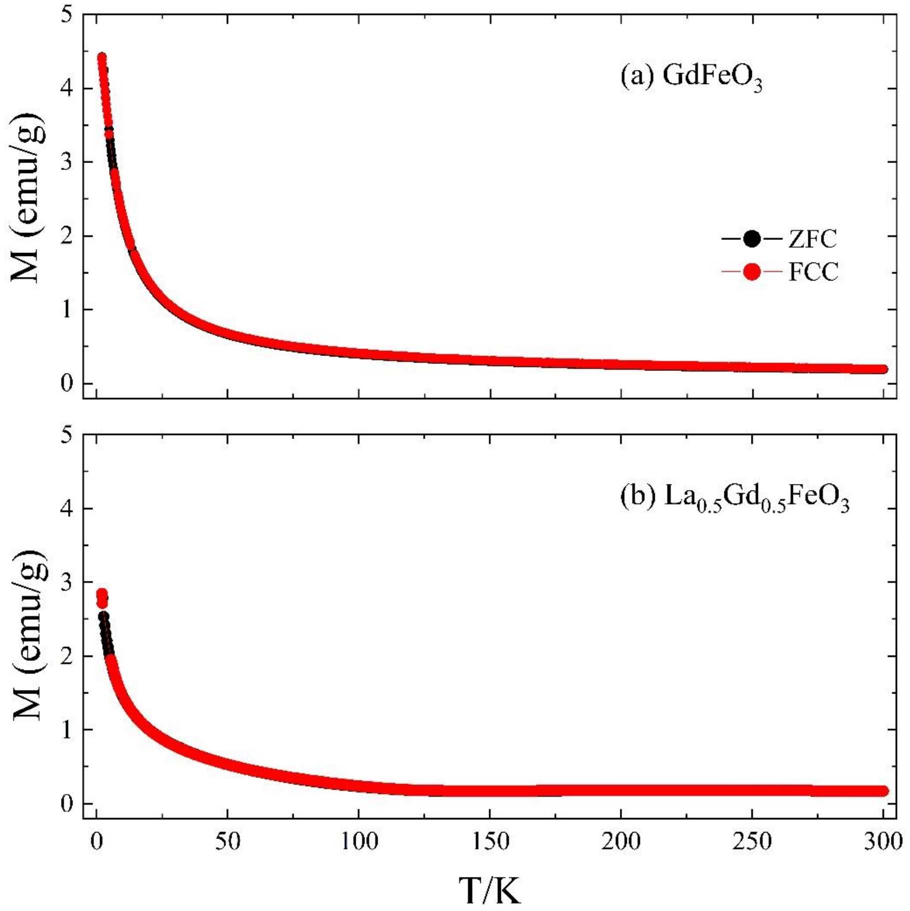

Figure S18: Temperature dependence of the magnetisation (ZFC and FCC) of (a) $GdFeO_3$ and (b) $La_{0.5}Gd_{0.5}FeO_3$ at 1 kOe from 2 to 300 K.

3. $La_xHo_{1-x}FeO_3$:

Figure S19 shows the temperature dependence of the magnetisation of $HoFeO_3$ and $La_{0.5}Ho_{0.5}FeO_3$. The Fe sublattice orders at ~645 K.[1, 4, 5] For $HoFeO_3$, a spin reorientation leading to a step in the magnetisation is observed between 40 (T_{SR1}) and 70 K (T_{SR2}). Similar features at comparable temperatures are reported for both polycrystalline and single crystal $HoFeO_3$[2, 5, 10]. Compared with $NdFeO_3$, the spin reorientation in $HoFeO_3$ occurs over a narrow temperature window of just 30 K. Moreover, there is very little hysteresis between the ZFC and FCC curves around T_{SR1} and T_{SR2} in $HoFeO_3$. The signal at low temperatures is significantly larger than for other rare earth orthoferrites studied here, suggesting the field polarisation of the large moment on the Ho^{3+} ions dominates over any signal due to the reorientation of the Fe sublattice. [11] For $La_{0.5}Ho_{0.5}FeO_3$, the magnetisation at low temperatures is around half that seen in $HoFeO_3$. There is still a degree of hysteresis between the ZFC and FCC at lower temperatures, and the step in M(T) associated the spin reorientation is still visible over the same temperature range. These data are consistent with a phase separated mixture of $HoFeO_3$ diluted by $LaFeO_3$, although there may be some incorporation of a small number of nonmagnetic La ions into the Ho sites in the $La_{0.5}Ho_{0.5}FeO_3$ material.

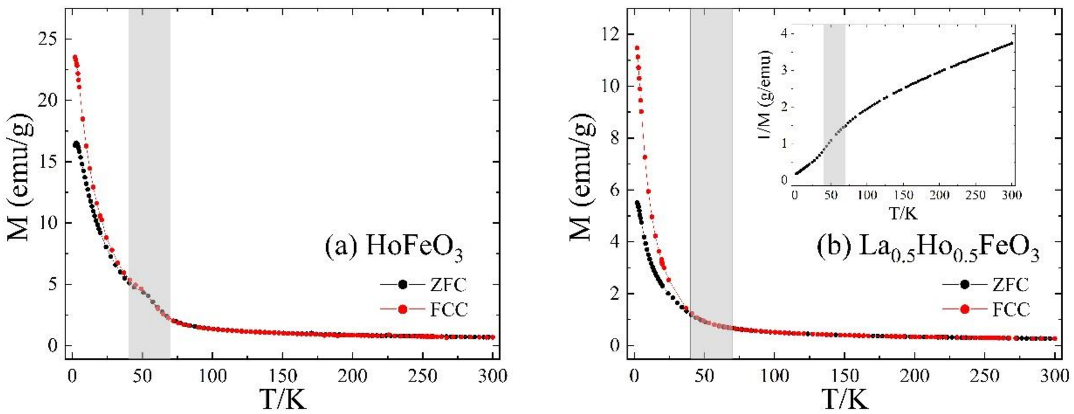

Figure S19: Temperature dependence of the magnetisation (ZFC and FCC) of (a) $HoFeO_3$ and (b) $La_{0.5}Ho_{0.5}FeO_3$ in 1 kOe from 2 to 300 K. The inset in (b) shows the inverse of the ZFC magnetisation versus temperature, to more clearly highlight the step at the spin reorientation.

4. $La_xYb_{1-x}FeO_3$:

For $YbFeO_3$, the Néel temperature of the Fe sublattice is T_N = 630 K, similar to the other rare-earth orthoferrites.[1, 4, 5] However, in $YbFeO_3$, a spin reorientation of the Fe moments is reported to occur at much lower temperatures between 6 and 8 K. This spin reorientation is clearly visible in Fig S20(a) as the onset of hysteresis between the ZFC and FCC magnetisation curves at ~ 7 K. The magnitude of the signal and the temperature dependence of the magnetisation are consistent with data collected on single crystal $YbFeO_3$.[8, 12]

The magnetisation versus temperature data for $La_{0.5}Yb_{0.5}FeO_3$ are qualitatively very similar. The magnitude of the magnetisation is approximately half that of $YbFeO_3$ at both 300 K and at low temperature. The onset of hysteresis between the ZFC and FCC data also occurs at ~ 7 K, which indicates that the spin reorientation transition temperature is unchanged. These data are consistent with the $La_{0.5}Yb_{0.5}FeO_3$ powders being a mixture of $LaFeO_3$ and $YbFeO_3$, although once again, a cross substitution of some La onto the Yb sites in $YbFeO_3$ and vice-versa cannot be ruled out. These data are in good agreement with the XRD and EDS results.

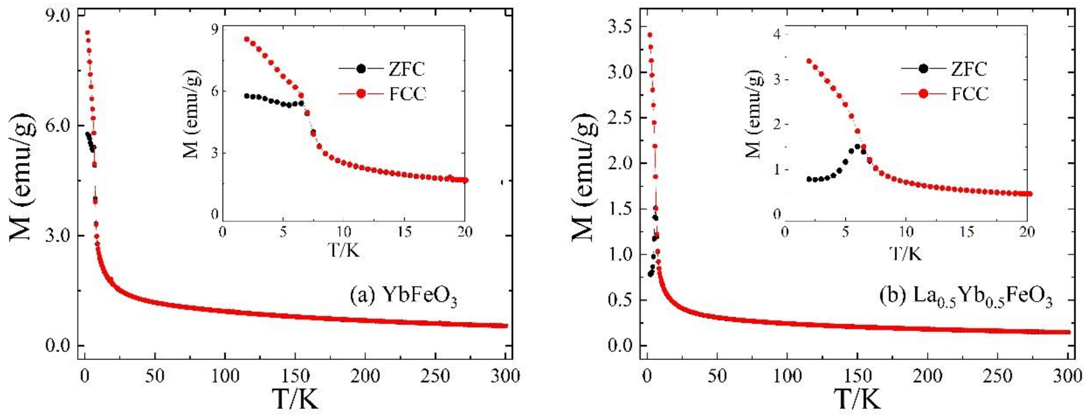

Figure S20: Temperature dependence of the magnetisation (ZFC and FCC) of (a) $YbFeO_3$, and (b) $La_{0.5}Yb_{0.5}FeO_3$ in 1 kOe from 2 to 300 K, respectively. The insets show the spin reorientation transition that occurs at ~ 7 K and results in hysteresis between the ZFC and FCC data.

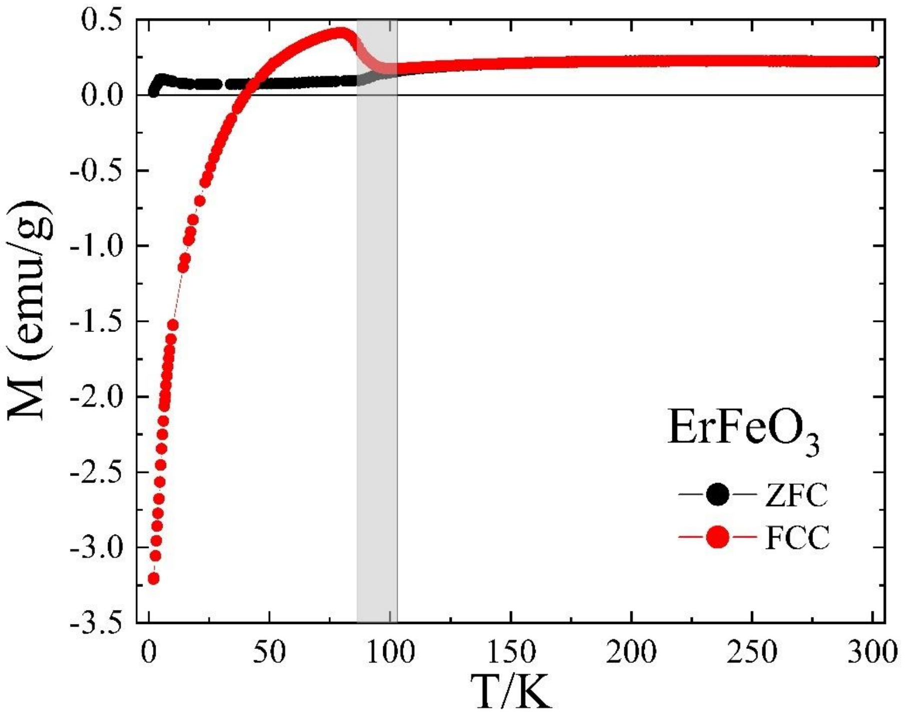

Figure S21: Temperature dependence of the magnetisation (ZFC and FCC) of $ErFeO_3$ in 100 Oe from 2 to 300 K. The grey shaded area indicates the temperature region over which the spin reorientation takes place. There is compensation point at ~40 K.

S5: Scanning electron microscopy images

1. $La_xNd_{1-x}FeO_3$

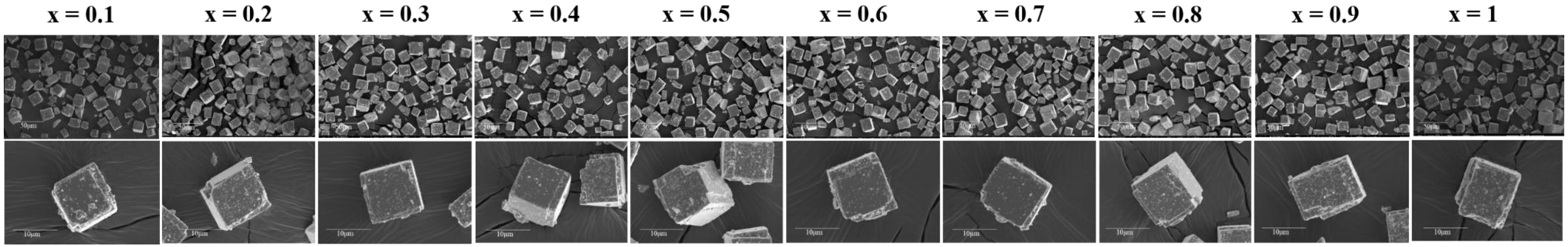

Figure S22: Electron micrographs of $La_xNd_{1-x}FeO_3$ (x = 0.1, 0.2, 0.3, …, 0.9, 1)

2. $La_xSm_{1-x}FeO_3$

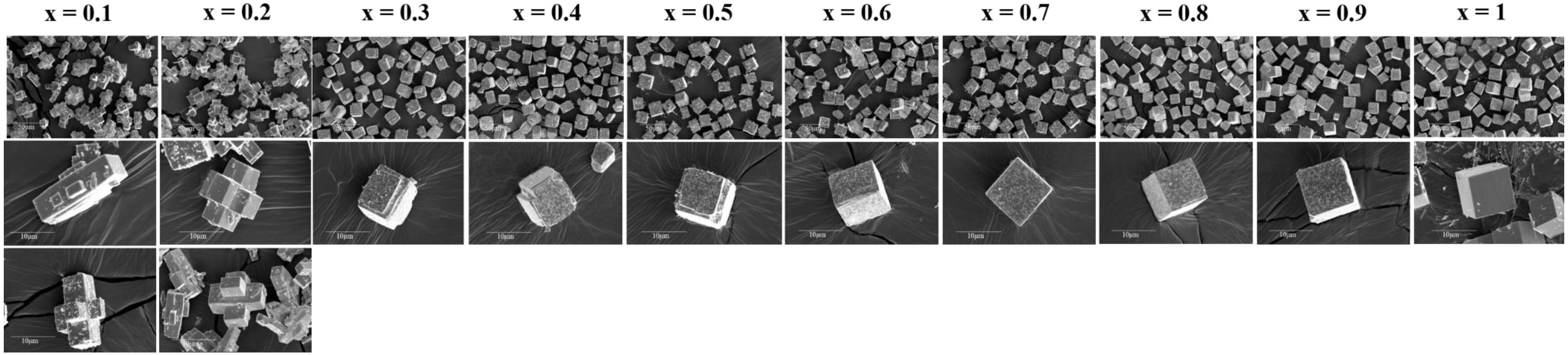

Figure S23: Electron micrographs of $La_xSm_{1-x}FeO_3$ (x = 0.1, 0.2, 0.3, …, 0.9, 1)

3. $La_xGd_{1-x}FeO_3$

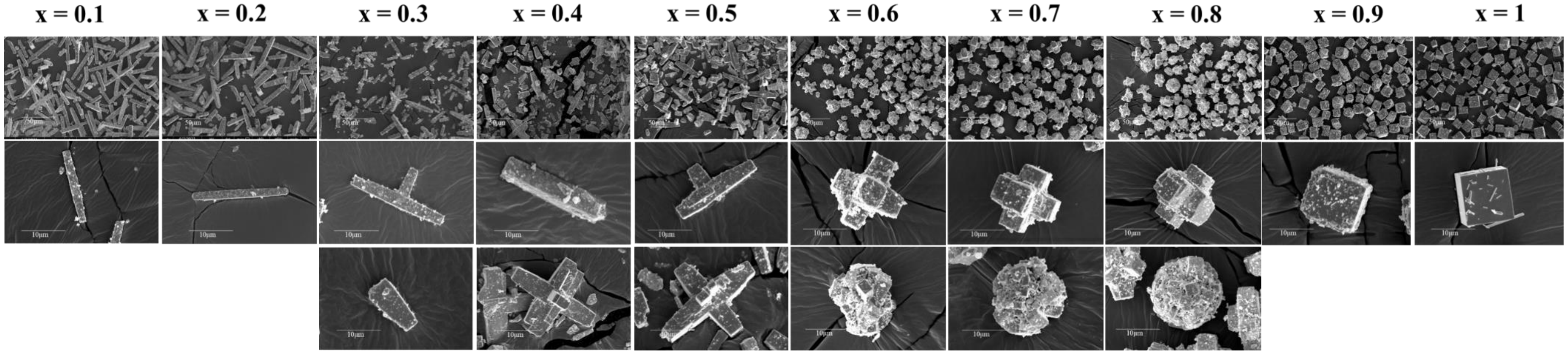

Figure S24: Electron micrographs of $La_xGd_{1-x}FeO_3$ (x = 0.1, 0.2, 0.3, …, 0.9, 1)

4. $La_xHo_{1-x}FeO_3$

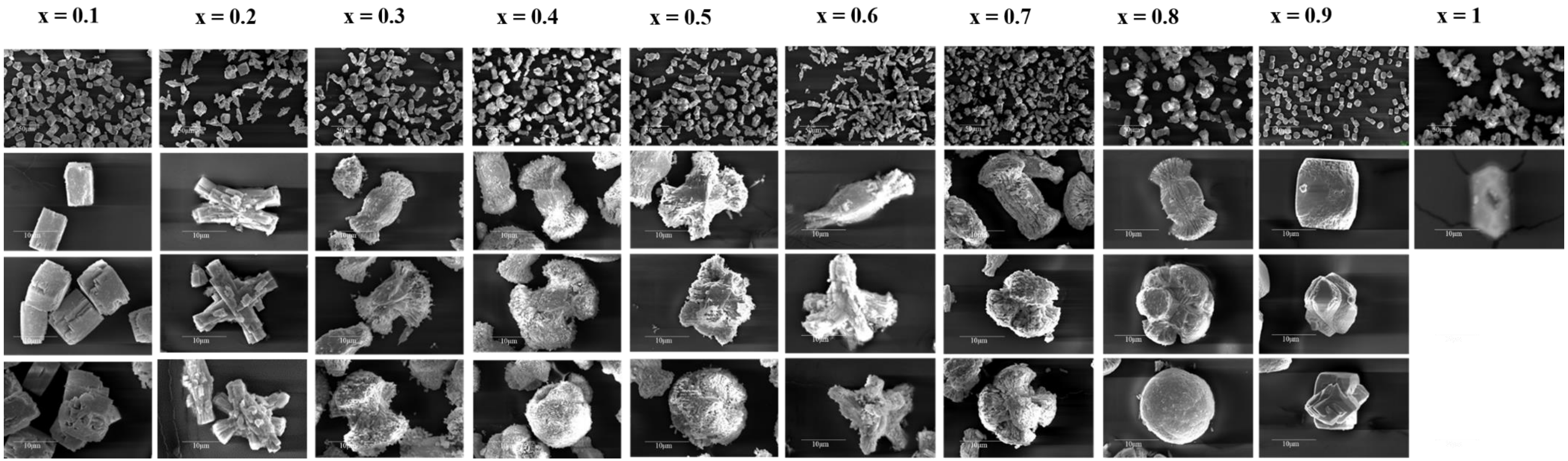

Figure S25: Electron micrographs of $La_xHo_{1-x}FeO_3$ (x = 0.1, 0.2, 0.3, …, 0.9, 1)

5. $La_xEr_{1-x}FeO_3$

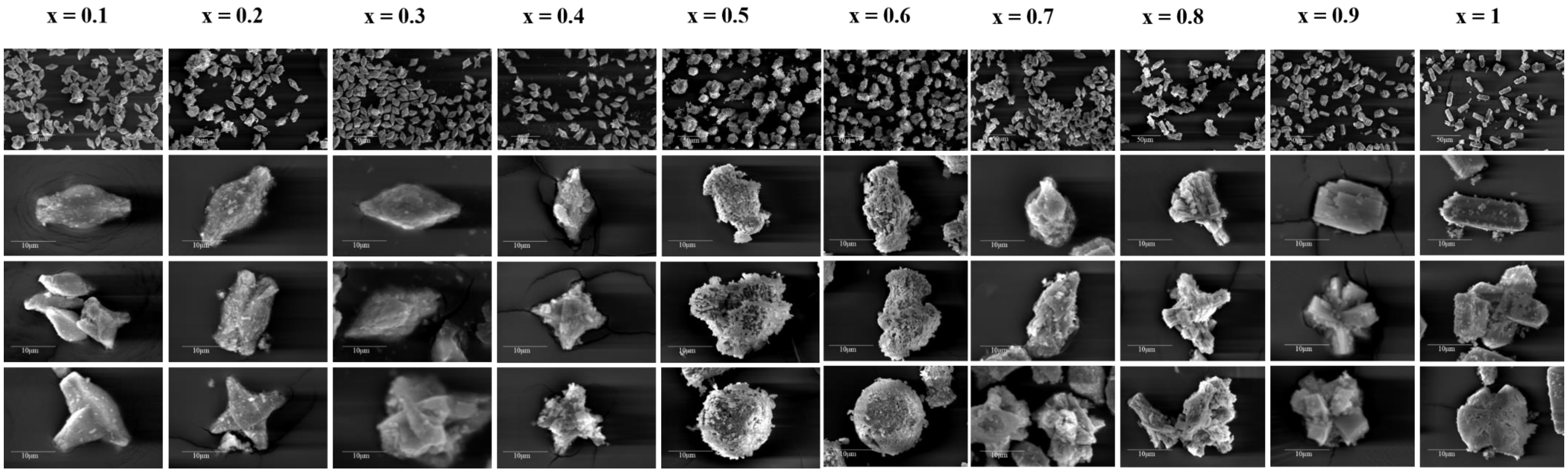

Figure S26: Electron micrographs of $La_xEr_{1-x}FeO_3$ (x = 0.1, 0.2, 0.3, …, 0.9, 1)

6. $La_xYb_{1-x}FeO_3$

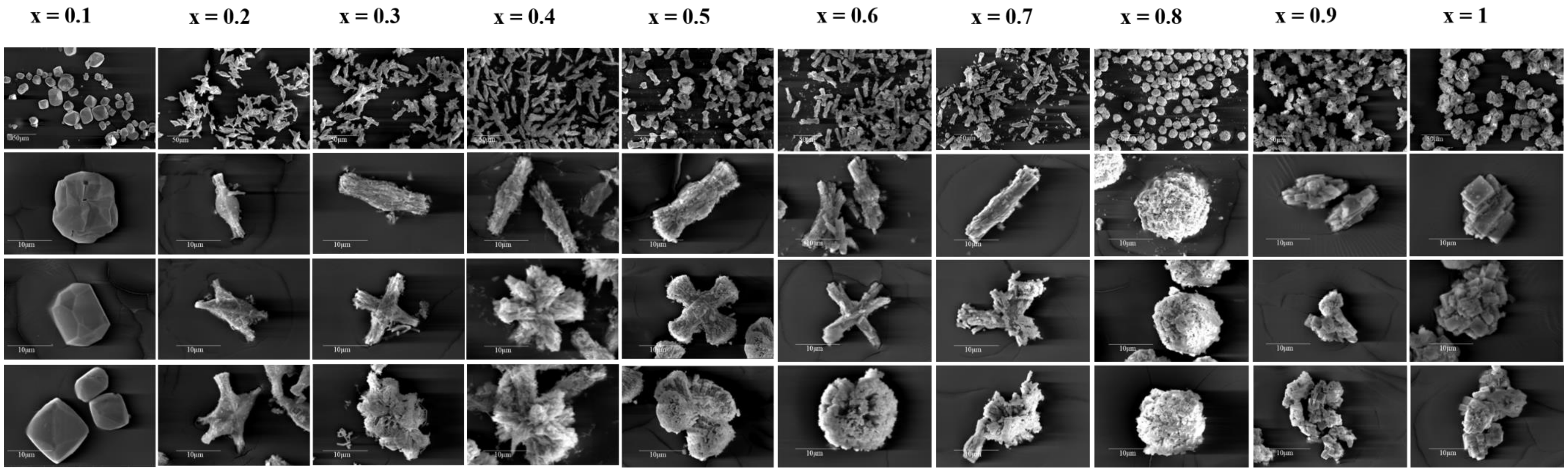

Figure S27: Electron micrographs of $La_xYb_{1-x}FeO_3$ (x = 0.1, 0.2, 0.3, …, 0.9, 1)

7. $La_xY_{1-x}FeO_3$

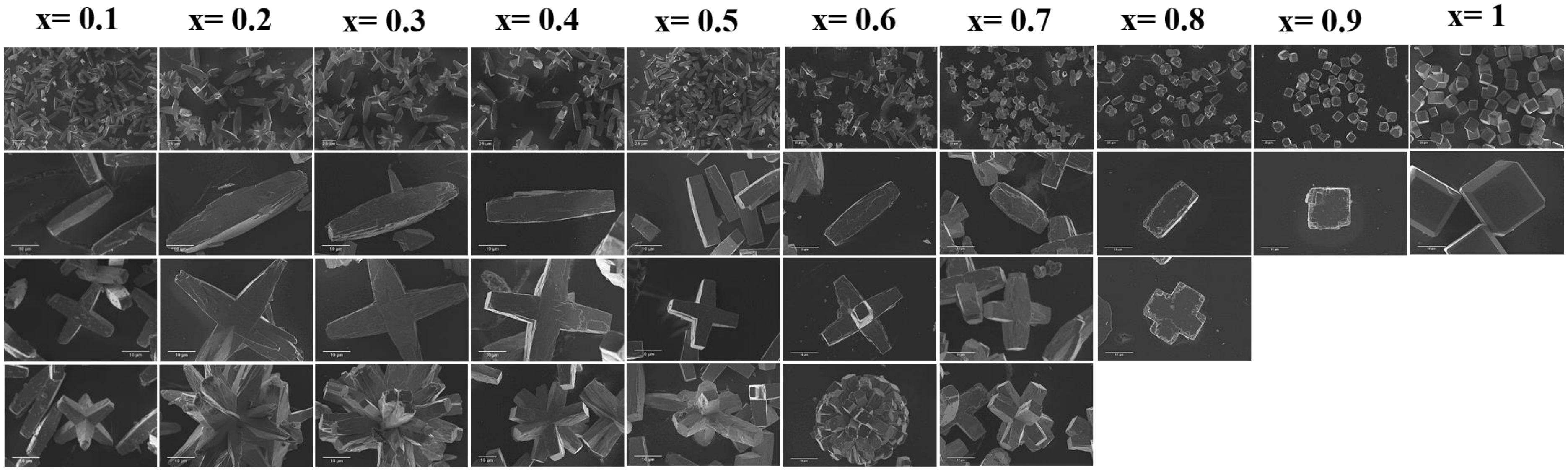

Figure S28: Electron micrographs of $La_xY_{1-x}FeO_3$ (x = 0.1, 0.2, 0.3, …, 0.9, 1)

The SEM images of the $La_xNd_{1-x}FeO_3$ samples are shown in Figure S22. All the samples present cubic shape with a relatively small size, which is estimated in the range of 20μm to 25 μm. Over the whole substitution range from 0 to 1, the morphologies remain cubic shape. The $La_xSm_{1-x}FeO_3$ and $La_xGd_{1-x}FeO_3$ all have a morphology evolution with increasing La^{3+} amount.

For the mixed-phases samples, all the samples have relatively small sizes, which is estimated to be in the range of 20μm to 30μm (Figures S24, 25, 26,27). Their crystals are not like the solid solutions which are mostly cubic shapes but irregular shapes. For $La_x\mathit{RE}_{1-x}FeO_3$ (*RE* = Ho, Er, Yb) samples, taking $La_xYb_{1-x}FeO_3$ as an example, with increase of La^{3+}, the materials reveals substantial changes in crystal morphology. When the amount of La^{3+} is 10%, the shape of the ample looks like a spindle, when the substituent amount increases, the particles of the sample grow more arms from the middle of the bar in different dimensions, first in the “z” axis, i.e., in the direction perpendicular to the initial growth directions, then trending towards spheroids composed of multiple rods. Finally, the morphology evolution ends with a cubic shape. It should be noted that all of the samples are constructed by small bricks of crystals.

The $La_xYb_{1-x}FeO_3$ samples also show a strong evolution of crystal morphology with increase of Yb, but the materials are more regular than other mixed-phase samples. This morphological evolution happens from an elongation of the original cube shape seen in pure $LaFeO_3$ system to a maximum length of ~25 µm (up from core particle size of ~10-15 µm), followed by additional perpendicular growths at the particle centre. These additional growths appear to always occur in the same plane as the first axial elongation, and reach the same length maximum (full particle length in direction of primary and secondary axial growth is equal at ~25 µm). At higher concentrations of Yb the particles continue to grow arms from a central core, in the direction perpendicular to the initial growth directions (Fig 15b – c) and then trending towards spheroids composed of multiple rods. Morphology changes are common in hydrothermal synthesis, with morphology changes reported for complexing/templating agents[13, 14] or changes in pH, however, to the authors' knowledge, there are no reported cases of the a doped bi-phasic material undergoing crystal growth in a similar nature to that seen here.

S6: SEM-EDS maps

1. $La_xGd_{1-x}FeO_3$

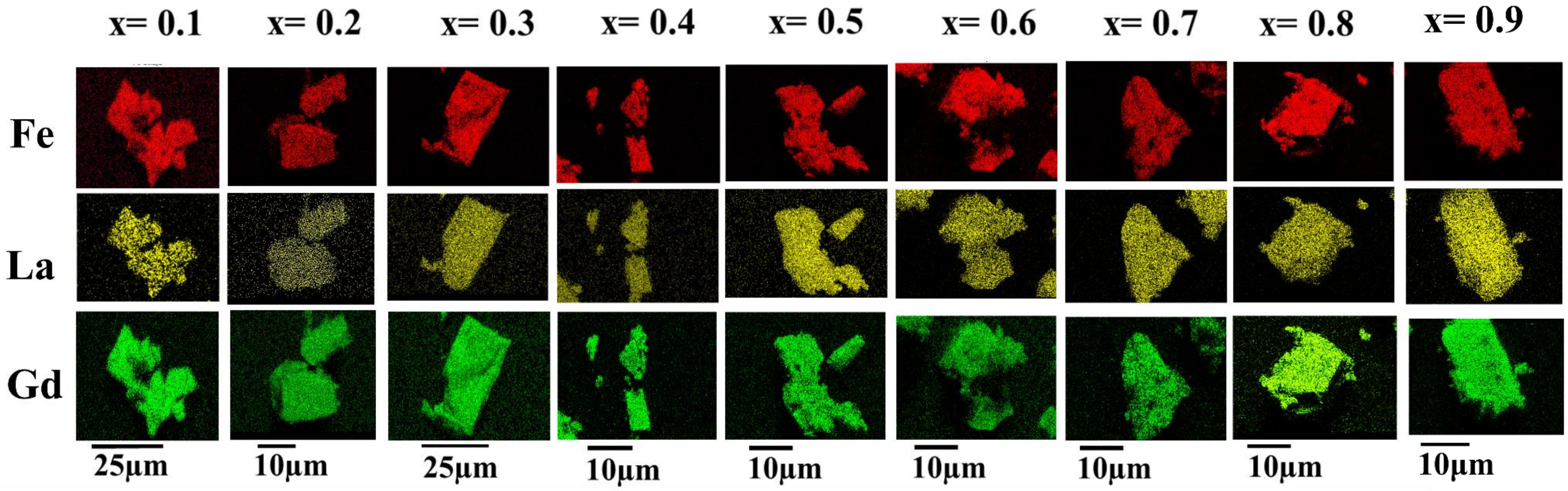

Figure S29: EDS maps of $La_xGd_{1-x}FeO_3$ (x = 0.1, 0.2, 0.3, …, 0.9)

2. $La_xSm_{1-x}FeO_3$

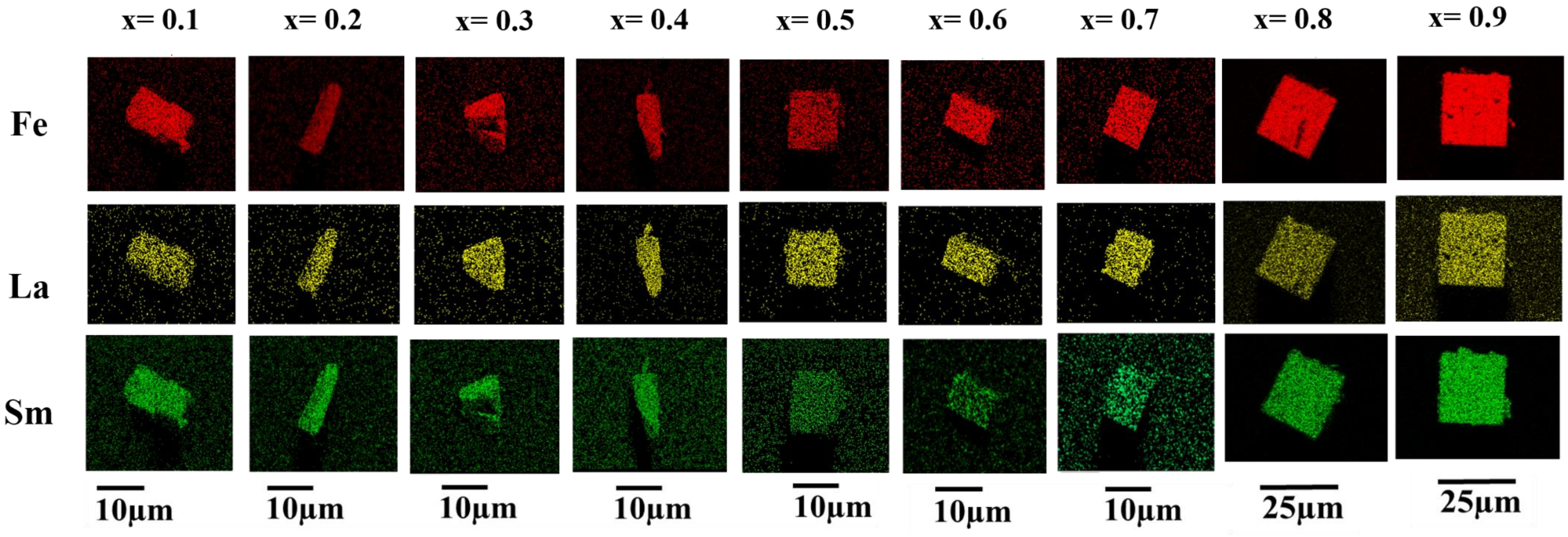

Figure S30: EDS maps of $La_xSm_{1-x}FeO_3$ (x = 0.1, 0.2, 0.3, …, 0.9)

3. $La_xHo_{1-x}FeO_3$

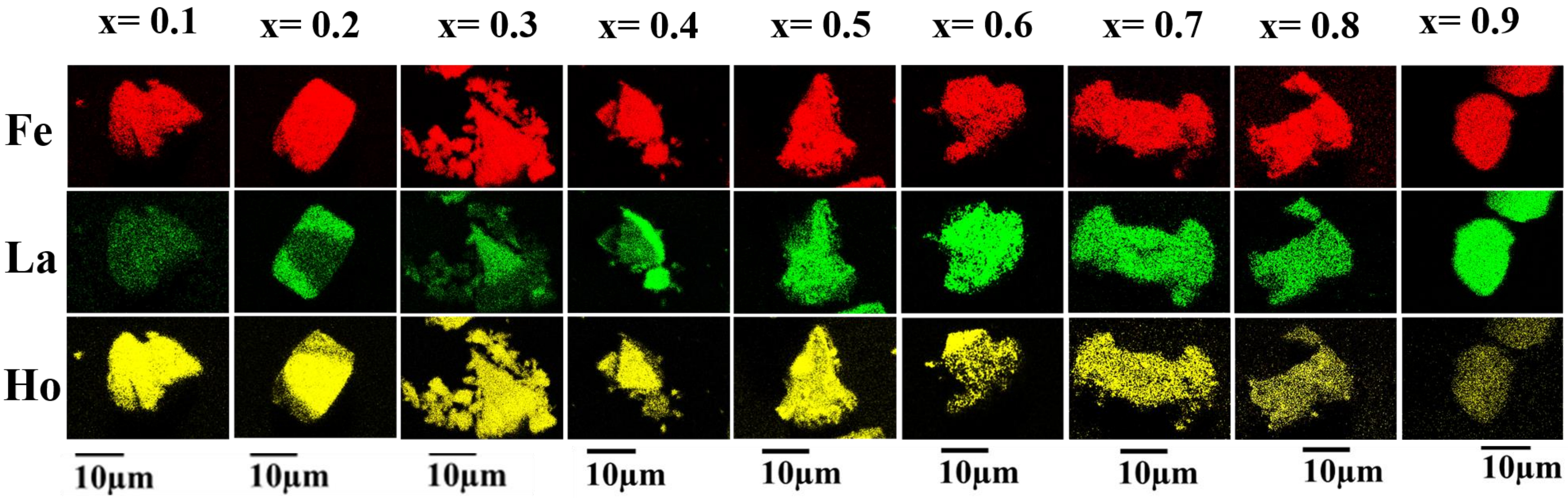

Figure S31: EDS maps of $La_xHo_{1-x}FeO_3$ (x = 0.1, 0.2, 0.3, …, 0.9)

4. $La_xYb_{1-x}FeO_3$

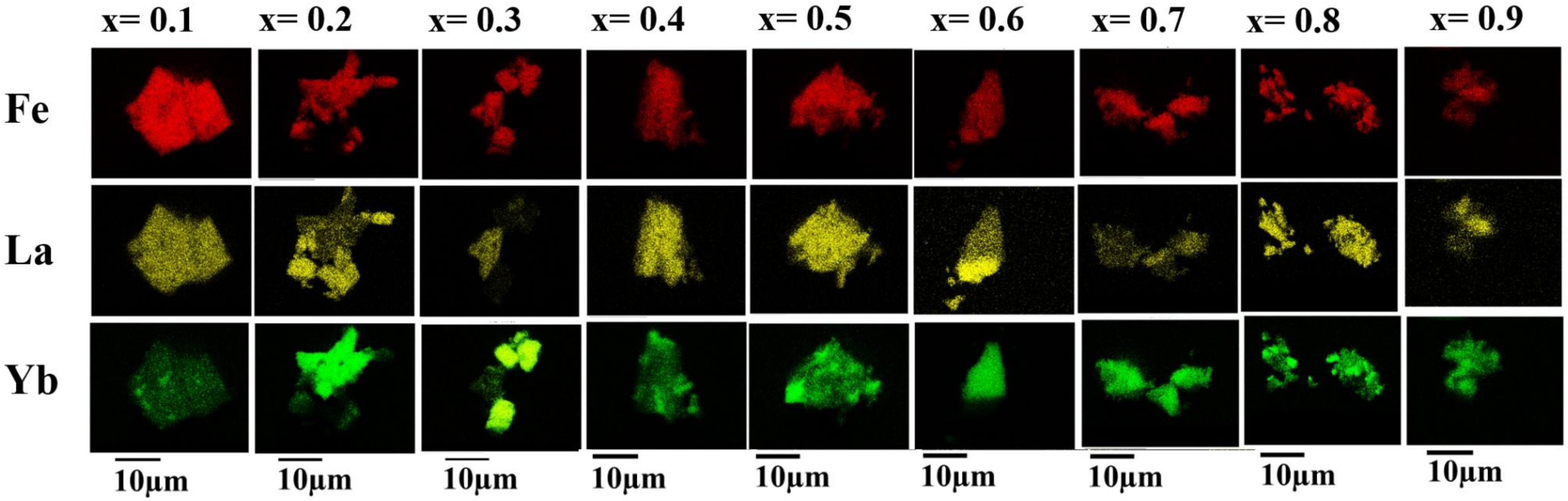

Figure S32: EDS maps of $La_xYb_{1-x}FeO_3$ (x = 0.1, 0.2, 0.3, …, 0.9)

Table S10: EDS analysis results for the elemental composition of $La_xNd_{1-x}FeO_3$ (normalised to Fe)

Target material	Target La:Nd:Fe:O ratio	La	Nd	Fe
$La_{0.9}Nd_{0.1}FeO_3$	0.9:0.1:1:3	0.98	0.08	1
$La_{0.8}Nd0_{.2}FeO_3$	0.8:0.2:1:3	0.69	0.12	1
$La_{0.7}Nd_{0.3}FeO_3$	0.7:0.3:1:3	0.69	0.23	1
$La_{0.6}Nd_{0.4}FeO_3$	0.6:0.4:1:3	0.44	0.31	1
$La_{0.5}Nd_{0.5}FeO_3$	0.5:0.5:1:3	0.88	0.97	1
$La_{0.4}Nd_{0.6}FeO_3$	0.4:0.6:1:3	0.37	0.88	1
$La_{0.3}Nd_{0.7}FeO_3$	0.3:0.7:1:3	0.13	0.91	1
$La_{0.2}Nd_{0.8}FeO_3$	0.2:0.8:1:3	0.18	0.88	1
$La_{0.1}Nd_{0.9}FeO_3$	0.1:0.9:1:3	0.11	0.97	1

Table S11: EDS analysis results for the elemental composition of $La_xSm_{1-x}FeO_3$ (normalised to Fe)

Target material	Target La:Sm:Fe:O ratio	La	Sm	Fe
$La_{0.9}Sm_{0.1}FeO_3$	0.9:0.1:1:3	0.59	0.17	1
$La_{0.8}Sm_{0.2}FeO_3$	0.8:0.2:1:3	0.63	0.09	1
$La_{0.7}Sm_{0.3}FeO_3$	0.7:0.3:1:3	1.16	0.35	1
$La_{0.6}Sm_{0.4}FeO_3$	0.6:0.4:1:3	0.78	0.46	1
$La_{0.5}Sm_{0.5}FeO_3$	0.5:0.5:1:3	0.49	0.39	1
$La_{0.4}Sm_{0.6}FeO_3$	0.4:0.6:1:3	0.52	0.46	1
$La_{0.3}Sm_{0.7}FeO_3$	0.3:0.7:1:3	0.28	0.67	1
$La_{0.2}Sm_{0.8}FeO_3$	0.2:0.8:1:3	0.18	0.95	1
$La_{0.1}Sm_{0.9}FeO_3$	0.1:0.9:1:3	0.08	0.74	1

Table 12: EDS analysis results for the elemental composition of $La_xGd_{1-x}FeO_3$ (normalised to Fe)

Target material	Target La:Gd:Fe:O ratio	La	Gd	Fe
$La_{0.9}Gd_{0.1}FeO_3$	0.9:0.1:1:3	1.27	0.33	1
$La_{0.8}Gd_{0.2}FeO_3$	0.8:0.2:1:3	0.99	0.19	1
$La_{0.7}Gd_{0.3}FeO_3$	0.7:0.3:1:3	1.14	0.48	1
$La_{0.6}Gd_{0.4}FeO_3$	0.6:0.4:1:3	0.81	0.41	1
$La_{0.5}Gd_{0.5}FeO_3$	0.5:0.5:1:3	0.51	0.48	1
$La_{0.4}Gd_{0.6}FeO_3$	0.4:0.6:1:3	0.35	0.62	1
$La_{0.3}Gd_{0.7}FeO_3$	0.3:0.7:1:3	0.23	0.67	1
$La_{0.2}Gd_{0.8}FeO_3$	0.2:0.8:1:3	0.12	0.91	1
$La_{0.1}Gd_{0.9}FeO_3$	0.1:0.9:1:3	0.14	1.10	1

References

(1) Parida, S. C.; Rakshit, S. K.; Singh, Z. Heat capacities, order–disorder transitions, and thermodynamic properties of rare-earth orthoferrites and rare-earth iron garnets. *J. Solid State Chem.* **2008**, *181*, 101-121.

(2) Zhou, Z.; Guo, L.; Yang, H.; Liu, Q.; Ye, F. Hydrothermal synthesis and magnetic properties of multiferroic rare-earth orthoferrites. *J. Alloys Compd.* **2014**, *583*, 21-31.

(3) Marshall, L. G.; Cheng, J. G.; Zhou, J. S.; Goodenough, J. B.; Yan, J. Q.; Mandrus, D. G. Magnetic coupling between $Sm^{3+}$and the canted spin in an antiferromagnetic $SmFeO_3$ single crystal. *Phys. Rev. B.* **2012**, *86*, 064417.

(4) Treves, D. Studies on Orthoferrites at the Weizmann Institute of Science. *J. Appl. Phys.* **1965**, *36*, 1033-1039.

(5) White, R. L. Review of Recent Work on the Magnetic and Spectroscopic Properties of the Rare-Earth Orthoferrites. *J. Appl. Phys.* **1969**, *40*, 1061-1069.

(6) Lee, J. H.; Jeong, Y. K.; Park, J. H.; Oak, M. A.; Jang, H. M.; Son, J. Y.; Scott, J. F. Spin-canting-induced improper ferroelectricity and spontaneous magnetization reversal in $SmFeO_3$. *Phys. Rev. Lett.* **2011**, *107*, 117201.

(7) Gilleo, M. A. Magnetic Properties of a Gadolinium Orthoferrite, $GdFeO_3$, Crystal. *The Journal of Chemical Physics* **1956**, *24*, 1239-1243.

(8) Durbin, G. W.; Johnson, C. E.; Thomas, M. F. Temperature dependence of field-induced spin reorientation in $GdFeO_3$. *J. Phys. C: Solid State Phys.* **1977**, *10*, 1975-1978.

(9) J. D. Cashion, A. H. C., D. M. Martin, M. R. Wells. Magnetic interactions in gadolinium orthoferrite. *J. Phys. C: Solid State Phys.* **1970**, *41*, 1193-1194.

(10) Shao, M.; Cao, S.; Wang, Y.; Yuan, S.; Kang, B.; Zhang, J. Large magnetocaloric effect in $HoFeO_3$ single crystal. *Solid State Commun.* **2012**, *152*, 947-950.

(11) Bujkof, S.; Georgievt, D.; Krezhovt, K.; Nietzt, V.; Passage, G. Induced antiferromagnetism in $HoFeO_3$. *J. Phys.: Condens. Matter* **1995**, *7*, 8099-8107.

(12) Davidson, G. R.; Dunlap, B. D.; Eibschütz, M.; van Uitert, L. G. Mössbauer study of Yb spin reorientation and low-temperature magnetic configuration in $YbFeO_3$. *Phys. Rev. B.* **1975**, *12*, 1681-1688.
(13) Thirumalairajan, S.; Girija, K.; Hebalkar, N. Y.; Mangalaraj, D.; Viswanathan, C.; Ponpandian, N. Shape evolution of perovskite $LaFeO_3$ nanostructures: a systematic investigation of growth mechanism, properties and morphology dependent photocatalytic activities. *RSC Adv.* **2013**, *3*, 7549–7561.
(14) Zhang, Y.; Zhang, L.; Deng, J.; Dai, H.; He, H. Hydrothermal Fabrication and Catalytic Properties of $YBa_2Cu_3O_7$ Single Crystallites for Methane Combustion. *Catal. Lett.* **2010**, *135*, 126-134.
